# QSAR Reveals Decreased Lipophilicity of Polar Residues Determines the Selectivity of Antimicrobial Peptide Activity

**DOI:** 10.1021/acsomega.4c01277

**Published:** 2024-06-03

**Authors:** Mandelie van der Walt, Dalton S. Möller, Rosalind J. van Wyk, Philip M. Ferguson, Charlotte K. Hind, Melanie Clifford, Phoebe Do Carmo Silva, J. Mark Sutton, A. James Mason, Megan J. Bester, Anabella R. M. Gaspar

**Affiliations:** † Department of Biochemistry, Genetics and Microbiology, Faculty of Natural and Agricultural Sciences, University of Pretoria, Pretoria 0002, South Africa; ‡ Institute of Pharmaceutical Science, School of Cancer & Pharmaceutical Science, King’s College London, Franklin-Wilkins Building, 150 Stamford Street, London SE1 9NH, United Kingdom; § Antimicrobial Discovery Development and Diagnostics, Vaccine Evaluation and Development Centre, UK Health Security Agency, Salisbury SP4 0JG, United Kingdom; ∥ Department of Anatomy, Faculty of Health Sciences, University of Pretoria, Pretoria 0002, South Africa

## Abstract

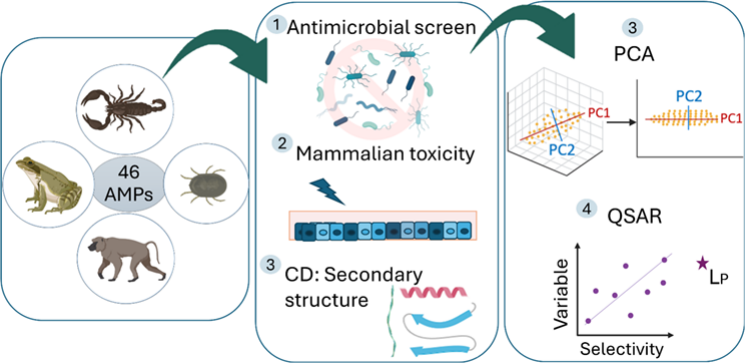

Antimicrobial resistance has increased rapidly, causing daunting morbidity and mortality rates worldwide. Antimicrobial peptides (AMPs) have emerged as promising alternatives to traditional antibiotics due to their broad range of targets and low tendency to elicit resistance. However, potent antimicrobial activity is often accompanied by excessive cytotoxicity toward host cells, leading to a halt in AMP therapeutic development. Here, we present multivariate analyses that correlate 28 peptide properties to the activity and toxicity of 46 diverse African-derived AMPs and identify the negative lipophilicity of polar residues as an essential physiochemical property for selective antimicrobial activity. Twenty-seven active AMPs are identified, of which the majority are of scorpion or frog origin. Of these, thirteen are novel with no previously reported activities. Principal component analysis and quantitative structure–activity relationships (QSAR) reveal that overall hydrophobicity, lipophilicity, and residue side chain surface area affect the antimicrobial and cytotoxic activity of an AMP. This has been well documented previously, but the present QSAR analysis additionally reveals that a decrease in the lipophilicity, contributed by those amino acids classified as polar, confers selectivity for a peptide to pathogen over mammalian cells. Furthermore, an increase in overall peptide charge aids selectivity toward Gram-negative bacteria and fungi, while selectivity toward Gram-positive bacteria is obtained through an increased number of small lipophilic residues. Finally, a conservative increase in peptide size in terms of sequence length and molecular weight also contributes to improved activity without affecting toxicity. Our findings suggest a novel approach for the rational design or modification of existing AMPs to increase pathogen selectivity and enhance therapeutic potential.

## 1 Introduction

Antimicrobial resistance is a global health crisis with a severe impact on developing countries,^1^ where infections due to drug resistance are difficult to treat, especially in patients with compromised immune systems.^2,3^ Additional costs of prolonged hospitalization and medical expenses have placed a strain on healthcare systems.^4^ This heightens the urgency in the search and development of new antimicrobial drugs, and antimicrobial peptides (AMPs) have emerged as promising drug candidates.

Cationic AMPs are found in the innate immune systems of a variety of living organisms. These peptides have bactericidal, fungicidal, antibiofilm, immunomodulatory, and anticancer properties^5−7^ identifying AMPs as multifunctional peptides. The broad-spectrum activity,^5,6,8^ superior pharmacodynamic properties,^7,9−12^ and multifaceted bactericidal mechanisms^12,13^ of AMPs are thought to lower the potential for drug resistance.

Despite these advantages, there are several drawbacks that hinder the breakthrough of AMPs into therapeutic use. Compared with antibiotics, AMPs tend to act against less specific targets, and this may cause a lack of selectivity.^14^ A side effect of this is the potential lysis of mammalian cell membranes causing hemolysis or cytotoxicity in humans.^15,16^ AMPs such as colistin, polymyxin B, and gramicidins have been approved for medical use but, due to toxic side effects, applications are limited.^14^ Thus, engineering AMPs capable of selectively targeting microbial over mammalian cells is an important consideration in AMP therapeutic development.

Rational design is a current approach used in recent studies aimed at developing new or optimizing natural AMPs to improve antimicrobial efficacy.^7,18^ Strategies include increasing the net positive charge to increase electrostatic interactions with microbial membranes, increasing peptide hydrophobicity to promote membrane insertion, or combining both strategies to increase peptide amphipathicity.^17^ While these strategies may enhance antimicrobial activity, microbial over mammalian selectivity is generally harder to address, leaving mammalian cell toxicity unresolved and hindering therapeutic application. Thus, for the successful rational design of effective and selective AMPs, it is essential to understand the sequence-driven features that confer not only target efficacy but also selectivity.

Molecular and experimentally determined properties aid in describing the relationship between the peptide structure and antimicrobial potency. In addition to charge and hydrophobicity, Krauson et al., Mai et al., and Li et al. consider peptide secondary structure and helicity as essential parameters for antimicrobial success,^18−20,62,63^ while others report AMP size and sequence length as important determinants.^17,21^ Due to the many parameters involved in the structure–activity relationships of AMPs, rational design, and optimization strategies often contain exceptions and contradictions, making it difficult to establish standard criteria.

The increase in information on AMP structure and function has contributed to the development of in silico chemoinformatic tools to analyze the structure–function relationships of peptides. This is useful in summarizing multidimensional data sets to find consistencies among variables.^22^ This approach can then be used to analyze the activity and selectivity of AMPs by focusing on AMP structure which in turn is defined by numerical physiochemical properties.^23^ Here, multivariate methods such as principal component analysis (PCA) and quantitative structure–activity relationship (QSAR) correlations are used to identify and then summarize the structural parameters of 46 AMPs with the aim of potentially simplifying future drug design and development.

Briefly, PCA reduces the dimensionality of a data set by transforming the input variables into reduced, linear groups called principal components (PCs).^24^ The PCs that show the largest variations indicate the relationships that exist in the multivariate data set, which were not initially observed in the input data. The first few PCs summarize the properties responsible for most of the variation.^24^ QSAR is a mathematical model that quantitatively relates a numerical measure of chemical structure e.g., a physiochemical property to a biological effect e.g., antimicrobial activity or toxicity.^25^ This type of analysis is gaining increased acceptance by medicinal researchers and related fields due to its ability to prioritize ideas in lead optimization and increase the rate of drug discovery.^26^

Although PCA analysis has not yet been featured regularly, QSAR multivariate methods have been used in previous studies involving AMPs. Ostberg and Kaznessis^27^ performed an extensive QSAR study on the antimicrobial, hemolytic, and cytotoxic effects of 62 AMPs derived from protegrins. The study included molecular descriptors that were correlated with activity against four Gram-negative, one Gram-positive bacteria, and one fungus. The complexity and vast amount of data made the results difficult to interpret without clear guidelines for future peptide design. Frecer^28^ quantitatively analyzed the antimicrobial and hemolytic activities of 97 cyclic AMPs, derived from Protegrin-1, by making use of simple additive molecular properties. QSAR modeling correlated antimicrobial potencies of cyclic peptides to charge and amphipathicity, while the lipophilicity of residues forming the nonpolar face correlated with increased hemolysis.^28^

The current study focuses on a new group of more diverse, linear AMPs that were identified in various African species. A total of 46 AMPs, of which 27 are novel or have unknown activity, were subjected to antimicrobial screening against a large panel of eight Gram-negative, four Gram-positive bacteria, and six fungi, including susceptible and resistant strains. Cytotoxicity was evaluated against human erythrocytes and immortalized keratinocytes. The secondary structures of each AMP in Tris buffer, as well as three different membrane-mimicking environments, were determined with circular dichroism (CD) spectroscopy. CD conformation together with molecular parameters such as charge, length, mass, lipophilicity, hydrophobicity, etc. of the diverse group of peptides allowed for conclusive, wide-spectrum PCA and QSAR analyses.

## 2 Materials and Methods

### 2.1 Peptide Selection and Modification

At first, 116 AMP candidates were identified in indigenous African species including scorpions, frogs, ticks, and primates using the Antimicrobial Peptide Database (APD3), the Data Repository of Antimicrobial Peptides (DRAMP), the Collection of Antimicrobial Peptides (CAMP_R3_) and other published sources. The list was reduced by selecting only short peptides of 25 amino acids or less in length to ensure cost-effective synthesis and screening. From large sequences, shorter sequences were identified using CAMP_R3_ prediction of antimicrobial regions within peptides. Amino acid sequences from 8 to 22 amino acids with increments of 2 amino acids were screened for predicted activity. Sequences with AMP prediction >0.95 (SVM, Random Forest, Discriminant Analysis) were selected and from these the shortest sequences with the highest charge (although not necessarily a predictor of activity), and not previously identified using DRAMP and APD3 were selected. This resulted in a total of 55 peptides. Furthermore, only the analogues derived from parent peptides with validated activity against *Escherichia coli* were included. Peptides that are under clinical investigation such as magainin^29^ or already extensively researched and/or patented (identified with DRAMP) were removed from the list. To increase positive charge, several of these peptides were amidated, resulting in the final 46 peptides (Table 1).

**Table 1:** Physiochemical Properties of 46 AMPs Derived from Sequences Identified in African Frog, Scorpion, Tick, and Primate Species^a^

	species	peptide	sequence	MW (g/mol)	length; charge	ElutionTime (min)^b^	*L*^c^	SCSA (Å)^d^	HBtot^e^
frogs	*Xenopus laevis* (Southern Africa)	XPF	GWASKIGQTLGKIAKVGLKELIQPK	2664	25; +4	45,8	7.85	1245,9	30
		XPF10a^N^	LGKIAKVGLK-NH_2_	1025	10; +4	22	3.76	511,8	9
		DRAMP02272a	GMASKAGAIAGKIAKVALKAL-NH_2_	1968	21; +5	44	7.62	658,2	15
		DRAMP02273a	KIAKVALKAL-NH_2_	1053	10; +4	28	4.38	472,6	9
		X1–20a^*,N^	KCLTLRSLKKTLKFCASGRT-NH_2_	2254	20; +7	31,7	6.70	1057,4	36
	*Xenopus petersii* (Southern Africa)	CPF-P2	GLASFLGKALKAGLKIGSHLLGGAPQ	2505	27; +3	50,5	12.39	1085,9	26
		CPF-P2a^N^	FLGKALKAGLKIGSHL-NH_2_	1652	16; +4	33	8.13	1085,9	26
		CPF-P3	GFGSFLGKALKAALKIGANVLGGAPQQ	2614	27; +3	54,3	11.62	1081,9	25
		CPF-P3–10a^N^	KALKAALKIG-NH_2_	1011	10; +4	22,7	3.16	421,5	9
		CPF-P3–12a^N^	KALKAALKIGAN-NH_2_	1196	12; +4	26	2.87	516,4	12
		CPF-P3–20a^N^	GKALKAALKIGANVLGGAPQ-NH_2_	1876	20; +4	35,5	6.60	729,2	20
	*Xenopus muelleri* (Central/Southern Africa)	CPF-MW1	GLGSLLGKAFKFGLKTVGKMMGGAPREQ	2879	28; +4	46,6	8.14	1279,3	33
		CPF-MW-10a^N^	KAFKFGLKTV-NH_2_	1137	10; +4	24,9	4.10	602,7	11
		CPF-MW-12a^N^	KAFKFGLKTVGK-NH_2_	1323	12; +5	25	3.11	693,4	14
		CPF-MW-20a^N^	GKAFKFGLKTVGKMMGGAPR-NH_2_	2081	20; +6	32,5	5.48	909,6	26
scorpions	*Parabuthus schlectheri* (Southern Africa)	PS–PB-8a^N^	WKSKLAKK-NH_2_	987	8; +5	14	0.26	560,9	15
		PS–PB-14a^N^	KKAWKSKLAKKLRAKGK-NH_2_	1968	17; +10	22	–2.39	994,1	32
		PS–PB-16a^N^	GSFLKKAWKSKLAKKL-NH_2_	1832	16; +7	28,8	3.74	981,4	23
		PS–PB-20a^N^	GSFLKKAWKSKLAKKLRAKG-NH_2_	2245	20; +9	29	2.05	1092,2	31
	*Opistophthalmus carinatus* (Southern Africa)	Opis16a^N^	GKVWDWIKSTAKKLWN-NH_2_	1959	16; +4	41,2	4.87	1134,9	14
		Opis8a^N^	WIKSTAKK-NH_2_	960	8; +4	15	1.61	513,5	24
	*Androctonus amoreuxi* (North Africa)	AamAP1	FLFSLIPHAIGGLISAFK	1931	18; +1	50	16.27	911,8	14
		AamAP-S1	FLFSLIPKAIGGLISAFK	1922	18; +2	52,3	15.15	926,8	15
		AamAP-S1a^N^	FLFSLIPKAIGGLISAFK-NH_2_	1921	18; +3	55	15.15	926,8	15
		AamAP1-Lys	FLFKLIPKAIKKLISKFK	2162	18; +6	45,5	10.92	1282,5	25
		AamAP1-Lys-a^N^	FLFKLIPKAIKKLISKFK-NH_2_	2162	18; +7	43	10.92	1282,5	25
		A3	FLFSLIRKAIGGLISAFK	1981	18; +3	53	13.42	926,8	15
		A3a^N^	FLFSLIRKAIGGLISAFK-NH_2_	1980	18; +4	58	13.42	926,8	15
	*Opisthacanthus madagascariensis* (Madagascar)	K3-IsCTa	ILKKIWEGIKSLF-NH_2_	1575	13; +3	46,8	7.54	918,7	14
		K7P8K11-IsCTa	ILGKIWKPIKKLF-NH_2_	1584	13; +5	38	9.60	960,8	18
		A1F5K8-IsCTa	ALGKFWEKIKSLF-NH_2_	1567	13; +3	46,1	6.04	854,5	14
ticks	*Ornithodoros savignyi* (Southern Africa)	Os^*^	KGIRGYKGGYCKGAFKQTCKCY	2460	22; +6	24,7	5.48	1169,5	37
		Osa^*^^,^^N^	KGIRGYKGGYCKGAFKQTCKCY-NH_2_	2459	22; +7	24,5	5.48	1169,5	37
		Os-C	KGIRGYKGGYKGAFKQTKY	2150	19; +6	21,8	0.86	1056,7	31
		Os(3–12)a	IRGYKGGYCK-NH_2_	1143	10; +4	17	2.27	531,1	17
		Os(11–22)a^*^	CKGAFKQTCKCY-NH_2_	1379	12; +4	21,3	4.75	676	22
		W3(Os-C)a	WWWKGIRGYKGGYKGAFKQTKY-NH_2_	2709	22; +6	31	7.61	1420	34
		W(Os-C)a^N^	WKGIRGYKGGYKGAFKQTKY-NH_2_	2336	20; +7	25	3.11	1177,8	32
	*Ornithodoros moubata* (Southern Africa)	OmDefB^*^^,^^N^	RGIRGYKGGYCTGRFKQTCKCY	2546	22; +6	25	5.39	1131,9	43
		OmDefB19^N^	RGIRGYKGGYTGRFKQTKY	2237	19; +6	20	0.77	1019,1	37
		W-OmC-Ca	WSGIRGYKGGYKGLFKQTNY-NH_2_	2323	20; +5	29,5	5.84	1227,1	31
		W-OmB-Ca^N^	WRGIRGYKGGYTGRFKQTKY-NH_2_	2422	20; +7	25	3.02	1140,2	38
primates	*Papio anubis*/*Papio ursinus* (Africa)	ThetaDefA^*,U^	RCVCTRGFC	1044	9; +2	22	5.87	380,3	18
		ThetaDefB^*,U^	RCVCRRGVC	1051	9; +3	20	4.60	334.1	21
		BTD11a^*^^,^^N^	RRGVCRCVCRR-NH_2_	1363	11; +6	16	2.01	413.5	31
		BTD15a^*,N^	RCVCRRGVCRCVCRR- NH_2_	1824	15; +7	20	5.30	579.5	40

a * – AMPs containing more than one cysteine residue were evaluated in their reduced forms throughout the study. N – Novel derivative. U – Unknown antimicrobial activity.

b This is the retention time for the major fraction collected during semipreparative HPLC of each peptide supplied at c 80% purity.

c The sum of the lipophilicity of the constituent amino acids of the peptides was determined from Frecer^28^ calculated using π_FP_ = log *P*_o/w_(a.a.) – log *P*_o/w_(Gly) (Suppl. Table S1).

d The sum of the side chain surface areas (SCSA) of the constituent amino acids of the peptides were determined from Frecer^28^ as calculated from the Connolly surfaces method^46^ (Suppl. Table S1).

e The sum of the hydrogen bond acceptors and donors calculated using in house scripts (Suppl. Table S1).

Frog peptides such as Xenoxin-1, DRAMP02272a, or the large Caerulein peptides of *Xenopus petersii* and *X. muelleri* previously showed promising antibacterial and antifungal activity.^30−32^ From these parent peptides, shorter sequences like DRAMP02273a, the xenopsin-precursor fragments (XPFs), or Caerulein-precursor fragments (CPFs) were derived and included in the study. For comparative purposes, some larger peptides were also included such as CPF-P2, CPF-P3, and CPF-MW1.

Scorpion peptides such as Opistoporin 1 or 2 (Opis1 or 2) and Parabutoporin (PBP) from the venom of the South-African *Opistophthalmus carinatus* or *Parabuthus schlechteri* species previously showed potent antibacterial activity, especially against Gram-negative bacteria.^33^ For this study, shorter derivatives with predicted activity such as Opis16a and Opis8a based on the sequence of Opis1 or PS-PB-8a, PS-PB-14a, PS-PB-16a and PS-PB-20a based on PBP, were included. Three synthetic derivatives of the broad-spectrum *Androctonus amoreuxi* scorpion peptide, AamAP1, namely, AamAP-S1, A3, and AamAP1-Lys^34−37^ as well as their novel amidated forms were included in the study. Lastly, derivatives such as K3-IsCTa, K7P8K11-IsCTa, and A1F5K11-IsCTa^38,39^ from the potent *Opisthacanthus madagascariensis* scorpion peptide, IsCT, were added to the list of 16 scorpion peptides.

Eleven AMPs screened for antimicrobial activity originate from African tick species such as *Ornithodoros savignyi* and *O. moubata*. Research by Ismail et al.^40^ showed anti-Gram-positive and -negative activity for the defensin-derived Os, Os(3–12), and Os(11–22) peptides with negligible erythrocyte hemolysis. Amidated forms of these derivatives were included in the current study. From *O. moubata* three defensins have been identified namely OmDefA, OmDefB, and OmDefC, with similar activities against Gram-positive bacteria.^41,42^ To increase antimicrobial activity, analogues with higher charges or those that are carboxy-amidated were included in this study.^43^ Trp residues, especially at the N-termini, are known to drive the interaction of an AMP with the lipid core of bilayers through hydrophobic forces and are likely also responsible for increased activity.^44^ Tryptophan residues were added to the N-termini of several tick-derived analogues, and amidated versions were included.

In their study on primate AMPs, Garcia et al.^45^ predicted 10 theoretical cyclic θ-defensins, BTD-1 to BTD-10. BTD-2 had the most promising antimicrobial activity in vitro. Shorter linear sequences BTD15a and BTD11a contained within the cyclic peptide sequence have been identified and amidated for this study. Two other derivatives, ThetaDefA and ThetaDefB, the nine residues on the N-terminus or the C-terminus of BTD-1, are included in this study with an aim to identify the active region of the parent peptide.

### 2.2 Peptide Synthesis and Purification

A total of 46 peptides were supplied by Cambridge Research Biochemicals (Cleveland, UK) at ∼80% purity. They were further purified using water/acetonitrile gradients in reverse-phase high-performance liquid chromatography (RP-HPLC) on a Waters SymmetryPrep C8, 7 mm, 19 × 300 mm column.

Crude peptides, made up to 10 mg/mL in 0.1% TFA (v/v) in ddH_2_O, were subjected to preparative RP-HPLC purification on an Agilent 1100 system, with elution in gradient. A gradient program at a flow rate of 8 mL/min and column temperature of 25 °C was used to achieve elution with solvent A (0.1% trifluoroacetic acid (TFA) in water (v/v)) and solvent B (0.1% TFA in 100% acetonitrile (ACN) (v/v)). The elution program was as follows: (1) At 0–73 min, 100% solvent A to 0% solvent B; (2) at 73–77 min, 40% solvent A to 60% solvent B; (3) at 77–93 min, 10% solvent A to 90% solvent B; (4) at 93–105 min, 100% solvent A to 0% solvent B. Data collected were the retention time of the major fraction for each peptide in minutes as RP-HPLC ElutionTime (ElutionTime). Absorbance was detected at 254 nm, and fractions were collected manually. The fractions collected were spun in a speed-vac to remove ACN and the contents lyophilized. After freeze-drying for 24 h the peptides were dissolved in 10% (v/v) acetic acid before being lyophilized for a second time and then stored at −20 °C.

### 2.3 Antimicrobial Activity against a Panel of Sensitive and Resistant Pathogens

Antimicrobial activity was evaluated against a panel of 18 pathogens consisting of eight Gram-negative strains (*E. coli* NCTC 12923, *E. coli* LEC001, *Pseudomonas aeruginosa* PAO1, *P. aeruginosa* NCTC 13437, *Acinetobacter baumannii* ATCC 17978, *A. baumannii* AYE, *Klebsiella pneumoniae* M6, *K. pneumoniae* NCTC 13368), four Gram-positive strains (*Staphylococcus aureus* ATCC 9144, *S. aureus* NCTC 13616, *S. aureus* USA300, *S. aureus* 1199B) and six fungi (*Candida auris* TDG 1912, *C. albicans* NCPF 8018, *C. krusei* NCPF 3876, *C. tropicalis* NCPF 8760, and *C. parapsilosis* NCPF 3209). Pathogens were grown in noncation adjusted Mueller Hinton broth (MHB, for bacteria) or Roswell Park Memorial Institute (RPMI 1640) + 2% glucose (for fungi) at 37 °C with 180 rpm shaking and maintained on solid media (tryptic soy agar for bacteria and Sabouraud dextrose agar for fungi). For an antibiogram, see Suppl. Table S2.

The 46 peptides were serially diluted (128 to 2 μg/mL) in media down polypropylene 96-well plates. Bacteria or fungi, diluted from an overnight culture to an OD_600_ of 0.01, were added to the serially diluted peptides at a 1:1 ratio resulting in a starting cell density of ∼5 × 10^5^ CFU/mL. Plates were incubated at 37 °C for 20 h, and the optical density was determined spectrophotometrically at 600 nm. Minimum inhibition concentrations (MICs) were determined from a minimum of three biological repeats and are reported in μg/mL as well as μM. The MIC is defined as the lowest concentration that resulted in pathogen growth of <0.1 above the background absorbance. The MIC_50_ or MIC_90_ is defined as the lowest peptide concentration that results in 50% or 90% growth inhibition over a 20 h treatment period, respectively.

### 2.4 In Vitro Hemolytic Activity

Potential erythrocyte hemolysis was tested by incubating serial dilutions of the AMPs in PBS with freshly collected erythrocytes at a ratio of 1:1 in 96-well polypropylene V-shape plates for 1 h at 37 °C. Control wells were treated with 0.1% Triton-X-100 to ensure complete lysis or PBS-only to represent no lysis. The erythrocytes were collected by centrifugation and the OD_550_ of the supernatant was measured using a plate reader. The percentage hemolysis was calculated as
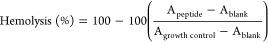
1where *A*_peptide_ is the absorbance value of erythrocytes exposed to a known peptide concentration, *A*_growth control_ is the absorbance of erythrocytes exposed to 0.1% Triton-X-100 and *A*_blank_ is the absorbance of erythrocytes exposed to the PBS. The concentrations at which a peptide caused 10% (HC_10_) and 50% (HC_50_) hemolysis was determined from the dose response curves.

### 2.5 In Vitro HaCat Cytotoxicity

The HaCat cell line (Cellonex, South Africa) was cultured in Dulbecco’s Modified Eagle Medium (DMEM) supplemented with 10% heat-inactivated fetal calf serum (FCS) and 1% antibiotic-antimycotic (Abs) and maintained at 5–10% CO_2_, 37 °C, 95% humidity. For cytotoxicity screening, 5.56 × 10^4^ cells/mL were plated in a 96-well plate. After 24 h incubation, the cells are initially exposed to each peptide at a concentration of 256 μg/mL or 0.1% Triton-X-100 as the control for 21 h. If cytotoxicity was observed, at a level of significance of *p* < 0.0001 compared with the untreated control after 21 h, a dose-response assay was undertaken. Cell viability was determined by adding 10% 3-(4,5-dimethyl-2-thiazolyl)-2,5-diphenyl-2H-tetrazolium bromide (MTT) (1 mg/mL), to each well and after a further 3 h incubation the media was discarded, and the formazan crystals dissolved by adding 25% DMSO in ethanol. The absorbance was measured at OD_570_ by using the FLUOstar Omega multidetection microplate reader (BMG Labtech). Percentage cell viability was calculated relative to the untreated control. The LC_50_ was defined as the concentration of AMP treatment that results in 50% lethality of HaCat cells and was determined from the dose-response curves.

### 2.6 Circular Dichroism Spectroscopy

Far-UV CD spectra of the peptides (50 μM) were obtained in Tris buffer (5 mM, pH 7.4), sodium dodecyl sulfate (SDS) micelles (50 mM prepared in 5 mM Tris, pH 7.4), and in the presence of small unilamellar vesicles (SUVs) using a Chirascan and a ChriscanPlus spectrometer (Applied Photophysics, Leatherhead, UK). For the preparation of SUVs, 1-palmitoyl-2-oleoyl-*sn*-glycero-3-phospho-(1′-rac-glycerol) (POPG) and 1-palmitoyl-2-oleoyl-*sn*-glycero-3-phosphoethanolamine (POPE) purchased from Avanti Polar Lipids, Inc. (Alabaster, AL) were used without any purification. The lipid powders were solubilized in chloroform and dried under rotor evaporation. To completely remove the organic solvent, the lipid films were left overnight under a vacuum and hydrated in 5 mM Tris buffer (pH 7.4). The lipid suspension was subjected to five rapid freeze–thaw cycles for further sample homogenization. POPE/POPG (75:25, mol:mol) and POPG SUVs were obtained by sonicating the lipid suspensions on Soniprep 150 (Measuring and Scientific Equipment, London, UK) for 2 × 5 min with an amplitude of six micrometers in the presence of ice to avoid lipid degradation. The SUVs were stored at 4 °C and used within 5 days of preparation.

CD spectra were recorded from 260 to 180 nm at a constant temperature of 296.15 K, a bandwidth of 2 nm, a step size of 1 nm, and a path length of 0.5 mm. The POPE/POPG or POPG SUV suspensions at a final concentration of 5 mM were used to dissolve the peptides to give a final peptide concentration of 50 μM. The same experimental conditions were used to investigate the peptide secondary structure in 5 mM Tris and 50 mM SDS micelles. For data processing, a spectrum of the peptide-free Tris solution, SDS solution, or lipid suspension was subtracted and Savitsky–Golay smoothing with a convolution width of 4 points was applied.

Peptide structures were quantitatively and qualitatively identified by examining the CD spectra and mean residue molar ellipticities (MRME, [θ], deg cm^2^ dmol^–1^). Peptides with a higher tendency to form a typical α-helical structure were identified by a positive band at ∼190 nm and two negative ellipticity minima around 208 and 222 nm^47,48^ (Table 2). Peptides with a tendency to form β-sheeted structures were identified by a positive ellipticity maximum at 195 nm and one negative ellipticity minimum at 215 nm. Similarly, a peptide with a positive ellipticity maximum at ∼205 nm and a minimum between 222–230 nm was identified to have a higher tendency to form a β-turn type I structure.^47^ Increased MRME intensity at the negative bands indicates a higher tendency of the peptide to form ordered structures. Lastly, CD spectra with no positive bands were identified as disordered structures.^49−51^

**Table 2:** CD Spectroscopy MRME Intensities of 46 African-Derived AMPs Elucidate the Predominant Secondary Structures Present in SDS, POPG, or POPG/POPE Liposomes^a^

species	peptide	SDS				POPG				POPG/POPE			
		λ of + [θ] (nm)^b^	[θ]_207–215_	[θ]_218–230_	predominant structure	λ of + [θ] (nm)^b^	[θ]_207–215_	[θ]_218–230_	predominant structure	λ of + [θ] (nm)^b^	[θ]_207–215_	[θ]_218–230_	predominant structure
frogs	XPF	191	–16087,3	–12730	α-helix	188	–15537,8	–15368,4	α-helix	191	–20857,8	–17786,7	α-helix
	XPF10a				disordered				disordered				disordered
	DRAMP02272a	191	–22372,2	–18563	α-helix	187	–16645,4	–18315,5	α-helix	190	–22234,8	–20072,1	α-helix
	DRAMP02273a	189	–10960,2	–5227,58	α-helix	188	–10823,6	–6096,34	α-helix	190	–12172,1	–5727,87	α-helix
	X1–20a	191	–11762,2	–10837,6	α-helix	197	–17718.7		β-sheet	193	–12131,2	–9901,79	α-helix
	CPF-P2	191	–11370,6	–9750,67	α-helix	189	–15114,6	–13831,7	α-helix	191	–27693,3	–24540,2	α-helix
	CPF-P2a	191	–10661,7	–7410,17	α-helix	186	–17674,1	–15410,2	α-helix	189	–14419,8	–11214,2	α-helix
	CPF-P3	192	–17908	–15106,1	α-helix	192	–15273,5	–13510,8	α-helix	192	–16291	–14066,2	α-helix
	CPF-P3-10a				disordered	197	–11718.3		β-sheet				disordered
	CPF-P3-12a				disordered	193	–4755.56		β-sheet				disordered
	CPF-P3-20a	191	–16959,1	–13504,2	α-helix	190	–17202	–14925,6	α-helix	191	–13974,2	–10927,9	α-helix
	CPF-MW1	191	–16090,9	–14897,4	α-helix	191	–17516,5	–17929	α-helix	190	–21024	–19769,6	α-helix
	CPF-MW-10a				disordered				disordered				disordered
	CPF-MW-12a				disordered	198	–3847.47		β-sheet				disordered
	CPF-MW-20a	191	–11091,2	–9544,57	α-helix	191	–3973.63		β-sheet	187	–11802,6	–8513,79	α-helix
scorpions	PS-PB-8a				disordered				disordered				disordered
	PS-PB-14a	191	–11466,1	–10672,9	α-helix	189	–14020	–11913,8	α-helix	189	–11898,7	–8431,4	α-helix
	PS-PB-16a	191	–12721,5	–7759,48	α-helix	189	–11801,8	–8608,36	α-helix	188	–12830,9	–7113,86	α-helix
	PS-PB-20a	191	–12815,3	–8142,95	α-helix	195	–12897,6	–9317	α-helix	191	–42170,4		P-II/α-helix
	Opis16a	192	–25651	–20167,6	α-helix	190	–20930,3	–15307,2	α-helix	188	–25929,2	–19734	α-helix
	Opis8a				disordered				disordered				disordered
	AamAP1	194	–9593,38	–9199,36	α-helix	192	–5013,64	–4705,86	α-helix	195	–4428,61	–4221,84	α-helix
	AamAP-S1	194	–12965,5	–12586,2	α-helix	192	–13047,3	–12862,1	α-helix	191	–13175,9	–13149,1	α-helix
	AamAP-S1a	193	–14571,1	–14745,9	α-helix	194	–14457,8	–14909,5	α-helix	194	–18872	–18677,2	α-helix
	AamP1-Lys	194	–14778,4	–14640,6	α-helix	190	–9234,91	–10738,3	α-helix	190	–12204,7	–11286,9	α-helix
	AamP1-Lysa	194	–14569,5	–14209,1	α-helix	195	–14491,8	–14824,9	α-helix	195	–13900,6	–13409,3	α-helix
	A3	193	–20899,3	–16918,5	α-helix	194	–18896,2	–20397	α-helix	191	–16406,4	–16536,2	α-helix
	A3a	192	–17674,8	–15326,7	α-helix	190	–6069,55	–6266,98	α-helix	194	–17351,6	–16210	α-helix
	K3-IsCTa	193	–14176,5	–9122,25	α-helix	190	–21973,5	–15284,4	α-helix	188	–20182	–14588,9	α-helix
	K7P8K11-IsCTa	194	–6578,13	–5694,38	α-helix	188	–6955,99	–7397,42	α-helix	189	–7209,07	–7240,37	α-helix
	A1F5K8-IsCTa	194	–12642,8	–7083,27	α-helix	188	–13386,5	–8670,74	α-helix	189	–14724,9	–10264,6	α-helix
ticks	Os	196	–2335,81	–1725,27	α-helix	189	–4350,35	–3789,7	α-helix	204		–2458,25	β-turn type I
	Osa	194	–4487,05	–4216,23	α-helix	193	–2114,6	–1968,74	α-helix	195	–2417,51	–2338,34	α-helix
	Os-C				disordered	197	–905.33		β-sheet				disordered
	Os(3–12)a				disordered				disordered				disordered
	Os(11–22)a	191	–6391,03		β-sheet	195	–7768,55		β-sheet	195	–6159,43		β-sheet
	W3(Os-C)a				disordered				disordered				disordered
	W(Os-C)a				disordered				disordered				disordered
	OmDefB				disordered				disordered				disordered
	OmDefB19				disordered				disordered				disordered
	W-OmC-Ca				disordered	192	–6508,76	–9435,85	α-helix				disordered
	W-OmB-Ca				disordered				disordered				disordered
primate	ThetaDefA				disordered				disordered				disordered
	ThetaDefB				disordered				disordered				disordered
	BTD11a				disordered				disordered				disordered
	BTD15a	185	–7204,69	–6479,47	α-helix	195	–1616.22		β-sheet	193	–6404.7		β-sheet

a All CD spectra are shown in Suppl. Figures S3–S6. MRME intensities are not calculated for CD spectra obtained in Tris buffer (5 mM) as all peptides adopted disordered conformations in this environment.

b Indicates the wavelength in nanometres where a positive MRME maximum is recorded.

### 2.7 PCA

Principal component analysis (PCA) is a method that reduces higher dimensional data to lower dimensional data while preserving the most important information. This is achieved by replacing the variables in a data set with a smaller number of derived variables.^52^ The goal of PCA was to extract a number of principal components (PCs) that can be used as clustering variables.

PCA was performed for all 46 peptides using JMP 17.0.0 software. The data matrix consisted of 46 samples (i.e., peptides) and 28 peptide molecular variables per pathogen group. Each column is centered and standardized individually, and PCs are calculated based on the Pearson correlation matrix. The overall antimicrobial potency of each peptide against each pathogen group (Gram-negative and -positive bacteria as well as fungi) was obtained by averaging the MIC values over the panel of strains tested.

To assess the correctness of PCA for the interpretation of the data sets, Bartlett’s test of sphericity was performed, where the null hypothesis (H_0_) states that there are no significant correlations between the variables in a data matrix, i.e., all correlation coefficients are <0.1 (Suppl. Table S13). This indicates that if the value for one variable is known, it cannot be used to predict the value of another variable. An alternative hypothesis (H_A_) is in contradiction to H_0_ stating that there is a correlation between variables. Thus, rejection of H_0_ would confirm the correctness of PCA application in this study.^55^

### 2.8 Quantitative Structure–Activity Relationship Analysis

Molecular descriptors used in the QSAR analysis included: charge, sequence length, overall lipophilicity (L), lipophilicity contributed by the polar or nonpolar residues (*L*_P_ and *L*_N_), summation of the side chain surface areas of the total peptide, polar or nonpolar residues (SCSA, SCSA_P_, and SCSA_N_), the molecular weight of the entire peptide, polar or nonpolar residues (Mw, MwP, and MwN), count of small lipophilic residues (CSL) or aromatic residues (CAR), the total number of hydrogen bond donor and acceptor centers (HBtot, HBdon, and HBacc), the total number of rotatable bonds (#RotBonds) and various amphipathicity descriptors (L_P_/L, L_P_/L_N_, L/L_N_, Q/L, Q/L_N_, SCSA_P_/SCSA_N_, MwP/MwN, Q/CSL, and Q/CAR) (Suppl. Table S1). Experimentally determined characteristics included ElutionTime as an indicator of overall peptide hydrophobicity and the CD structure of each peptide determined in POPG/POPE lipids representing Gram-negative membranes, POPG lipids for Gram-positive membranes, and SDS micelles for fungal membranes. AMPs with an α-helical structure were labeled 4, β-sheets were labeled 3, β-turns and mixed conformations were labeled 2, and disordered structures were labeled 1.

The molecular charge was calculated as the sum of qi, where qi is the formal charge of a residue at pH 7, which depends on the side chain p*K*_a_ constant.^53^ Lipophilicity parameters, L, L_P_, and L_N_, were defined by the residue side chain lipophilicity parameter, πFP, described by Frecer^28^ and Fauchere and Pliska,^54^ as a total of all the residues of the peptide (L) or only the contribution of the polar (P) or nonpolar (N) residues. The πFP parameter represents the difference between the experimental partitioning coefficients in the n-octanol/water system, log *P*_o/w_, of a given amino acid and glycine:
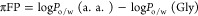
2

Lipophilicity parameters, L, L_P_, and L_N_, calculated with the more recent Wimley–White bilayer/water scale^76^ were also assessed to determine the robustness of the QSAR to different lipophilicity scales (Suppl. Table S15).

The surface areas of the peptide describe the bulkiness due to the residue side chains and were obtained from Frecer^28^ that used the Connolly surface calculation method.^46^ Molecular weights of the analogues (Mw, MwP, and MwN) were calculated as the sum of the relevant residue contributions. The count of CSL within the nonpolar face was also considered. Amphipathicity descriptors were defined as the ratio of certain molecular properties. Peptide flexibility was expressed as the total number of rotatable bonds in the residue side chains obtained with Shrodinger-Maestro version 13.1 released 2022-1. #RotBonds = sum of all the R.B.i, where R.B.i is the number of rotatable bonds in an amino acid side chain: R.B.i = R.B.(a.a.) - R.B.(Gly).

A Pearson correlation between the molecular descriptors and the average MIC per peptide was determined for Gram-negative, Gram-positive, and antifungal activity as well as cytotoxic activity against HaCat cells by using multivariate correlations in JMP 17.0.0 software and row-wise estimation. The Pearson product-moment correlation coefficient measures the strength of the linear relationship between the two variables. For response variables *X* and *Y*, it is denoted as *r* and computed as follows:
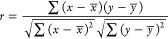
3

If there is an exact linear relationship between two variables, the correlation is 1 or −1, depending on whether the variables are positively or negatively related. If there is no linear relationship, then the correlation tends toward zero. In this study, the data matrix consisted of 46 samples (i.e., peptides) and the 28 molecular/experimental descriptors described earlier.

### 2.9 Activity Data and Statistical Analysis

Triplicate dose-response assays with three technical repeats each were performed per peptide against the target pathogens. All data analyses were performed using GraphPad Prism V 7.0 software (San Diego, CA, USA). The selectivity indices (SI) were determined by ratios of LC_50_/MIC. Statistical analyses were performed by determining Row totals/means with standard deviation (SD) or standard error of the mean (SEM) followed by One-way analysis of variance (ANOVA) with Dunnett’s multiple/selected comparison post-test.

## 3 Results

### 3.1 Novel African-Derived AMPs Show Promising Antimicrobial Activity In Vitro

AMP potency was evaluated against a panel of resistant and susceptible Gram-negative and Gram-positive bacteria by determining modal MICs (Suppl. Tables S3–S6), comparable to previous work where the potency of AMPs such as the WF peptides, Temporin B, Temporin L and Pleurocidin were determined against similar bacterial panels.^77−81^ Antifungal potency was evaluated against a panel of six fungi by measuring the modal MIC_50_ and MIC_90_ values (Suppl. Tables S7–S10). The EUCAST and CLSI definitions of an antifungal MIC value vary depending on the antifungal class, with amphotericin B having a 90% growth inhibition (MIC_90_) readout, while all other clinically used antifungals have a 50% growth inhibitory readout (MIC_50_). Overall, the peptides are more active against susceptible and resistant strains of *E. coli*, *A. baumannii*, *S. aureus*, and *C. tropicalis* NCPF 8760, requiring lower concentrations to inhibit these strains compared to others. *P. aeruginosa* PAO1 is the least sensitive bacterial strain as only seven AMPs have MICs ≤ 128 μg/mL. *C. auris* TDG 1912 is the least sensitive fungal strain as none of the peptides show MIC_90_ activity and only BTD11a shows MIC_50_ activity. Out of the 46 AMPs, 27 show strong antibacterial activity, and 22 show some antifungal activity, indicating a preference for prokaryotic cells. Noticeably, shorter peptides such as XPF10a, DRAMP02273a, CPF-P3-10a, CPF-P3-12a, CPF-MW-10a, CPF-MW-12a, PS-PB-8a, Opis8a, ThetaDefA, and ThetaDefB, with sequence lengths of 12 or less, have no activity.

For the frog-derived AMP group, nine out of 15 peptides have broad-spectrum antibacterial activity with MICs ≤ 128 μg/mL while only six have antifungal activity (Suppl. Tables S3 and S7). For this group, the antibacterial potential is greater than the antifungal potential. Three peptides, CPF-P2, CPF-P3, and CPF-MW1, show activity against all Gram-negative and Gram-positive bacteria except *P. aeruginosa* PAO1. XPF is the only frog AMP that shows antibacterial activity against the entire bacterial panel, including the *P. aeruginosa* strains (Suppl. Tables S3 and S3B). The bacterially active peptides inhibit only one or two fungal strains and require moderately high MICs to achieve fungal inhibition. Noticeably, peptides CPF-P2a and CPF-MW-20a show antibacterial activity against at least four bacterial strains but no antifungal activity.

The scorpion-derived group of peptides shows the highest activity. Thirteen of 16 peptides have MICs ≤ 128 μg/mL against bacteria or fungi (Suppl. Tables S4 and S8). Opis16a, AamAP-S1a, AamAP1-Lys, AamAP1-Lysa, A3, A3a, K3-IsCTa, K7P8K11-IsCTa, and A1F5K8-IsCTa all demonstrate potent activity against multiple different bacterial and fungal strains, with generally low MIC values. Opis16a, AamP1-Lys, AamP1-Lysa, and A3a are the most promising antibacterial peptides of the 46 AMPs with activity against all 12 bacterial strains with low MICs. The C-terminal amidation of parent peptides AamAP1-S1, AamAP1-Lys, and A3 results in peptides with increased overall activity.

The tick-derived AMP group has the lowest activity in the tested environments. Of the 11 AMPs, only four show significant antibacterial activity with MICs between 16 and 128 μg/mL and all peptides lack antifungal activity (Suppl. Tables S5 and S9). These results differ from previous studies that found activity for the Os peptides but could be explained by the previously described salt susceptibility of these peptides in different media.^40,61^ N-terminal tryptophan tagging and C-terminal amidation of parent peptides Os-C and OmDefB result in W3(Os-C)a, W(Os-C)a, W-OmB-Ca and W-OmC-Ca with increased potency against at least two bacterial strains but did not improve antifungal activity.

One of the four primate AMPs, BTD15a, shows promising antibacterial activity against all bacteria except the *P. aeruginosa* strains (Suppl. Tables S6 and S6B). BTD15a is also active against five of the six fungal species, with slightly higher concentrations (Suppl. Tables S10 and S10B). Under the conditions used BTD11a proved ineffective against bacteria but showed efficacy against all the tested fungal pathogens, making it the most promising antifungal AMP and the only peptide with activity against the resistant *C. auris* TDG 1912 strain (Suppl. Tables S10 and S10B).

### 3.2 Mammalian Toxicity Is Mostly Caused by the Microbially Active Scorpion-Derived AMPs

An ideal candidate for antimicrobial therapeutic applications must demonstrate selective activity against pathogens at low concentrations while exhibiting limited mammalian cell toxicity. To further characterize the potential of the 46 peptides, cytotoxicity was determined using human erythrocytes and the HaCat cell line. Erythrocyte hemolysis represents membrane lytic effects, while with the HaCat cell line, the effect on viability is determined and in addition to membrane effects includes the effect on cellular processes such as cellular metabolism, growth, and replication.

At the initial screening concentration of 256 μg/mL, 12 of the 46 AMPs caused hemolysis of more than 10% while eight caused 50% hemolysis at ≤256 μg/mL (Suppl. Table S11). Significant HaCat toxicity is observed for 20 AMPs and generally corresponds to those that showed hemolytic activity (Suppl. Figures S1 and S2). For the 12 most cytotoxic peptides (*p* < 0.0001), dose-response curves (1024–4 μg/mL) were generated from which the LC_50_ values were determined (Suppl. Table S12).

**Figure 1:**
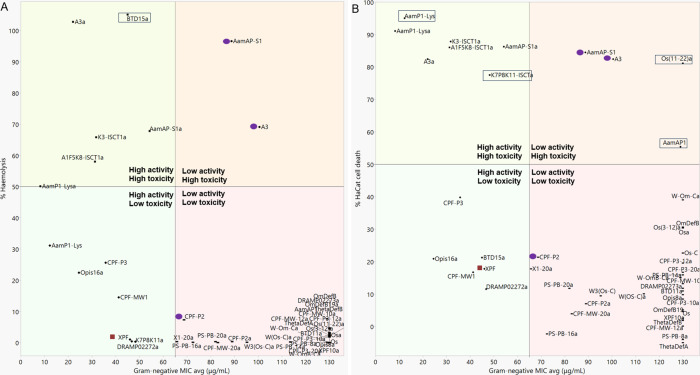
Peptide potency and selectivity shown by the relationship between the average Gram-negative MIC and (A) hemolysis or (B) HaCat toxicity both at 256 μg/mL. Boxed peptides show HaCat toxicity but not hemolysis or vice versa. Purple circle labeled peptides show increased selectivity for Gram-positive bacteria. Red square labeled peptides show increased selectivity for Gram-negative bacteria.

**Figure 2:**
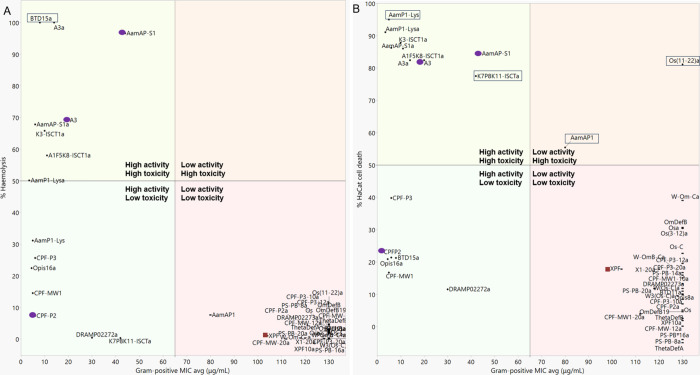
Peptide potency and selectivity shown by the relationship between the average Gram-positive MIC and (A) hemolysis or (B) HaCat toxicity both at 256 μg/mL. Boxed peptides show HaCat toxicity but not hemolysis or vice versa. Purple circle labeled peptides show increased selectivity for Gram-positive bacteria. Red square labeled peptides show increased selectivity for Gram-negative bacteria.

Overall, the frog peptides show low to moderate hemolytic activity and cytotoxicity indicating pathogen over mammalian cell selectivity (Suppl. Table S11, Figures S1 and S2). Most of these peptides show an average hemolysis of less than 10% with HC_10_ values larger than 256 μg/mL. Only CPF-P3 and CPF-MW1 show slightly higher hemolytic activities of more than 10% at 256 μg/mL, but their HC_50_ values remain above 256 μg/mL which is higher than their MICs. Similarly, HaCat viability remains at 80% or more after treatment with most of the frog peptides. CPF-P3 is the only cytotoxic frog peptide that causes a 39.9% reduction in HaCat cell viability with an average LC_50_ of 283.5 μg/mL (Suppl. Figure S2 and Table S12). This LC_50_ is still higher than the bacterial MICs of this peptide. Therefore, due to their bacterial potency and overall low cytotoxicity, the three most active frog AMPs, XPF, CPF-MW1, and CPF-P3 show noteworthy Gram-negative and -positive selectivity over mammalian cells (Figures 1 and 2).

The most active scorpion-derived AMP group shows the highest proportion of toxic peptides. Nine out of the 13 active peptides display an average hemolysis of more than 10% at 256 μg/mL while 10 peptides reduce HaCat cell viability by 44% or more (Suppl. Table S11, Figures S1 and S2). Peptides AamAP1-S1, AamAP1-S1a, A3, A3a, K3-IsCTa, and A1F5K8-IsCTa show the highest hemolysis with HC_50_ values lower than 256 μg/mL. The active scorpion AMPs also show HaCat toxicity with LC_50_ values ranging between 59.3 and 279.7 μg/mL (Suppl. Table S12). Peptides AamAP1-S1a and K3-IsCTa are the most toxic with the lowest LC_50_ values of 59.3 and 69.4 μg/mL, respectively (Suppl. Table S12). The four most active peptides, AamAP1-Lys, AamAP1-Lysa, A3a, and Opis16a have LC_50_ values of 124.4, 101.4, 279.7, and 311.2 μg/mL, respectively. AamAP1-Lysa and AamAP1-Lys are considered cytotoxic with LC_50_ < 256 μg/mL, while A3a shows less toxicity. Opis16a is regarded as the least toxic of the four most active scorpion peptides, making it the best candidate for potent bacterial activity and selectivity (Figures 1 and 2).

Peptides identified in African tick species cause significantly more HaCat toxicity than hemolytic activity (Suppl. Table S12, Figures S1 and S2). Six out of the 11 peptides, Osa, Os(3–12)a, Os(11–22)a, Os-C, W-OmC-Ca, and OmDefB, show significant decreases in HaCat cell viability compared to the growth control (Suppl. Figure S2). This is notable as the tick group shows little to no bactericidal, fungicidal, or hemolytic activity. Once again this can be attributed to the salt susceptibility of these peptides in different broth environments.^40,61^ Os(11–22)a is the most cytotoxic peptide in this group, reducing HaCat cell viability by 59.5% with an average LC_50_ of 163.2 μg/mL (Suppl. Table S12).

**Figure 3:**
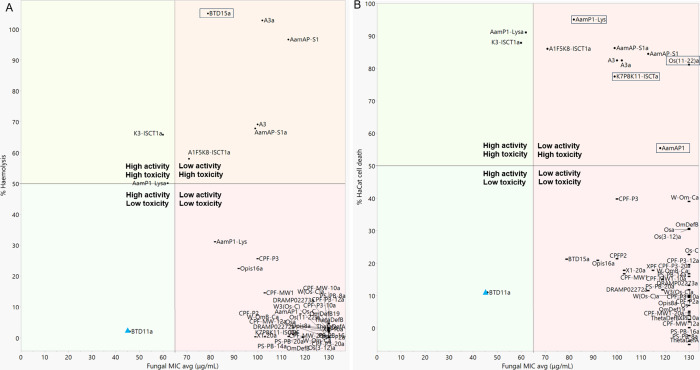
Peptide potency and selectivity shown by the relationship between the average antifungal MIC and (A) hemolysis or (B) HaCat toxicity both at 256 μg/mL. Boxed peptides show HaCat toxicity but not hemolysis or vice versa. Blue triangle labeled peptides show selectivity toward fungi only.

Lastly, only one out of the four primate-derived AMPs shows significant hemolysis (Suppl. Table S10 and Figure S1). BTD15a, the only peptide in this group with antibacterial activity, causes 100% hemolysis at 256 μg/mL. The HC_10_ and HC_50_ of this peptide is also the lowest of all 46 AMPs tested at 9 and 33 μg/mL, respectively. Although obtained under different conditions, these concentrations overlap with the MIC range of this peptide, suggesting little selectivity for pathogen cells over mammalian red blood cells (Figure 1 A). In terms of HaCat toxicity, at 256 μg/mL BTD15a only causes a 20% reduction in cell viability (Suppl. Figure S2). This shows that BTD15a is active toward pathogens and mammalian erythrocytes but not toward mammalian HaCat cells, suggesting some selectivity (Figure 1 B). The other three peptides in this group are considered noncytotoxic, causing on average less than 10% hemolysis or HaCat toxicity. Noticeably, the most active antifungal peptide, BTD11a, shows no hemolytic or cytotoxic activity and is the only peptide with pronounced selectivity for fungal rather than bacterial or mammalian cells (Figure 3).

### 3.3 CD Spectroscopy MRME Intensities Indicate Structured Secondary Conformations for Active AMPs

One molecular descriptor that is considered to play an important role in determining antimicrobial potency is the secondary structure of an AMP. AMPs tend to fold and form higher secondary structures when in close proximity to membranous environments.^50,51^ Mai et al.^62^ and Li et al.^63^ previously reported that peptides with more ordered secondary structures exhibit improved antibacterial potency. Here, far-UV CD analysis indicates that, in general, the peptides adopt a disordered conformation in Tris buffer (Suppl. Figures S3–S6). However, in three membrane-mimicking environments, the AMPs that have promising activity adopt predominantly α-helical or β-sheet conformations upon interaction with the SDS, Gram-positive, and -negative model membranes (Table 2 and Suppl. Figures S3–S6). This is consistent with previous studies that have shown that ordered secondary structures play a vital role in the membranolytic action of AMPs.^62,63^ A decrease in activity was also observed for shorter peptides that are unable to adopt stable secondary structures, possibly preventing the membranolytic action.

For 27 out of the 46 peptides, CD spectra are observed consistent with substantial α-helix conformation in the SDS environment: a positive band around 195 nm and two negative bands around 208 and 222 nm (Table 2). These peptides predominantly have longer sequences and correspond mostly to the 27 AMPs that showed activity against the bacterial panel. Only four peptides, DRAMP02273a, PS–PB14a, Os, and Osa, that have substantial α-helical content in SDS show no activity against bacteria. The activity of these peptides might have been affected by experimental conditions such as the salt concentration of the media used. These results are similar to what is reported by Pan et al.^64^ where an analogue of aurein 2.3 also adopts an α-helix similar to the active natural form, but in contrast, has no antimicrobial action.

On the other hand, peptides like W3(Os-C)a, W(Os-C)a, W-OmC-Ca, and W-OmB-Ca showed some activity against bacteria without adopting an ordered structure in SDS. This suggests that a structured secondary conformation such as an α-helix or β-sheet increases the probability of potent antimicrobial action but does not always guarantee activity. More likely, other physiological factors such as hydrophobicity, length, charge, or amphipathicity, in conjunction with secondary structure confer activity rather than secondary structure alone. The CD spectrum obtained for the most toxic tick AMP, Os(11–22)a, is consistent with β-sheet folding in the SDS environment, which is also observed in other membranous environments (Suppl. Figure S5E). Opis16a, one of the most potent and selective AMPs, showed the deepest MRME minima, indicating the strongest predominance for α-helicity of all 46 peptides in this environment.

In the POPG (Gram-positive) environment, peptides generally adopt more ordered structures with only 13 disordered peptides compared to 18 in SDS (Table 2). This can be explained by the greater size of POPG SUVs in contrast to SDS micelles therefore resulting in a stronger anionic attraction to the cationic peptides.^65^ As a result, peptides exhibit reduced flexibility when anchored in this membrane interaction. CD spectra of CPF-P3-10a, CPF-P3-12a, CPF-MW-12a, and Os-C are now consistent with β-sheet (Suppl. Figures S3I,J,N and S5C), with an increase in the intensity of a single negative band at 220 nm (Table 2). In POPG, W-OmC-Ca adopts a more α-helical instead of disordered conformation, with an increase in the intensity of two MRME minima recorded (Suppl. Figure S5J). Of the 27 α-helical AMPs in SDS, 25 remain α-helical in POPG, with X1-20a and CPF-MW-20a now showing stronger β-sheet tendencies (Suppl. Figure S3E,O and Table 2).

In the POPG/POPE (Gram-negative) environment, the structural tendencies of the peptides closely resemble those observed in SDS, rather than in POPG, with the same 18 peptides having disordered structures. This could be explained by the difference in charge between the SUVs. POPG is greatly anionic, whereas mixed POPG/POPE contains less anionic lipids and is more zwitterionic overall. As explained by Urushibara and Hicks,^66^ even slight differences in local charge density or hydrophobicity between membranes will result in different physiochemical surface properties that can dramatically affect the binding and consequently the conformational flexibility of AMPs. The 27 α-helical AMPs in SDS remain α-helical in POPG/POPE, except for PS-PB-20a which has a mixed P-II/α-helical conformation (Suppl. Figure S4D), BTD15a with stronger β-sheet tendencies and a loss in the 218–230 nm negative band (Suppl. Figure S6D) and Os with β-turn Type I tendencies with a loss in the 207–215 nm negative band (Figure S5A).

Generally, the less active tick and primate groups are mostly disordered, while the most active scorpion group showed the highest number of α-helical AMPs. Only two of the scorpion peptides, PS-PB-8a and Opis8a with short sequences of eight residues, remain disordered throughout all membrane environments, which explains the lack of activity. The same trends were observed for the frog- and primate-derived peptides. Active AMPs such as XPF, CPF-P2, CPF-P3, CPF-MW1, and BTD15a adopt predominantly α-helical conformations, whereas inactive shorter AMPs like XPF10a, CPF-MW10a, ThetaDefA and ThetaDefB are disordered.

Overall, the truncation of parent peptides to 15 amino acids or less resulted in decreased activity and a loss of secondary structure. This suggests that the optimal length for these AMPs to be able to adopt stable secondary structures and to exert their antimicrobial action is 16 amino acids or more. These findings are similar to those by Gagnon et al.^67^ and Liu et al.^68^ who also reported a decrease in antimicrobial action as the sequence length of the involved AMPs decreased.

Noticeably, six peptides from the tick or primate groups, Os, Osa, OmDefB, ThetaDefA, ThetaDefB, and BTD15a, contain two or more Cys residues (Suppl. Figures S5A,B,H and S6A,B,D). These residues can form disulfide bonds in oxidizing environments, which could result in different secondary structures than reported here.

### 3.4 Principal Component Analysis Highlights the Importance of Balance between Nonpolar and Polar Peptide Properties in Antimicrobial Activity

In this study, PCA is used to identify distinctive structural features in peptides that confer antimicrobial activity. Knowledge of these structural attributes can facilitate the design of novel antimicrobial agents and accelerate drug discovery. For PCA, 28 molecular peptide parameters were evaluated to identify specific structural features associated with Gram-negative, Gram-positive, or antifungal activities. The data matrices were composed of 46 peptides and 28 variables. Peptides were grouped according to their average MIC.

The Bartlett’s test of sphericity revealed χ^2^ is 2016.25 (PC-1) and 1643.94 (PC-2) for Gram-negative activity, 2004.10 (PC-1) and 1639.63 (PC-2) for Gram-positive activity, and 2037.89 (PC-1) and 1671.67 (PC-2) for antifungal activity (Suppl. Table S13). The *p* values calculated for the three populations are <0.0001 which is lower than the chosen significance level of 0.05 indicating that the data sets are suitable for factor analysis. Thus, there are no grounds for accepting H_0_ and it can be assumed that the correlation matrix is not an identity matrix. The adequate results for Bartlett’s test as well as the high number of peptides included in this study confirm the appropriate application of PCA for studying the main molecular parameters that affected antimicrobial activity.

Eigenvalues, percentages of variance, and cumulative variance were calculated for the parameters involved in peptide activity against Gram-negative and -positive bacteria as well as fungi (Suppl. Table S14). The number of principal components (PCs) specified for each group was determined based on the percentage cumulative variance.^56^ This agrees with other examples in the literature where the number of PCs is defined by a threshold cumulative variance.^52,56,57^ This specific threshold value depends on the specificity of the data and is usually at least 70%.^56^ For this study, the number of PC selections was determined at a threshold cumulative variance value of at least 80% (Suppl. Table S14).

For each group, four PCs are enough to describe over 80% of the cumulative variance. The four PCs result in 84.42% cumulative variance for activity against the Gram-negative bacteria, 83.95% for activity against the Gram-positive bacteria, and 84.82% for activity against fungi. The number of PCs selected for each group is also confirmed with a screen plot suggested by Kaiser’s criterion (Suppl. Figures S7–S9).^57^ The PCs with eigenvalues greater than 1.5 qualify to be used for the interpretation of results (Suppl. Table S14).

For anti-Gram-negative activity, PC-1 accounts for 37.9% and PC-2 for 29.36% of the variation (Suppl. Table S14) with the composition of individual PCs listed in Table 3. PC-1 is responsible for the highest amount of variability in the data indicating that the variables that make up PC-1 have the greatest impact on how the data are separated into active and nonactive peptides. PC-1 consists of 11 variables with absolute loading matrices of ≥0.7 (Table 3). Five of the variables show a negative correlation to PC-1 and relate mostly to the lipophilicity and nonpolar properties of peptides, including L, L_N_, ElutionTime, SCSA_N_, and CSL. Six of the variables show a positive correlation to PC-1 and include mostly the polar properties such as MW_P_, charge, MW_P_/MW_N_, Q/CSL, SCSA_P_/SCSA_N_, and Q/L_N_ (Table 3). PC-2 consists of four variables with positive loading matrices of ≥0.7. The size of the peptide (MW and length), or peptide side chains factors (SCSA and #RotBonds) have the largest impact on PC-2. L_P_ is the biggest contributor to PC-3 with a positive correlation of >0.7. PC-4 contains two amphipathic variables with L_P_/L having the biggest impact followed by Q/L (Table 3).

**Table 3:** Variable Composition of Each PC Obtained for Peptide Activity against Gram-Negative and Gram-Positive Bacteria and Fungi

principal component	Gram-negative		Gram-positive		fungi	
	variable	correlation coefficient	variable	correlation coefficient	variable	correlation coefficient
PC-1	L	–0.816	L	–0.808	L	–0.744
	L_N_	–0.768	L_N_	–0.758	HBdon	0.705
	ElutionTime	–0.752	ElutionTime	–0.750	HBtot	0.708
	SCSA_N_	–0.741	SCSA_N_	–0.734	MW_P_/MW_N_	0.718
	CSL	–0.700	MW_P_	0.715	charge	0.765
	MW_P_	0.716	charge	0.726	SCSA_P_	0.775
	charge	0.723	MW_P_/MW_N_	0.730	MW_P_	0.777
	MW_P_/MW_N_	0.734	Q/CSL	0.785	Q/CSL	0.786
	Q/CSL	0.780	SCSA_P_/SCSA_N_	0.850	SCSA_P_/SCSA_N_	0.861
	SCSA_P_/SCSA_N_	0.856	Q/L_N_	0.921	Q/L_N_	0.892
	Q/L_N_	0.925				
PC-2	#RotBonds	0.879	#RotBonds	0.878	MW_N_	0.754
	SCSA	0.932	SCSA	0.935	#RotBonds	0.808
	length	0.937	length	0.939	SCSA	0.916
	MW	0.972	MW	0.972	length	0.937
					MW	0.942
PC-3	L_P_	0.859	L_P_	0.865	L_P_	0.873
PC-4	L_P_/L	–0.828	L_P_/L	–0.828	L_P_/L	–0.836
	Q/L	0.797	Q/L	0.798	Q/L	0.809

**Figure 4:**
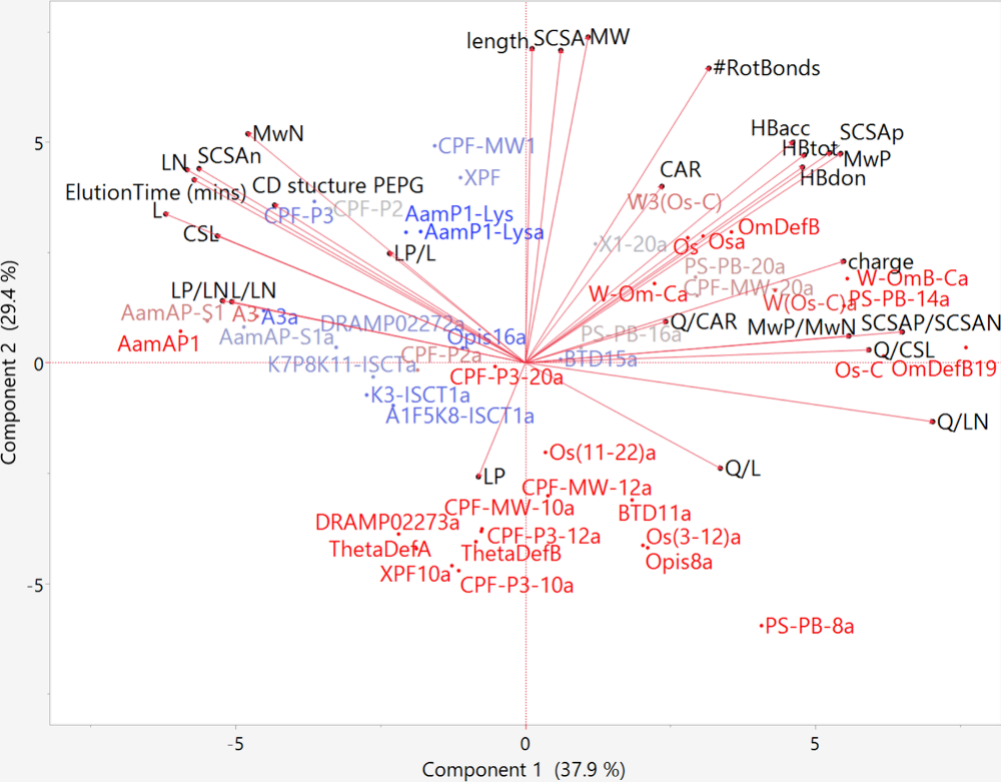
PCA biplot showing the molecular properties of AMPs that impact their MIC_avg_ against Gram-negative bacteria. A clustering of active (blue) AMPs are shown in the region of SCSA_N_, L_N,_ ElutionTime etc. while less active (red) AMPs cluster in regions of L_p_, SCSA_P_, HB, charge, SCSA_P_/SCSA_N_, Q/CSL etc.

On a PCA biplot for anti-Gram-negative activity, the 46 peptides are split into two distinct groups (Figure 4). Potent peptides that have lower MIC_avg_ are shown in blue and accumulate mostly to the upper left quadrant and are associated with molecular properties such as increased L_N_ residues, increased SCSA of nonpolar residues, and increased ElutionTime, indicating greater hydrophobicity, increased MW of nonpolar residues, increased CSL residues, and CD structure. Less active peptides with a higher MIC_avg_ accumulate mostly on the right. These peptides are generally less lipophilic, hydrophobic, and have fewer nonpolar residues. Inactive peptides are associated with molecular properties related to polarity such as increased L_P_, change in the peptide charge or the number of hydrogen bonds, increased SCSA of polar residues, and an increase in various amphipathic molecular descriptors.

The PC composition for anti-Gram-positive activity is very similar to that of anti-Gram-negative activity. PC-1 accounts for 37.4% and PC-2 for 29.5% of the variation within the data (Suppl. Table S14). PC-1 consists of ten peptide descriptors with absolute loading matrices of ≥0.7, similar to the PC-1 of the Gram-negative group (Table 3). Contributions to PC-1 include four negative correlations by L, L_N_, ElutionTime, and SCSA_N_ and 6 positive correlations by MW_P_, charge, MW_P_/MW_N_, Q/CSL, SCSA_P_/SCSA_N_, and Q/L_N_ (Table 3). The compositions of PC-2, -3, and -4 are also practically identical to the anti-Gram-negative PCs with peptide size and side chain (MW, length, SCSA, and #RotBonds), L_P_ and amphipathic variables making the greatest contributions, respectively (Table 3).

**Figure 5:**
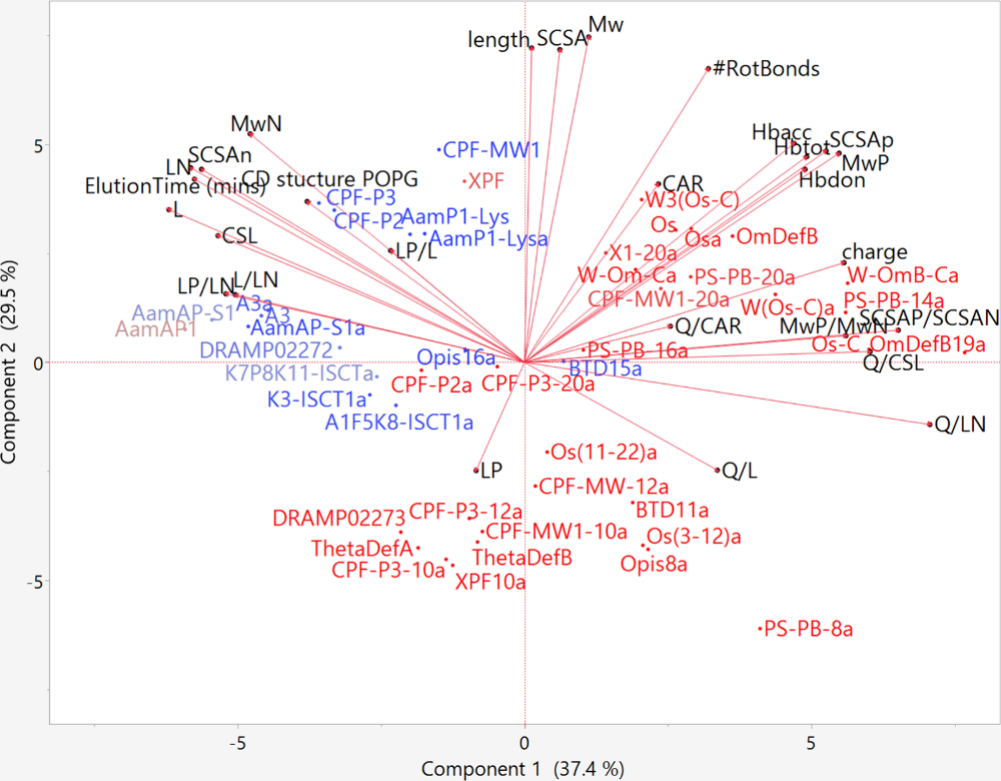
PCA biplot showing the molecular properties of AMPs that impact their MIC_avg_ against Gram-positive bacteria. A clustering of active (blue) AMPs are shown in the region of ElutionTime, SCSA_N_, L_N_ etc. while less active (red) AMPs cluster in regions of L_P_, Q/L, HB, charge, SCSA_P_/SCSA_N_ etc.

On the PCA biplot for anti-Gram-positive activity, the peptides are also split into two distinct groups (Figure 5). Potent peptides (blue) that have lower MIC_avg_ cluster mostly to the upper left quadrant associated with similar molecular properties as anti-Gram-negative activity such as increased lipophilicity, increased nonpolar residue SCSA, increased ElutionTime indicating greater hydrophobicity, increased CSL residues, and increased MW of nonpolar residues. The less active peptides (red) that have higher MIC_avg_ are scattered on the opposite side of the plot indicating decreased lipophilicity and hydrophobicity (Figure 5). The less active peptides are associated with molecular properties such as increased L_P_, change in peptide charge or number of HB, increased SCSA of polar residues, as well as an increase in amphipathic molecular descriptors like Q/L_N_.

For the PCA on both bacterial groups, peptides are categorized into active or inactive peptides first on their lipophilicity and nonpolar properties versus their polarity, then their size and side chain properties and last the lipophilicity of their polar residues and their amphipathic properties.

In contrast to the antibacterial activity, most of the AMPs are inactive against fungi or had high MIC_90_ values in the screenings, apart from BTD11a. Therefore, the composition and correlation coefficients for PC-1 of antifungal activity are different from those of antibacterial activity. PC-1 consists of ten peptide descriptors with absolute loading matrices of ≥0.7, with L being the only negative correlation (Table 3). Properties such as L_N_, ElutionTime, and SCSA_N_ that appear in the PC-1 of bacterial activity have lower than 0.7 negative correlation coefficients in the PC-1 of antifungal activity. Nine variables show a positive correlation to antifungal PC-1 and include mostly polar properties such as HBdon and HBtot, not associated with antibacterial PC-1, MW_P_/MW_N_, charge, SCSA_P_, MW_P_, Q/CSL, SCSA_P_/SCSA_N_, and Q/L_N_. The compositions of PC-2, -3, and -4 are more similar to those of antibacterial activity with the peptide size and side chain (MW, length, SCSA and #RotBonds), L_P_ and amphipathic variables making the greatest contributions, respectively (Table 3).

**Figure 6:**
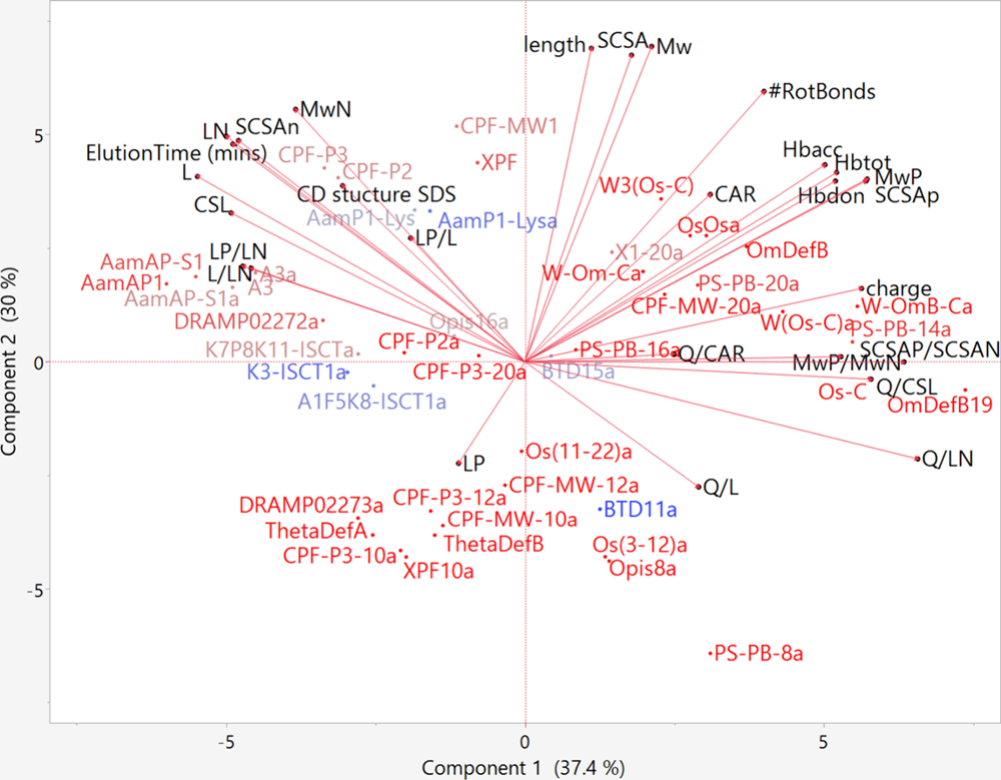
PCA biplot showing the molecular properties of AMPs that impact their MIC_avg_ against fungi. A clustering of active (light red/blue) AMPs is shown in different regions including L, CSL, L_P_/L_N_, L/L_N_ etc. while less active (red) AMPs cluster in regions of L_P_, HB, charge, SCSA_P_/SCSA_N_ etc.

On a PCA biplot, the most active antifungal AMPs, shown in blue, with MIC_90_ below 80 μg/mL do not group together or associate with any molecular property in particular (Figure 6). AMPs with some activity and MIC_90_ between 90 and 110 μg/mL group around the upper left quadrant in the vicinity of hydrophobic nonpolar molecular properties such as MW_N_, L, and CSL and amphipathic properties such as L_P_/L_N_ and L/L_N_. Those that are completely inactive, shown in bright red, are scattered in the other three quadrants associated with polarity such as L_P_ and charge (Figure 6).

### 3.5 QSAR Analysis Show That Certain Peptide Descriptors Including Lipophilicity and Size Can Be Manipulated To Achieve Broad-Spectrum Pathogen over Mammalian Selectivity

QSAR analysis was performed to relate the selectivity of the 46 AMPs to specific molecular properties that contribute to their potency and mechanism of action. Quantifying the structure–activity relationship of active peptides is essential for the future rational design of potent and selective antimicrobial peptides.

The MIC values were averaged over the panel of strains tested per group to facilitate the assessment of the overall antimicrobial potency of the peptides against Gram-negative bacteria, Gram-positive bacteria, or fungi. QSAR analysis that involves the averaged MIC (MIC_avg_) reflects the antimicrobial potency of a peptide better than analyses based on MICs against the individual strains, where the actual effect may be strongly dependent on the specific membrane composition, cell growth rate, and life cycle of the particular strain.^28^ The MIC_avg_ gives an overall indication of these factors and accentuates the role of the peptide sequence and structure in the achieved antimicrobial effect. Additionally, the development of a QSAR model for peptides that are active against a wide spectrum of Gram-negative and Gram-positive bacteria or fungi is preferable to structure–activity relationships described only for a specific individual strain. The relationships between sequences and observed bioactivities were analyzed in terms of physio-chemical molecular properties as well as experimentally determined characteristics of the peptides.

Table 4 shows the pairwise correlations between the averaged antimicrobial (MIC_avg_), hemolytic (% hemolysis), or cytotoxic (% HaCat cell death) activities and the respective molecular properties of the peptides. Consistent with the PCA analysis, descriptors such as L_N_, SCSA_N_, and ElutionTime show strong negative correlations with Gram-negative, Gram-positive, and antifungal activity (Table 4 and S15). This suggests that an increase in nonpolar lipophilic amino acids, nonpolar residues with bulkier side chains, or increased overall hydrophobicity leads to an increase in broad-spectrum antimicrobial activity. These three descriptors are also strongly correlated with hemolysis and HaCat cell death (Table 4) regardless of the lipophilicity scale used (Suppl. Table S15). This is consistent with several other studies^58−60^ which report that AMP lipophilicity and hydrophobicity closely correlate with mammalian toxicity. Thus, an increase in lipophilic nonpolar residues as well as overall hydrophobicity may lead to improved broad-spectrum activity but also decreased pathogen selectivity.

**Table 4:** Correlation of Single Molecular Descriptors of the 46 AMPs with Antimicrobial and Cytotoxic Activities

	property	*R*^2^				
		Gram-negative bacteria MIC_avg_	Gram-positive bacteria MIC_avg_	fungi MIC_avg_	%hemolysis	%HaCat cell death
1	ElutionTime (min)	–0.7203	–0.8032	–0.4590	0.5567	0.6485
2	L	–0.5246	–0.6713	–0.3576	0.5413	0.6376
3	L_N_	–0.7464	–0.8027	–0.5531	0.5855	0.6343
4	SCSA_N_	–0.7492	–0.7920	–0.4968	0.5230	0.6082
5	MwN	–0.6832	–0.7462	–0.4162	0.4500	0.4865
6	CD structure	–0.7250	–0.5670	–0.3425	0.4597	0.4548
7	L_P_/L_N_	–0.2103	–0.3396	–0.0510	0.3103	0.4426
8	L/L_N_	–0.1928	–0.3099	–0.0362	0.2763	0.4097
9	CSL	–0.5204	–0.5914	–0.3447	0.3155	0.2755
10	L_P_/L	–0.1358	–0.1675	–0.0890	0.1297	0.2489
11	SCSA	–0.4589	–0.3225	–0.1024	0.0341	0.2350
12	CAR	–0.0190	–0.0051	0.1287	0.0498	0.2250
13	Mw	–0.3691	–0.2877	–0.1111	0.0576	0.1195
14	sequence length	–0.3623	–0.3182	–0.0633	0.0297	0.0748
15	L_P_	0.5258	0.2956	0.4575	–0.0711	0.0330
16	#RotBonds	–0.3049	–0.1062	–0.0739	–0.0619	0.0321
17	Q/CAR	–0.0162	0.1919	0.1001	–0.2490	–0.1772
18	HBacc	0.0344	0.1853	0.0936	–0.2188	–0.2010
19	SCSA_P_	0.0653	0.2597	0.2801	–0.3813	–0.2144
20	HBtot	0.0447	0.1872	0.0260	–0.1794	–0.2305
21	HBdon	0.0486	0.1828	–0.0091	–0.1543	–0.2389
22	MwP	0.1253	0.2805	0.2178	–0.3133	–0.2675
23	Charge	0.0364	0.2892	0.0388	–0.2451	–0.2776
24	Q/L	0.2370	0.2561	0.1781	–0.1804	–0.2803
25	Q/CSL	0.3733	0.4251	0.3222	–0.3037	–0.3021
26	SCSA_P_/SCSA_N_	0.4631	0.5194	0.4496	–0.4156	–0.3158
27	MwP/MwN	0.4330	0.5075	0.3644	–0.3871	–0.3630
28	Q/L_N_	0.5264	0.6058	0.3810	–0.4363	–0.4634

Similar to the findings of the PCA analyses, the AMP secondary structure correlates strongly with Gram-negative activity with an *R*^2^ of −0.7250 surpassing the *R*^2^ of −0.5670 and −0.3425 for Gram-positive or antifungal activity, respectively. The more ordered the overall secondary structure of the peptide e.g., α-helical or β-sheeted, the lower the average MIC against Gram-negative bacteria. This indicates a narrow-spectrum association. Although the correlation is stronger toward Gram-negative activity, an association between CD structure and toxicity also exists with an *R*^2^ of 0.4597 for hemolysis and 0.4548 for HaCat toxicity. Therefore, an increase in the ordered secondary structure of an AMP can potentiate anti-Gram-negative activity but might also lead to an increase in toxicity.

Gram-positive antibacterial activity, in turn, is much more dependent on peptide lipophilicity and hydrophobicity, especially of the nonpolar residues, rather than the secondary structure. The *R*^2^ values for ElutionTime and L_N_ of >0.8 show the strongest correlations of all 28 molecular descriptors across the three pathogen groups. This highlights the importance of these descriptors for anti-Gram-positive activity. Another property that showed a relatively strong correlation with Gram-positive activity is the count of small lipophilic residues (CSL). This implies that increasing the count of small lipophilic residues in a peptide sequence or the overall hydrophobicity or lipophilicity of the nonpolar face will increase anti-Gram-positive activity but might contribute to toxicity.

Similarly, the antifungal activity is also more dependent on the lipophilicity and hydrophobicity of a peptide. Although, out of the 28 properties L_N_, SCSA_N_, and ElutionTime show the highest correlation with antifungal activity, the *R*^2^ values are much lower than the *R*^2^ values for antibacterial or HaCat activity and similar to the R^2^ values of hemolysis. This suggests that these properties might slightly increase the antifungal activity of a peptide but are not ideal in conferring fungal selectivity over bacterial or mammalian selectivity.

Thus, there are several properties that correlate well with antimicrobial activity showing an absolute *R*^2^ of >0.5, which can be manipulated to increase the potency of an AMP but will most likely also cause an undesired cytotoxic side-effect. Therefore, to increase pathogen over mammalian selectivity and reduce the risk of toxicity, descriptors that show little to no correlation with hemolytic or HaCat toxicity but some association with antimicrobial activity, and vice versa, should be considered. L_P_ (Table S1) describes the lipophilicity of those amino acids classified as polar (Arg, Asn, Asp, Gln, Glu, His, Lys, Ser, Thr, Tyr). As such it is possible to vary this parameter alongside, or independently of, a change in charge and potentially independently of overall hydrophobicity as nonpolar residues Cys, Ile, Leu, Met, Phe, Pro, Trp, and Val do not contribute. Most notably, L_P_ shows strong positive correlations with the MIC for all three pathogens and no association with toxicity. Therefore, including more polar cationic residues to reduce the lipophilicity of the polar groups will decrease the L_P_ resulting in an improved broad-spectrum MIC with no concomitant increase in cytotoxic effects. This result is also obtained when using the Wimley-White scale (Suppl. Table S15). For antibacterial activity, it is however important to retain a balance between L_P_ and L_N_. A drastic decrease in L_P_ to increase activity and selectivity can lead to an imbalance in the L_P_/L_N_ ratio, which could result in decreased activity. Thus, having a few L_N_ residues is important to balance activity and toxicity.

For antifungal activity, L_P_ is the only parameter that can be manipulated to increase activity and have no effect on toxicity. All other parameters that increase the antifungal activity also cause an increase in toxicity. Here, the L_P_/L_N_ ratio is not as important. Thus, reducing the lipophilicity contributed by the polar residues will decrease the overall L_P_ value and increase antifungal activity, with no effect on hemolytic or HaCat toxicity. Increasing the charge or the number of hydrogen bond-forming residues is also an effective strategy for decreasing HaCat toxicity and thus increasing antifungal selectivity.

The peptide size in terms of the sequence length and molecular weight also shows notable correlations with antibacterial activity and a negligible association with toxicity. This suggests that an increased sequence length and, consequently, Mw can lead to increased antibacterial activity and no change in toxicity. The same was observed in the MIC screens, where peptides shorter than 12 residues showed no antibacterial activity. Peptide size does not seem to be essential for the antifungal activity.

Lastly, the #RotBonds in an AMP sequence correlate with anti-Gram-negative activity and have no effect on toxicity. Thus, an increase in residues that contain higher amounts of side chain rotational bonds, such as Lys or Arg, will increase anti-Gram-negative activity without affecting cytotoxicity.

Other properties that show a correlation with toxicity but no association with antimicrobial activity can also be manipulated to reduce toxicity and achieve selectivity. One example is the count of aromatic residues (CAR) in a peptide sequence. This property correlates with HaCat cell death and somewhat with antifungal activity but shows no association with antibacterial activity. Hence, a decrease in the CAR could potentially reduce the HaCat toxicity of an AMP without affecting its antibacterial activity. Similarly, properties like charge and the number of hydrogen bonds (HB) a peptide can form correlate with both hemolysis and HaCat cell death but have no effect on Gram-negative or antifungal activity. Thus, increasing the overall charge and consequently the number of HB of an AMP would mean a decrease in the toxicity and an increase in Gram-negative or fungal selectivity. This, however, is not the case for Gram-positive antibacterial activity, as an increase in charge might lead to a loss in activity.

For a Gram-negative targeting AMP, an increase in polar residues with bulkier side chains would increase the SCSA_P_ value, which would decrease toxicity without affecting activity. For a fungal targeting AMP, a decrease in L_P_ would result in a decrease in the L_P_/L_N_ or L/L_N_ ratios, which can reduce the toxicity of the compound without affecting its antifungal potency.

## 4 Discussion

AMPs show great potential for development into therapeutic drugs to address the global antimicrobial resistance crisis. However, despite their many advantages, AMPs have some drawbacks that limit their translation into therapeutic drugs. Potent AMPs often exhibit low target selectivity and are accompanied by unacceptable toxicity toward host cells.^14−16^ More than 70 of the peptides deposited in DRAMP have entered the antimicrobial drug development stage with 27 in clinical trials and 34 in the preclinical stage.^69^ Thus far, only eight have made it to market including colistin, polymyxin B, vancomycin, gramicidin, bacitracin, daptomycin, enfuvirtide, and telaprevir, with many other trials being halted due to unwanted toxicity.^69,70^ Several clinically approved peptides including the well characterized Gramicidins and Polymyxins were initially denied due to their toxicity but have since been approved as last-resort therapeutics with limited applications.^14,69,70^ As a result, AMP toxicity is regarded as one of the main challenges to overcome in the design and development of peptides as therapeutic drugs.

The biological effects of AMPs are directly associated with their structural features, and it is therefore essential to understand the sequence-driven features that confer activity and, more importantly, target selectivity. Various physiochemical properties such as the peptide charge, hydrophobicity, amphipathicity, or secondary structure have been associated with structure–activity relationships (SAR) of AMPs.^17−20^ Due to the huge diversity in structural or physiochemical properties characteristic of AMPs, it is often difficult to pinpoint the exact molecular property or combination of properties required to obtain a potent and selective AMP for therapeutic use. Quantitative analyses of the SAR of AMPs could simplify large data sets to summarize the key physiochemical properties that determine selectivity; however, QSAR studies on AMP selectivity are scarce. Most available QSAR studies on AMPs utilize machine-learning techniques to define mathematical models that can predict AMP activity.^27,28,71,72^ Very few QSAR models explicitly address target selectivity and rarely define which physiochemical properties and amino acid residues contribute to selectivity.

In this study, a QSAR based on 28 molecular properties of 46 diverse African-derived AMPs, identifies L_P_ as an essential molecular parameter to consider for broad-spectrum antimicrobial selectivity. L_P_ showed a strong correlation with peptide activity against Gram-negative, Gram-positive, and fungal pathogen classes and a much lower correlation with mammalian cytotoxicity. This suggests that an increase in the number of polar residues to reduce the overall L_P_ of the peptide can lead to an increase in activity without affecting toxicity, thus increasing the selectivity of an AMP. Similar results were obtained using the Wimley-White scale, demonstrating the robustness of L_P_ as a parameter for selectivity regardless of the lipophilicity scale used.

The interaction with, and diffusion across, a pathogenic phospholipid-bilayer requires a peptide to be lipophilic and hydrophobic enough to interact with the lipid chains but also be polar enough to selectively target a pathogen over mammalian cell membranes. Several SAR studies show the importance of amphipathicity with a balance between hydrophobic and cationic regions in an active AMP sequence.^18−20,58,73^ A peptide with increased hydrophobicity or lipophilic nonpolar residues will show enhanced cytolytic activities, whereas a peptide with an imbalance in net charge will show decreased selectivity and antimicrobial potency.^58−60,73^ The QSAR model established by Frecer^28^ also correlated peptide lipophilicity and amphipathicity to potent antimicrobial action. This agrees with the current QSAR results that show a strong correlation between hydrophobicity or lipophilicity and peptide activity, but there is also a strong correlation with toxicity as well. Thus, increasing the overall hydrophobicity or lipophilicity of a peptide alone will improve activity but not selectivity. L_P_ represents a polar property that can be manipulated to balance lipophilicity and charge to achieve target selectivity.

In this study, three scorpion-derived peptides, namely, A3a, AamAP-Lysa, and Opis16a, and two frog-derived peptides, XPF and CPF-MW1 were identified as the best antibacterial peptides of the 46 AMPs with activity against all or all but one of the tested bacterial strains. Their activities correlate with their high overall hydrophobicity (ElutionTime) and lipophilicities (Table 1). Only Opis16a, XPF, and CPF-MW1 showed selective microbial killing and low cytotoxicity. This selectivity is correlated to low L_P_ values of −6.91 for Opis16a, −7.46 for XPF, and −7.26 for CPF-MW1. A3a and AamAP-Lysa with higher L_P_ values of −3.07 and −0.94, respectively, showed more toxicity and less pathogen selectivity.

Although a balance between hydrophobicity and polarity has been identified as a strategy to improve selectivity in previous SAR studies,^18−20,58,73^ manipulating the L_P_ of an AMP as a way of achieving this balance has, to our knowledge, not been suggested as of yet. At pH 7, there are four polar residues with negative L_P_ values including anionic Asp (L_P_ of −2.57) and Glu (L_P_ of −2.29), and cationic Arg (L_P_ of −1.01) and Lys (L_P_ of −0.99) (Suppl. Table S1). The cationic side chains of Arg or Lys have been shown to increase the selectivity for anionic pathogens rather than neutral mammalian membranes.^74^ Therefore, Arg and Lys fulfill both the cationic charge and negative L_P_ requirements making these residues the preferable choice to achieve pathogen over mammalian selectivity.

In the past, polar cationic residues were incorporated into peptide sequences to improve antimicrobial activity by increasing the overall charge; however, the QSAR shows that an increase in charge alone has no effect on Gram-negative or fungal activity of the peptide. Rather the increased charge caused by these residues reduces mammalian toxicity and decreases the L_P_ to improve microbial selectivity.

As an example, the usefulness of the present QSAR findings can be determined through the understanding of the results of an SAR study of a small group of AMPs by Fields et al.^75^ In their study, four linear peptides were derived from a parent SynSaf-P1 peptide using stepwise amino acid substitutions to improve antimicrobial activity. To obtain the first derivative, SynSaf-P8, a Thr residue was changed for a Lys resulting in an increased overall charge but also a very substantial drop in L_P_. Activity studies revealed that this increase in overall charge did not significantly alter the antimicrobial activity, consistent with the prediction of the current QSAR. However, further substitutions of Gly residues to more hydrophobic Trp residues, increasing L_N_, in derivatives SynSaf-P24, SynSaf-P56, and SynSaf-P96 significantly improved the antimicrobial activity. The current QSAR shows that AMPs with increased numbers of lipophilic and bulky nonpolar residues such as Trp or Phe result in increased hydrophobicity and generally display promising MIC results. As mentioned previously, a strong correlation between these molecular properties and peptide cytotoxicity also exists. An increase in overall hydrophobicity or lipophilicity would not only result in lower MICs but also lead to increased erythrocyte and HaCat toxicity. As predicted by the current QSAR, the improved SynSaf-P24, SynSaf-P56, and SynSaf-P96 derivatives with increased lipophilicity and hydrophobicity did not show increased toxicity.^73^ This is because of the initial incorporation of Lys which, on its own, did not improve the activity of SynSaf-P8, but did manage to cause a sufficient reduction in L_P_ to mitigate the toxicity, caused by the increased hydrophobicity and lipophilicity of the Trp residues in SynSaf-P24, SynSaf-P56, and SynSaf-P96, and achieve microbial over mammalian selectivity.

Although the most effective, L_P_ and charge are not the only descriptors involved in the antimicrobial selectivity. Other properties such as CAR, HB, #RotBonds and SCSA can be manipulated to achieve selectivity to specific pathogen groups over others as well as over mammalian cells. For example, decreasing the CAR in a peptide sequence could potentially reduce the HaCat toxicity without affecting its antibacterial activity. To further improve Gram-negative activity and selectivity the #RotBonds in the residues of a peptide sequence can be increased. Once again, Lys and Arg residues can be considered due to their long and flexible hydrocarbon side chains. For Gram-positive selectivity, a more balanced overall charge is preferred and may be achieved by including smaller, neutral lipophilic residues such as Ala, Gly, Leu, or Ile.^28^

Finally, peptide size in terms of sequence length and molecular weight can also be considered to improve peptide activity and selectivity, as shorter sequences show little activity. This could be due to the potential impact of peptide size on various other molecular properties, including the conformational state and flexibility of an AMP. A conservative increase in the sequence length and molecular weight would allow an increase in activity without affecting the toxicity.

Overall, the results show that the antimicrobial and cytotoxic effects of AMPs are proportional to their hydrophobicity and lipophilicity. Although this is well documented in the literature, the identification of properties such as L_P_, charge, hydrogen bonding, #RotBonds and CAR that confer selectivity to certain pathogen classes over mammalian or other pathogens represents a relatively novel finding. This may help overcome the major challenge of cytotoxic side effects of potent AMPs that restrict them from entering the drug development stage.

## 5 Conclusions

Analysis of 46 diverse peptides, with a focus on antimicrobial, hemolytic, and cytotoxic activity supplied the data required to obtain a better overall picture of what physiochemical properties are required for an effective and selective AMP. PCA and QSAR revealed that, at least for this sample of peptides, characteristics such as overall hydrophobicity, nonpolar lipophilic residues, and residue side chain surface area affect the antimicrobial and cytotoxic activity of an AMP. Some of these characteristics outweigh others, depending on the specific target pathogen. QSAR further identified the lipophilicity of polar residues to be the main molecular property that can be manipulated to improve pathogen over mammalian selectivity. Furthermore, an increase in the overall peptide charge specifically confers Gram-negative and fungal selectivity, while Gram-positive selectivity is obtained through small lipophilic residues. The PCA and QSAR results herein obtained grant valuable information for future rational AMP design strategies that could lead to antimicrobial and cytotoxic activity improvement.

## Supporting Information Available

Peptide properties per residue; antimicrobial activity; antibiogram; antibacterial activity; antifungal activity; hemolytic activity; cytotoxicity against HaCat cells; circular dichroism spectra; PCA; and QSAR -Wimley-White (PDF)

## Abbreviations

AMP: antimicrobial peptide
MIC: minimum inhibition concentration
PCA: principal component analysis
QSAR: quantitative structure–activity relationship

## References

[ref1] Ayukekbong J. A.; Ntemgwa M.; Atabe A. N. The threat of antimicrobial resistance in developing countries: causes and control strategies. Antimicrobial Resistance & Infection Control 2017, 6 (1), 1–8. 10.1186/s13756-017-0208-x. PMC5433038 28515903

[ref2] Jemal M.; Deress T.; Belachew T.; Adem Y. Antimicrobial resistance patterns of bacterial isolates from blood culture among HIV/AIDS patients at Felege Hiwot Referral Hospital, Northwest Ethiopia. International Journal of Microbiology 2020, 2020, 1–8. 10.1155/2020/8893266. PMC7593716 33133192

[ref3] Ioana D. O.; Evelina T.; Shunmay Y.; Rashida A. F.; Richard A. S.; Heidi H.; Alexander M. A.; Kranzer K. The association between antimicrobial resistance and HIV infection: a systematic review and meta-analysis. Clin. Microbiol. Infect. 2021, 27 (6), 846–853. 10.1016/j.cmi.2021.03.026. 33813126

[ref4] Nelson R. E.; Hyun D.; Jezek A.; Samore M. H. Mortality, length of stay, and healthcare costs associated with multidrug-resistant bacterial infections among elderly hospitalized patients in the United States. Clinical Infectious Diseases 2022, 74 (6), 1070–1080. 10.1093/cid/ciab696. 34617118 PMC8946701

[ref5] Amso Z.; Hayouka Z. Antimicrobial random peptide cocktails: a new approach to fight pathogenic bacteria. Chemical Communications (Camb) 2019, 55, 2007–2014. 10.1039/C8CC09961H. 30688322

[ref6] Lei J.; Sun L.; Huang S.; Zhu C.; Li P.; He J.; Mackey V.; Coy D. H.; He Q. The antimicrobial peptides and their potential clinical applications. Am. J. Transl. Res. 2019, 11 (7), 3919–3931. 31396309 PMC6684887

[ref7] Huan Y.; Kong Q.; Mou H.; Yi H. Antimicrobial peptides: Classification, design, application and research progress in multiple fields. Frontiers in Microbiology 2020, 11, 2559–2578. 10.3389/fmicb.2020.582779. PMC7596191 33178164

[ref8] Mahlapuu M.; Hakansson J.; Ringstad L.; Bjorn C. Antimicrobial peptides: An emerging category of therapeutic agents. Frontiers in Cellular and Infection Microbiology 2016, 6 (194), 1–12. 10.3389/fcimb.2016.00194. 28083516 PMC5186781

[ref9] Hancock R. E. W. Host defence (cationic) peptides: what is their future clinical potential?. Drugs 1999, 57, 469–473. 10.2165/00003495-199957040-00002. 10235687

[ref10] Mosca D. A.; Hurst M. A.; So W.; Viajar B. S. C.; Fujii C. A.; Falla T. J. IB-367, a protegrin peptide with in vitro and in vivo activities against the microflora associated with oral mucositis. Antimicrob. Agents Chemother. 2000, 44, 1803–1808. 10.1128/AAC.44.7.1803-1808.2000. 10858334 PMC89965

[ref11] El Shazely B.; Yu G.; Johnston P. R.; Rolff J. Resistance evolution against antimicrobial peptides in *Staphylococcus aureus* alters pharmacodynamics beyond the MIC. Frontiers in Microbiology 2020, 11 (103), 1–11. 10.3389/fmicb.2020.00103. 32117132 PMC7033599

[ref12] Yu G.; Baeder D. Y.; Regoes R. R.; Rolff J. Predicting drug resistance evolution: insights from antimicrobial peptides and antibiotics. Proc. Biol. Sci. 2018, 285 (1874), 20172687 10.1098/rspb.2017.2687. 29540517 PMC5879628

[ref13] Nguyen T. N.; Teimouri H.; Medvedeva A.; Kolomeisky A. B. Cooperativity in bacterial membrane association controls the synergistic activities of antimicrobial peptides. J. Phys. Chem. B 2022, 126 (38), 7365–7372. 10.1021/acs.jpcb.2c05345. 36108158

[ref14] Boto A.; Pérez De La Lastra J. M.; González C. C. The road from host-defense peptides to a generation of antimicrobial drugs. Molecules 2018, 23 (2), 311–320. 10.3390/molecules23020311. 29389911 PMC6017364

[ref15] Greco I.; Molchanova N.; Holmedal E.; Jenssen H.; Hummel B. D.; Watts J. L.; Hakkanson J.; Hansen P. R.; Svenson J. Correlation between hemolytic activity, cytotoxicity and systemic in vivo toxicity of synthetic antimicrobial peptides. Sci. Rep. 2020, 10 (1), 13206 10.1038/s41598-020-69995-9. 32764602 PMC7414031

[ref16] Sarkar T.; Chetia M.; Chatterjee S. Antimicrobial peptides and proteins: From nature’s reservoir to the laboratory and beyond. Frontiers in Chemistry 2021, 9, 1–40. 10.3389/fchem.2021.691532. PMC8249576 34222199

[ref17] Han Y.; Zhang M.; Lai R.; Zhang Z. Chemical modifications to increase the therapeutic potential of antimicrobial peptides. Peptides 2021, 146, 170666 10.1016/j.peptides.2021.170666. 34600037

[ref18] Krauson A. J.; Hall O. M.; Fuselier T.; Starr C. G.; Kauffman W. B.; Wimley W. C. Conformational fine-tuning of pore-forming peptide potency and selectivity. J. Am. Chem. Soc. 2015, 137, 16144–16152. 10.1021/jacs.5b10595. 26632653 PMC4697923

[ref19] Liu Y.; Du Q.; Ma C.; Xi X.; Wang L.; Zhou M.; Burrows J. F.; Chen T.; Wang H. Structure-activity relationship of an antimicrobial peptide, Phylloseptin-PHa: balance of hydrophobicity and charge determines the selectivity of bioactivities. Drug Design, Development and Therapy 2019, 13, 447–458. 10.2147/DDDT.S191072. 30774309 PMC6350648

[ref20] Duque H. M.; Rodrigues G.; Santos L. S.; Franco O. L. The biological role of charge distribution in linear antimicrobial peptides. Expert Opinion on Drug Discovery 2023, 18 (3), 287–302. 10.1080/17460441.2023.2173736. 36720196

[ref21] Park Y.; Park S. C.; Park H. K.; Shin S. Y.; Kim Y.; Hahm K. S. Structure-activity relationship of hp (2–20) analog peptide: Enhanced antimicrobial activity by n-terminal random coil region deletion. Biopolymers 2007, 88, 199–207. 10.1002/bip.20679. 17216635

[ref22] Iwaniak A.; Minkiewicz P.; Darewicz M.; Protasiewicz M.; Mogut D. Chemometrics and cheminformatics in the analysis of biologically active peptides from food sources. Journal of Functional Foods 2015, 16, 334–351. 10.1016/j.jff.2015.04.038.

[ref23] Scotti L.; Júnior F. J. B. M.; Ishiki H. M.; Ribeiro F. F.; Duarte M. C.; Santana G. S.; Oliveira T. B.; Formiga Melo Diniz M. F.; Quintans-Júnior L. J.; Scotti M. T. In Computer-aided drug design studies in food chemistry. Natural and artificial flavoring agents and food dyes; Grumezescu A. M.; Holban A. M., Eds.; Elsevier BV: Amsterdam, 2018; pp 261–297.

[ref24] Abdi H.; Williams L. J. Principal component analysis. Wiley Interdisciplinary Reviews: Computational Statistics 2010, 2 (4), 433–459. 10.1002/wics.101.

[ref25] Pittinger C.; Mohapatra A. Chapter 69 - Software tools for toxicology and risk assessment. In Information Resources in Toxicology (Fourth Edition); 2009; pp 631–638.

[ref26] Roy K.; Kar S.; Das R. N. Chapter 9 – Newer QSAR techniques. In Understanding the Basics of QSAR for Applications in Pharmaceutical Sciences and Risk Assessment; 2015; pp 319–356.

[ref27] Ostberg N.; Kaznessis Y. Protegrin structure-activity relationships: using homology models of synthetic sequences to determine structural characteristics important for activity. Peptides 2005, 26, 197–206. 10.1016/j.peptides.2004.09.020. 15629531

[ref28] Frecer V. QSAR analysis of antimicrobial and haemolytic effects of cyclic cationic antimicrobial peptides derived from protegrin-1. Bioorg. Med. Chem. 2006, 14, 6065–6074. 10.1016/j.bmc.2006.05.005. 16714114

[ref29] Jacob L.; Zasloff M. Potential therapeutic applications of magainins and other antimicrobial agents of animal origin. [Review]. Ciba Found. Symp. 1994, 186, 197–216. 10.1002/9780470514658.ch12. 7768152

[ref30] Lohner K.; Prossnigg F. Biological activity and structural aspects of PGLa interaction with membrane mimetic systems. Biochim. Biophys. Acta, Biomembr. 2009, 1778 (8), 1656–1666. 10.1016/j.bbamem.2009.05.012. 19481533

[ref31] Conlon J. M.; Mechkarska M. Host-defense peptides with therapeutic potential from skin secretions of frogs from the family Pipidae. Pharmaceuticals 2014, 7, 58–77. 10.3390/ph7010058. 24434793 PMC3915195

[ref32] King J. D.; Mechkarska M.; Coquet L.; Leprince J.; Jouenne T.; Vaudry H.; Takada K.; Conlon J. M. Host-defense peptides from skin secretions of the tetraploid frogs Xenopus petersii and *Xenopus pygmaeus*, and the octoploid frog *Xenopus lenduensis* (Pipidae). Peptides 2012, 33 (1), 35–43. 10.1016/j.peptides.2011.11.015. 22123629

[ref33] Cesa-Luna C.; Munoz-Rojas J.; Saab-Rincon G.; Baez A.; Morales-Garcia Y. E.; Juarez-Gonzalez V. R.; Quintero-Hernandez V. Structural characterization of scorpion peptides and their bactericidal activity against clinical isolates of multidrug-resistant bacteria. PLoS One 2019, 14 (11), e0222438 10.1371/journal.pone.0222438. 31710627 PMC6844485

[ref34] Almaaytah A.; Zhou M.; Wang L.; Chen T.; Walker B.; Shaw C. Antimicrobial/cytolytic peptides from the venom of the North African scorpion, *Androctonus amoreuxi*: Biochemical and functional characterization of natural peptides and a single site-substituted analog. Peptides 2012, 35, 291–299. 10.1016/j.peptides.2012.03.016. 22484288

[ref35] Almaaytah A.; Farajallah A.; Abualhaijaa A.; Al-Balas Q. A3, a scorpion venom derived peptide analogue with potent antimicrobial and potential antibiofilm activity against clinical isolates of Multi-Drug Resistant Gram-positive bacteria. Molecules 2018, 23, 1603–1615. 10.3390/molecules23071603. 30004427 PMC6100099

[ref36] Almaaytah A.; Abualhaijaa A.; Alqudah O. The evaluation of the synergistic antimicrobial and antibiofilm activity of AamAP1- Lysine with conventional antibiotics against representative resistant strains of both Gram-positive and Gram-negative bacteria. Infection and Drug Resistance 2019, 12, 1371–1380. 10.2147/IDR.S204626. 31213855 PMC6537036

[ref37] Song C.; Wen R.; Zhou J.; Zeng X.; Kou Z.; Zhang J.; Wang T.; Chang P.; Lv Y.; Wu R. Antibacterial and antifungal properties of a novel antimicrobial peptide GK-19 and its application in skin and soft tissue infections induced by *MRSA* or *Candida albicans*. Pharmaceutics 2022, 14 (9), 1937–1949. 10.3390/pharmaceutics14091937. 36145681 PMC9503518

[ref38] Lee K.; Shin S. Y.; Kim K.; Lim S. S.; Hahm K. S.; Kim Y. Antibiotic activity and structural analysis of the scorpion-derived antimicrobial peptide, IsCT, and its analogs. Biochem. Biophys. Res. Commun. 2004, 323 (2), 712–719. 10.1016/j.bbrc.2004.08.144. 15369808

[ref39] Acevedo I. C. C.; Silva P. I.; Silva F. D.; Araújo I.; Alves F. L.; Oliveira C. S.; Oliveira V. X. IsCT-based analogs intending better biological activity. Journal of Peptide Science: an Official Publication of the European Peptide Society 2019, 25 (12), e3219 10.1002/psc.3219. 31642159

[ref40] Ismail N. O.; Odendaal C.; Serem J. C.; Stromstedt A. A.; Bester M. J.; Sayed Y.; Neitz A. W. H.; Gaspar A. R. M. Antimicrobial function of short amidated peptide fragments from the tick-derived OsDef2 defensin. Journal of Peptide Science 2019, 25, 1–9. 10.1002/psc.3223. 31713951

[ref41] Nakajima Y.; Van Der Goes Van Naters-Yasui A.; Taylor D.; Yamakawa M. Two isoforms of a member of the arthropod defensin family from the soft tick *Ornithodoros moubata* (Acari: Argasidae). Insect Biochem. Mol. Biol. 2001, 31, 747–751. 10.1016/S0965-1748(01)00066-2. 11378409

[ref42] Nakajima Y.; Van Der Goes Van Naters-Yasui A.; Taylor D.; Yamakawa M. Antibacterial peptide defensin is involved in midgut immunity of the soft tick, *Ornithodoros moubata*. Insect Mol. Biol. 2002, 11, 611–618. 10.1046/j.1365-2583.2002.00372.x. 12421419

[ref43] Dennison S. R.; Harris F.; Bhatt T.; Singh J.; Phoenix D. A. The effect of C-terminal amidation on the efficacy and selectivity of antimicrobial and anticancer peptides. Molecular and cellular biochemistry 2009, 332 (1–2), 43–50. 10.1007/s11010-009-0172-8. 19513817

[ref44] Pasupuleti M.; Chalupka A.; Morgelin M.; Schmidtchen A.; Malmsten M. Tryptophan end-tagging of antimicrobial peptides for increased potency against *Pseudomonas aeruginosa*. Biochim. Biophys. Acta 2009, 1790, 800–808. 10.1016/j.bbagen.2009.03.029. 19345721

[ref45] Garcia A. E.; Osapay G.; Tran P. A.; Yuan J.; Selsted M. E. Isolation, synthesis, and antimicrobial activities of naturally occurring theta-defensin isoforms from baboon leukocytes. Infect. Immun. 2008, 76 (12), 5883–5891. 10.1128/IAI.01100-08. 18852242 PMC2583559

[ref46] Connolly M. L. Analytical molecular surface calculation. J. Appl. Crystallogr. 1983, 16, 548–558. 10.1107/S0021889883010985.

[ref47] Kelly S. M.; Jess T. J.; Price N. C. How to study proteins by circular dichroism. Biochimica et Biophysica Acta - Proteins Proteomics 2005, 1751 (2), 119–139. 10.1016/j.bbapap.2005.06.005. 16027053

[ref48] Rogers D. M.; Jasim S. B.; Dyer N. T.; Auvray F.; Réfrégiers M.; Hirst J. D. Electronic circular dichroism spectroscopy of proteins. Chemistry 2019, 5 (11), 2751–2774. 10.1016/j.chempr.2019.07.008.

[ref49] Baldwin R. L.; Rohl C. A. Deciphering rules of helix stability in peptides. Methods Enzymol. 1998, 295, 1–26. 10.1016/S0076-6879(98)95032-7. 9750211

[ref50] Fairlie D. P.; Shepherd N. E.; Hoang H. N.; Abbenante G. Single turn peptide alpha helices with exceptional stability in water. J. Am. Chem. Soc. 2005, 127 (9), 2974–2983. 10.1021/ja0456003. 15740134

[ref51] Wang D.; Cheng K.; Kulp J. L.; Arora P. S. Evaluation of biologically relevant short alpha-helices stabilized by a main-chain hydrogen-bond surrogate. J. Am. Chem. Soc. 2006, 128 (28), 9248–9256. 10.1021/ja062710w. 16834399 PMC1828873

[ref52] Iwaniak A.; Hrynkiewicz M.; Bucholska J.; Darewicz M.; Minkiewicz P. Structural characteristics of food protein-originating di- and tripeptides using principal component analysis. European Food, Research and Technology 2018, 244, 1751–1758. 10.1007/s00217-018-3087-3.

[ref53] Dawson R. M. C.; Elliott D. C.; Elliott W. H.; Jones K. M. Data for Biochemical Research, 3rd ed.; Oxford Science Publications: 1986; pp 1–31.

[ref54] Fauchere J. L.; Pliska V. Hydrophobicity scale (pi-r). Eur. J. Med. Chem. 1983, 18, 369–375.

[ref55] Bartlett M. S. A note on multiplying factors for various chi-squared approximations. Journal of the Royal Statistical Society: Series B 1954, 16, 296–298. 10.1111/j.2517-6161.1954.tb00174.x.

[ref56] O’rourke N.; Hatcher L.; Stepanski E. J. Principal component analysis. In A step-by-step approach to using SAS for univariate and multivariate statistics, 2nd ed.; O’Rourke N.; Hatcher L.; Stepanski E. J. *Eds.;* SAS Institute: Cary, 2005; pp 453–455.

[ref57] Stanisz A. Statistical course with an application of STATISTICA PL on medicine examples. In Principal component analysis; Stanisz A. Ed.; StatSoft Cracow: Poland, 2007; pp 165–181.

[ref58] Gong H.; Zhang J.; Hu X.; Li Z.; Fa K.; Liu H.; Waigh T. A.; Mcbain A.; Lu J. R. Hydrophobic control of the bioactivity and cytotoxicity of de novo designed antimicrobial peptides. ACS Appl. Mater. Interfaces 2019, 11 (38), 34609–34620. 10.1021/acsami.9b10028. 31448889

[ref59] Frecer V.; Ho B.; Ding J. L. De novo design of potent antimicrobial peptides. Antimicrob. Agents Chemother. 2004, 48 (9), 3349–3357. 10.1128/AAC.48.9.3349-3357.2004. 15328096 PMC514781

[ref60] Yeaman M. R.; Yount N. Y. Mechanisms of antimicrobial peptide action and resistance. Pharmacol. Rev. 2003, 55, 27–55. 10.1124/pr.55.1.2. 12615953

[ref61] Mbuayama K. R.; Taute H.; Strömstedt A. A.; Bester M. J.; Gaspar A. R. M. Antifungal activity and mode of action of synthetic peptides derived from the tick OsDef2 defensin. J. Pept. Sci. 2022, 28 (5), e3383 10.1002/psc.3383. 34866278

[ref62] Mai X. T.; Huang J.; Tan J.; Huang Y.; Chen Y. Effects and mechanisms of the secondary structure on the antimicrobial activity and specificity of antimicrobial peptides. Journal of Peptide Science: an official publication of the European Peptide Society 2015, 21 (7), 561–568. 10.1002/psc.2767. 25826179

[ref63] Li S.; Wang Y.; Xue Z.; Jia Y.; Li R.; He C.; Chen H. The structure-mechanism relationship and mode of actions of antimicrobial peptides: A review. Trends in Food ScienceTechnology 2021, 109, 103–115. 10.1016/j.tifs.2021.01.005.

[ref64] Pan Y. L.; Cheng J. T.; Hale J.; Pan J.; Hancock R. E.; Straus S. K. Characterization of the structure and membrane interaction of the antimicrobial peptides aurein 2.2 and 2.3 from Australian southern bell frogs. Biophys. J. 2007, 92 (8), 2854–2864. 10.1529/biophysj.106.097238. 17259271 PMC1831713

[ref65] Wojciechowska M.; Miszkiewicz J.; Trylska J. Conformational changes of Anoplin, W-MreB1–9, and (KFF)3K peptides near the membranes. International Journal of Molecular Sciences 2020, 21 (24), 9672 10.3390/ijms21249672. 33352981 PMC7766051

[ref66] Urushibara T.; Hicks R. Effect of liposome surface charge and peptide side chain charge density on antimicrobial peptide-membrane binding as determined by circular dichroism. J. Membr. Sci. Technol. 2013, 3 (3), 124 10.4172/2155-9589.1000124.

[ref67] Gagnon M.; Strandberg E.; Grau-Campistany A.; Wadhwani P.; Reichert J.; Bürck J.; Rabanal F.; Auger M.; Paquin J. F.; Ulrich A. S. Influence of the length and charge on the activity of alpha-helical amphipathic antimicrobial peptides. Biochemistry 2017, 56 (11), 1680–1695. 10.1021/acs.biochem.6b01071. 28282123

[ref68] Liu Z.; Brady A.; Young A.; Rasimick B.; Chen K.; Zhou C.; Kallenbach N. R. Length effects in antimicrobial peptides of the (RW)n series. Antimicrob. Agents Chemother. 2007, 51 (2), 597–603. 10.1128/AAC.00828-06. 17145799 PMC1797765

[ref69] Wang C.; Hong T.; Cui P.; Wang J.; Xia J. Antimicrobial peptides towards clinical application: Delivery and formulation. Adv. Drug Delivery Rev. 2021, 175, 113818 10.1016/j.addr.2021.05.028. 34090965

[ref70] Mahlapuu M.; Björn C.; Ekblom J. Antimicrobial peptides as therapeutic agents: opportunities and challenges. Critical Reviews in Biotechnology 2020, 40 (7), 978–992. 10.1080/07388551.2020.1796576. 32781848

[ref71] Cardoso M. H.; Orozco R. Q.; Rezende S. B.; Rodrigues G.; Oshiro K. G. N.; Cândido E. S.; Franco O. L. Computer-aided design of antimicrobial peptides: Are we generating effective drug candidates?. Frontiers in Microbiology 2020, 10, 3097 10.3389/fmicb.2019.03097. 32038544 PMC6987251

[ref72] Toropova M. A.; Aleksandar M. V.; Veselinović J. B.; Stojanović D. B.; Toropov A. A. QSAR modeling of the antimicrobial activity of peptides as a mathematical function of a sequence of amino acids. Comput. Biol. Chem. 2015, 59 (Pt A), 126–130. 10.1016/j.compbiolchem.2015.09.009. 26454621

[ref73] Hwang P. M.; Vogel H. J. Structure-function relationships of antimicrobial peptides. Biochemistry and Cell Biology 1998, 76 (2), 235–246. 10.1139/bcb-76-2-3-235. 9923692

[ref74] De Planque M. R. R.; Kruijtzer J. A. W.; Liskamp R. M. J.; Marsh D.; Greathouse D. V.; Koeppe R. E.; De Kruijff B.; Killian J. A. Different membrane anchoring positions of tryptophan and lysine in synthetic transmembrane α-helical peptides. J. Biol. Chem. 1999, 274, 20839–20846. 10.1074/jbc.274.30.20839. 10409625

[ref75] Fields F. R.; Manzo G.; Hind C. K.; Janardhanan J.; Foik I. P.; Do Carmo Silva P.; Balsara R. D.; Clifford M.; Vu H. M.; Ross J. N.; Kalwajtys V. R.; Gonzalez A. J.; Bui T. T.; Ploplis V. A.; Castellino F. J.; Siryaporn A.; Chang M.; Sutton J. M.; Mason A. J.; Lee S. Synthetic antimicrobial peptide tuning permits membrane disruption and interpeptide synergy. ACS Pharmacol. Transl. Sci. 2020, 3 (3), 418–424. 10.1021/acsptsci.0c00001. 32566907 PMC7296547

[ref76] Wimley W.; White S. Experimentally determined hydrophobicity scale for proteins at membrane interfaces. Nature Structural & Molecular Biology 1996, 3, 842–848. 10.1038/nsb1096-842. 8836100

[ref77] Clarke M.; Hind C. K.; Ferguson P. M.; Manzo G.; Mistry B.; Yue B.; Romanopulos J.; Clifford M.; Bui T. T.; Drake A. F.; Lorenz C. D.; Sutton J. M.; Mason A. J. Synergy between Winter Flounder Antimicrobial peptides. npj Antimicrobials & Resistance 2023, 1 (8), 1–16. 10.1038/s44259-023-00010-7. PMC11057203 38686212

[ref78] Ferguson P. M.; Clarke M.; Manzo G.; Hind C. K.; Clifford M.; Sutton J. M.; Lorenz C. D.; Phoenix D. A.; Mason A. J. Temporin B forms heterooligomers with Temporin L, modifies its membrane activity and increases the cooperativity of its antibacterial pharmacodynamic profile. Biochemistry 2022, 61, 1029–1040. 10.1021/acs.biochem.1c00762. 35609188 PMC9178791

[ref79] Manzo G.; Hind C. K.; Ferguson P. M.; Amison R. T.; Hodgson-Casson A. C.; Ciazynska K. A.; Weller B. J.; Clarke M.; Lam C.; Man R. C. H.; O’shaughnessy B. G.; Clifford M.; Bui T. T.; Drake A. F.; Atkinson R. A.; Lam J. K. W.; Pitchford S. C.; Page C. P.; Phoenix D. A.; Lorenz C. D.; Sutton J. M.; Mason A. J. A Pleurocidin analogue with greater conformational flexibility, enhanced antimicrobial potency and in vivo therapeutic efficacy. Commun. Biol. 2020, 3 (1), 697 10.1038/s42003-020-01420-3. 33247193 PMC7699649

[ref80] Manzo G.; Ferguson P. M.; Hind C.; Clifford M.; Gustilo V. B.; Ali H.; Bansal S. S.; Bui T. T.; Drake A. F.; Atkinson R. A.; Sutton J. M.; Lorenz C. D.; Phoenix D. A.; Mason A. J. Temporin L and aurein 2.5 have identical conformations but subtly distinct membrane and antibacterial activities. Sci. Rep. 2019, 9 (1), 10934 10.1038/s41598-019-47327-w. 31358802 PMC6662694

[ref81] Manzo G.; Ferguson P. M.; Gustilo V. B.; Hind C.; Clifford M.; Bui T. T.; Drake A. F.; Atkinson R. A.; Sutton M. J.; Batoni G.; Lorenz C. D.; Phoenix D. A.; Mason A. J. Minor sequence modifications in temporin B cause drastic changes in antibacterial potency and selectivity by fundamentally altering membrane activity. Sci. Rep. 2019, 9 (1385), 1–16. 10.1038/s41598-018-37630-3. 30718667 PMC6362004

