# Supplementary material for: QSAR Reveals Decreased Lipophilicity of Polar Residues Determines the Selectivity of Antimicrobial Peptide Activity
Source: ACS Omega. 2024 Jun 3;9(24):26030–49. doi: 10.1021/acsomega.4c01277 (PMC11191095; doi:10.1021/acsomega.4c01277)
Supplement: Supplementary file 1 — ao4c01277_si_001.pdf [file ao4c01277_si_001.pdf]

QSAR reveals decreased lipophilicity of polar residues determines the selectivity of antimicrobial peptide activity

Mandelie van der Walt[a], Dalton S. Moller[a], Rosalind J. van Wyk[a], Philip M. Ferguson[b], Charlotte K. Hind[c], Melanie Clifford[c], Phoebe Do Carmo Silva[c], J. Mark Sutton[c], A. James Mason[b]*, Megan J. Bester[d], Anabella R. M. Gaspar[a]*

[a] Department of Biochemistry, Genetics and Microbiology, Faculty of Natural and Agricultural Sciences, University of Pretoria, 0002, South Africa
[b] Institute of Pharmaceutical Science, School of Cancer & Pharmaceutical Science, King's College London, Franklin-Wilkins Building, 150 Stamford Street, London, SE1 9NH, United Kingdom
[c] Antimicrobial Discovery Development and Diagnostics, Vaccine Evaluation and Development Centre, UK Health Security Agency, SP4 0JG, United Kingdom
[d] Department of Anatomy, Faculty of Health Sciences, University of Pretoria, 0002, South Africa

***Corresponding authors:**

Prof. A.J. Mason, Institute of Pharmaceutical Science, King's College London, james.mason@kcl.ac.uk

Prof. A.R.M. Gaspar, Department of Biochemistry, Genetics and Microbiology, Faculty of Natural and Agricultural Sciences, University of Pretoria, anabella.gaspar@up.ac.za

SUPPLEMENTARY MATERIALS

1. Peptide properties per residue

Table S1: *Sidechain descriptors of amino acids used in PCA and QSAR analyses*

Amino acid	charge	L_P[a]	L_N[a]	L_P[b]	L_N[b]	$SCSA_P$ (Å) [c]	$SCSA_N$ (Å) [c]	MwP	MwN	CSL[d]	CAR [d]	HBacc	HBdon	R.B.i [e]
Ala	*0*	*0*	*0.31*	*0*	*-0.17*	*0*	*19.6*	*0*	*71.1*	*1*	*0*	*0*	*0*	*0*
Arg	*+1*	*-1.01*	*0*	*-0.81*	*0*	*39.7*	*0*	*156.2*	*0*	*0*	*0*	*1*	*4*	*4*
Asn	*0*	*-0.6*	*0*	*-0.42*	*0*	*114.5*	*0*	*114.1*	*0*	*0*	*0*	*1*	*2*	*2*
Asp	*-1*	*-2.57*	*0*	*-1.23*	*0*	*53.0*	*0*	*115.1*	*0*	*0*	*0*	*2*	*0*	*2*
Cys	*0*	*0*	*1.54*	*0*	*0.24*	*0*	*37.6*	*0*	*103.1*	*1*	*0*	*1*	*1*	*2*
Gln	*0*	*-0.22*	*0*	*-0.58*	*0*	*71.7*	*0*	*146.1*	*0*	*0*	*0*	*1*	*2*	*3*
Glu	*-1*	*-2.29*	*0*	*-2.02*	*0*	*61.6*	*0*	*129.1*	*0*	*0*	*0*	*2*	*0*	*3*
Gly	*0*	*0*	*0*	*0*	*-0.01*	*0*	*0*	*0*	*57.1*	*0*	*0*	*0*	*0*	*0*
His	*0*	*0.13*	*0*	*-0.17*	*0*	*75.7*	*0*	*137.1*	*0*	*0*	*0*	*1*	*1*	*2*
Ile	*0*	*0*	*1.8*	*0*	*0.31*	*0*	*68.4*	*0*	*113.2*	*1*	*0*	*0*	*0*	*2*
Leu	*0*	*0*	*1.7*	*0*	*0.56*	*0*	*69.9*	*0*	*113.2*	*1*	*0*	*0*	*0*	*2*
Lys	*+1*	*-0.99*	*0*	*-0.99*	*0*	*90.7*	*0*	*128.2*	*0*	*0*	*0*	*1*	*2*	*5*
Met	*0*	*0*	*1.23*	*0*	*0.23*	*0*	*78.2*	*0*	*131.2*	*1*	*0*	*1*	*0*	*3*
Phe	*0*	*0*	*1.79*	*0*	*1.13*	*0*	*92.2*	*0*	*147.2*	*0*	*1*	*0*	*0*	*2*
Pro	*0*	*0*	*0.72*	*0*	*-0.45*	*0*	*39.7*	*0*	*97.1*	*1*	*0*	*0*	*-1*	*0*
Ser	*0*	*-0.04*	*0*	*-0.13*	*0*	*26.7*	*0*	*87.1*	*0*	*0*	*0*	*1*	*1*	*2*
Thr	*0*	*0.26*	*0*	*-0.14*	*0*	*44.8*	*0*	*101.1*	*0*	*0*	*0*	*1*	*1*	*2*
Trp	*0*	*0*	*2.25*	*0*	*1.85*	*0*	*121.1*	*0*	*186.2*	*0*	*1*	*0*	*1*	*2*
Tyr	*0*	*0.96*	*0*	*0.94*	*0*	*102.0*	*0*	*163.2*	*0*	*0*	*1*	*1*	*1*	*3*
Val	*0*	*0*	*1.22*	*0*	*-0.07*	*0*	*51.1*	*0*	*99.1*	*1*	*0*	*0*	*0*	*1*

[a]The lipophilicity of polar or nonpolar amino acids at pH 7.0 taken from Frecer (28) and Fauchere & Pliska (54) calculated using $\pi_{FP} = \log P_{o/w}(\text{a.a.}) - \log P_{o/w}(\text{Gly})$
[b]The lipophilicity of polar or nonpolar amino acids at pH 7.0 according to the Wimley-white bilayer/water scale (74)
[c]The side chain surface areas (SCSA) of amino acids taken from Frecer (28) as calculated by Connolly surfaces method (46)
[d]Count of small lipophilic (CSL) or aromatic (CAR) residues taken from Frecer (28)
[e]The number of rotatable bonds in an amino acid side chain: R.B.i = Rotatable Bonds (a.a.) - Rotatable Bonds (Gly)

2. Antimicrobial activity

Table S2: *Antibiogram employed in the AMP susceptibility testing for susceptible and resistant (A) Gram-negative and -positive bacteria and (B) fungi.*

**A** Isolate	**Modal MICs (µg/mL)**				
	**Colistin**	**Tobra-mycin**	**Cipro-floxacin**	**Gentamycin**	**Ceftazidime**
Gram-negative					
E. coli NCTC 12923 (S)	0.0625	16	≤0.125	1	≤0.125
E. coli LEC001 (R)	2	8	32	4	128
P. aeruginosa PAO1 (S)	2	2	0.5	4	1
P. aeruginosa NCTC 13437 (R)	4	64	64	256	>128
A. baumannii ATCC 17978 (S)	1	4	0.5	0.5	<0.5
A. baumannii AYE (R)	1	64	128	>512	>128
K. pneumoniae M6 (S)	2	8	<0.5	0.25	<0.5
K. pneumoniae NCTC 13368 (R)	1	64	0.5	4	512
Gram-positive					
S. aureus (MSSA) ATCC 9144 (S)	ND	0.25-0.5	1	0.25	16
S. aureus (MRSA) NCTC 13616 (R)	>128	0.5	64	0.25	128
S. aureus (MRSA) USA300 (R)	ND	ND	64	4	32
S. aureus (MRSA) 1199B (R)	ND	0.25	8	0.25	8

**B** Isolate	**Modal MICs (µg/mL)**				
	**Fluconazole**	**Caspofungin**	**Voriconazole**	**Ampho-tericin B**	**Abafungin**
Fungi					
C. auris TDG 1912	>128	0.03	1	0.03	>128
C. albicans NCPF 3281	0.06-0.125	≤0.007	≤0.007	0.015	>128
C. glabrata NCPF 8018	4	≤0.007	0.125	0.03	>128
C. krusei NCPF 3876	8	≤0.007	0.06	0.06	0.25
C. tropicalis NCPF 8760	16	≤0.007	0.5	0.03	>128
C. parapsilosis NCPF 3209	0.25	0.125	≤0.007	≤0.007	0.25-0.5

Table S3: *Inhibitory activity of 15 frog derived AMPs against a panel of susceptible and resistant bacteria*

Isolate	Modal MICs (µg/mL)														
	X. laevis					*X. petersii*						*X. muelleri*			
	XPF	XPF10a	DRAMP 02272	DRAMP 02273	X1-20a	CPF-P2	CPF-P2a	CPF-P3	CPF-P3-10a	CPF-P3-12a	CPF-P3-20a	CPF-MW1	CPF-MW-10a	CPF-MW-12a	CPF-MW-20a
Gram-negative															
E. coli NCTC 12923 (S)	**4**	>128	**4**	>128	**8**	**8**	**32**	**8**	>128	>128	**128**	**8**	>128	>128	**16**
E. coli LEC001 (R)	**4**	>128	**8**	>128	>128	**16**	**64**	**8**	>128	>128	>128	**8**	>128	>128	**64**
P. aeruginosa PAO1 (S)	**128**	>128	>128	>128	>128	>128	>128	>128	>128	>128	>128	>128	>128	>128	>128
P. aeruginosa NCTC 13437 (R)	**128**	>128	>128	>128	>128	**128**	>128	**64**	>128	>128	>128	**128**	>128	>128	>128
A. baumannii ATCC 17978 (S)	**4**	>128	**4**	>128	**16**	**4**	**32**	**4**	>128	>128	**128**	**4**	>128	>128	**32**
A. baumannii AYE (R)	**4**	>128	**2**	>128	**16**	**8**	**64**	**8**	>128	>128	**128**	**4**	>128	>128	**32**
K. pneumoniae M6 (S)	**64**	>128	**32**	>128	**32**	**128**	>128	**32**	>128	>128	>128	**16**	>128	>128	>128
K. pneumoniae NCTC 13368 (R)	**32**	>128	**64**	>128	**64**	**128**	>128	**32**	>128	>128	>128	**32**	>128	>128	>128
Gram-positive															
S. aureus (MSSA) ATCC 9144 (S)	**32**	>128	**8**	>128	**16**	**4**	>128	**4**	>128	>128	>128	**4**	>128	>128	**64**
S. aureus (MRSA) NCTC 13616 (R)	**128**	>128	**16**	>128	>128	**4**	>128	**4**	>128	>128	>128	**4**	>128	>128	**128**
S. aureus (MRSA) USA300 (R)	**128**	>128	**32**	>128	>128	**8**	>128	**8**	>128	>128	>128	**8**	>128	>128	**128**
S. aureus (MRSA) 1199B (R)	**128**	>128	**64**	>128	>128	**8**	>128	**8**	>128	>128	>128	**4**	>128	>128	**128**

Table S3 B: *Inhibitory activity of 15 frog derived AMPs against a panel of susceptible and resistant bacteria* ***shown in µM***

Isolate	Modal MICs (µM)														
	X. laevis					***X. petersii***						***X. muelleri***			
	**XPF**	**XPF10a**	**DRAMP 02272**	**DRAMP 02273**	**X1-20a**	**CPF-P2**	**CPF-P2a**	**CPF-P3**	**CPF-P3-10a**	**CPF-P3-12a**	**CPF-P3-20a**	**CPF-MW1**	**CPF-MW-10a**	**CPF-MW-12a**	**CPF-MW-20a**
Gram-negative															
E. coli NCTC 12923 (S)	**1.5**	>120.6	**2**	>117.5	**3.4**	**2.9**	**18.6**	**2.9**	>122	>103.8	**65.7**	**2.7**	>110.8	>95.5	**6.5**
E. coli LEC001 (R)	**1.5**	>120.6	**4**	>117.5	>55	**5.8**	**37.1**	**2.9**	>122	>103.8	>65.7	**2.7**	>110.8	>95.5	**25.8**
P. aeruginosa PAO1 (S)	**46.5**	>120.6	>64	>117.5	>55	>46	>74	>47	>122	>103.8	>65.7	>43.1	>110.8	>95.5	>51.7
P. aeruginosa NCTC 13437 (R)	**46.5**	>120.6	>64	>117.5	>55	**46**	>74	**23.5**	>122	>103.8	>65.7	**43.1**	>110.8	>95.5	>51.7
A. baumannii ATCC 17978 (S)	**1.5**	>120.6	**2**	>117.5	**6.9**	**1.4**	**18.6**	**1.5**	>122	>103.8	**65.7**	**1.4**	>110.8	>95.5	**12.9**
A. baumannii AYE (R)	**1.5**	>120.6	**1**	>117.5	**6.9**	**2.9**	**37.1**	**2.9**	>122	>103.8	**65.7**	**1.4**	>110.8	>95.5	**12.9**
K. pneumoniae M6 (S)	**23.2**	>120.6	**16**	>117.5	**13.8**	**46**	>74	**11.8**	>122	>103.8	>65.7	**5.4**	>110.8	>95.5	>51.7
K. pneumoniae NCTC 13368 (R)	**11.6**	>120.6	**32**	>117.5	**27.5**	**46**	>74	**11.8**	>122	>103.8	>65.7	**10.8**	>110.8	>95.5	>51.7
Gram-positive															
S. aureus (MSSA) ATCC 9144 (S)	**11.6**	>120.6	**4**	>117.5	**6.9**	**1.4**	>74	**1.5**	>122	>103.8	>65.7	**1.4**	>110.8	>95.5	**25.8**
S. aureus (MRSA) NCTC 13616 (R)	**46.5**	>120.6	**8**	>117.5	>55	**1.4**	>74	**1.5**	>122	>103.8	>65.7	**1.4**	>110.8	>95.5	**51.7**
S. aureus (MRSA) USA300 (R)	**46.5**	>120.6	**16**	>117.5	>55	**2.9**	>74	**2.9**	>122	>103.8	>65.7	**2.7**	>110.8	>95.5	**51.7**
S. aureus (MRSA) 1199B (R)	**46.5**	>120.6	**32**	>117.5	>55	**2.9**	>74	**2.9**	>122	>103.8	>65.7	**1.4**	>110.8	>95.5	**51.7**

Table S4: *Inhibitory activity of 16 scorpion derived AMPs against a panel of susceptible and resistant bacteria*

Isolate	Modal MICs (µg/mL)															
	P. schlectheri				***O. carinatus***		***A. amoeruxi***							***O. madagascariensis***		
	**PS-PB-8a**	**PS-PB-14a**	**PS-PB-16a**	**PS-PB-20a**	**Opis16a**	**Opis8a**	**AamAP1**	**AamAP-S1**	**AamAP-S1a**	**AamAP1-Lys**	**AamAP1-Lysa**	**A3**	**A3a**	**K3-IsCTa**	**K7P8K11-IsCTa**	**A1F5K8-IsCTa**
Gram-negative																
E. coli NCTC 12923 (S)	>128	>128	**16**	**32**	**4**	>128	>128	**64**	**16**	**4**	**4**	>128	**16**	**8**	**16**	**8**
E. coli LEC001 (R)	>128	>128	**64**	**64**	**4**	>128	>128	**32**	**16**	**8**	**8**	>128	**32**	**8**	**64**	**8**
P. aeruginosa PAO1 (S)	>128	>128	**64**	**128**	**128**	>128	>128	>128	>128	**32**	**16**	>128	**32**	>128	>128	>128
P. aeruginosa NCTC 13437 (R)	>128	>128	**128**	>128	**32**	>128	>128	>128	**128**	**16**	**16**	>128	**16**	**64**	**64**	**64**
A. baumannii ATCC 17978 (S)	>128	>128	**16**	**8**	**2**	>128	**128**	**64**	**8**	**4**	**2**	**8**	**8**	**4**	**8**	**4**
A. baumannii AYE (R)	>128	>128	**32**	**32**	**2**	>128	**128**	**32**	**8**	**2**	**4**	**16**	**16**	**8**	**8**	**8**
K. pneumoniae M6 (S)	>128	>128	>128	>128	**8**	>128	>128	>128	**64**	**16**	**8**	>128	**32**	**16**	**32**	**16**
K. pneumoniae NCTC 13368 (R)	>128	>128	>128	>128	**16**	>128	>128	>128	**64**	**16**	**8**	>128	**32**	**16**	**64**	**16**
Gram-positive																
S. aureus (MSSA) ATCC 9144 (S)	>128	>128	**32**	**8**	**2**	>128	**64**	**16**	**4**	**4**	**4**	**16**	**16**	**8**	**8**	**4**
S. aureus (MRSA) NCTC 13616 (R)	>128	>128	>128	>128	**4**	>128	**64**	**64**	**8**	**4**	**2**	**16**	**16**	**8**	**64**	**16**
S. aureus (MRSA) USA300 (R)	>128	>128	>128	>128	**8**	>128	**128**	**64**	**8**	**8**	**4**	**32**	**16**	**16**	**64**	**16**
S. aureus (MRSA) 1199B (R)	>128	>128	>128	>128	**4**	>128	**64**	**32**	**4**	**4**	**4**	**16**	**8**	**8**	**32**	**8**

Table S4 B: *Inhibitory activity of 16 scorpion derived AMPs against a panel of susceptible and resistant bacteria* ***shown in µM***

Isolate	**Modal MICs (µM)**															
	P. schlectheri				***O. carinatus***		***A. amoeruxi***							***O. madagascariensis***		
	**PS-PB-8a**	**PS-PB-14a**	**PS-PB-16a**	**PS-PB-20a**	**Opis16a**	**Opis8a**	**AamAP1**	**AamAP-S1**	**AamAP-S1a**	**AamAP1-Lys**	**AamAP1-Lysa**	**A3**	**A3a**	**K3-IsCTa**	**K7P8 K11-IsCTa**	**A1F5K8-IsCTa**
Gram-negative																
E. coli NCTC 12923 (S)	>127.2	>63.8	**8.5**	**13.9**	**2**	>133.2	>64.8	**32.2**	**8.1**	**1.8**	**1.8**	>62.9	**7.9**	**5**	**9.9**	**5**
E. coli LEC001 (R)	>127.2	>63.8	**33.9**	**27.8**	**2**	>133.2	>64.8	**16.1**	**8.1**	**3.6**	**3.6**	>62.9	**15.7**	**5**	**39.5**	**5**
P. aeruginosa PAO1 (S)	>127.2	>63.8	**33.9**	**55.7**	**64.7**	>133.2	>64.8	>64.5	>64.8	**14.4**	**7.2**	>62.9	**15.7**	>79.5	>79	>79.9
P. aeruginosa NCTC 13437 (R)	>127.2	>63.8	**67.8**	>55.7	**16.2**	>133.2	>64.8	>64.5	**64.8**	**7.2**	**7.2**	>62.9	**7.9**	**39.8**	**39.5**	**39.9**
A. baumannii ATCC 17978 (S)	>127.2	>63.8	**8.5**	**3.5**	**1**	>133.2	**64.8**	**32.2**	**4.1**	**1.8**	**0.9**	**3.9**	**3.9**	**2.5**	**4.9**	**2.5**
A. baumannii AYE (R)	>127.2	>63.8	**17**	**13.9**	**1**	>133.2	**64.8**	**16.1**	**4.1**	**0.9**	**1.8**	**7.9**	**7.9**	**5**	**4.9**	**5**
K. pneumoniae M6 (S)	>127.2	>63.8	>67.8	>55.7	**4**	>133.2	>64.8	>64.5	**32.4**	**7.2**	**3.6**	>62.9	**15.7**	**9.9**	**19.8**	**10**
K. pneumoniae NCTC 13368 (R)	>127.2	>63.8	>67.8	>55.7	**8.1**	>133.2	>64.8	>64.5	**32.4**	**7.2**	**3.6**	>62.9	**15.7**	**9.9**	**39.5**	**10**
Gram-positive																
S. aureus (MSSA) ATCC 9144 (S)	>127.2	>63.8	**17**	**3.5**	**1**	>133.2	**32.4**	**8.1**	**2**	**1.8**	**1.8**	**7.9**	**7.9**	**5**	**4.9**	**2.5**
S. aureus (MRSA) NCTC 13616 (R)	>127.2	>63.8	>67.8	>55.7	**2**	>133.2	**32.4**	**32.2**	**4.1**	**1.8**	**0.9**	**7.9**	**7.9**	**5**	**39.5**	**10**
S. aureus (MRSA) USA300 (R)	>127.2	>63.8	>67.8	>55.7	**4**	>133.2	**64.8**	**32.2**	**4.1**	**3.6**	**1.8**	**15.7**	**7.9**	**9.9**	**39.5**	**10**
S. aureus (MRSA) 1199B (R)	>127.2	>63.8	>67.8	>55.7	**2**	>133.2	**32.4**	**16.1**	**2**	**1.8**	**1.8**	**7.9**	**3.9**	**5**	**19.8**	**5**

Table S5: *Inhibitory activity of 11 tick derived AMPs against a panel of susceptible and resistant bacteria*

**Isolate**	**Modal MICs (µg/mL)**										
	O. savignyi							***O. moubata***			
	**Os**	**Osa**	**Os-C**	**Os(3-12)a**	**Os(11-22)a**	**W3(Os-C)a**	**W(Os-C)a**	**OmDefB**	**OmDef19**	**W-OmC-Ca**	**W-OmB-Ca**
Gram-negative											
E. coli NCTC 12923 (S)	>128	>128	>128	>128	>128	**64**	>128	>128	>128	**128**	**128**
E. coli LEC001 (R)	>128	>128	>128	>128	>128	>128	>128	>128	>128	>128	>128
P. aeruginosa PAO1 (S)	>128	>128	>128	>128	>128	>128	>128	>128	>128	>128	>128
P. aeruginosa NCTC 13437 (R)	>128	>128	>128	>128	>128	>128	>128	>128	>128	>128	>128
A. baumannii ATCC 17978 (S)	>128	>128	>128	>128	>128	**32**	**64**	>128	>128	**128**	**64**
A. baumannii AYE (R)	>128	>128	>128	>128	>128	**16**	**32**	>128	>128	**128**	**32**
K. pneumoniae M6 (S)	>128	>128	>128	>128	>128	>128	>128	>128	>128	>128	>128
K. pneumoniae NCTC 13368 (R)	>128	>128	>128	>128	>128	>128	>128	>128	>128	>128	>128
Gram-positive											
S. aureus (MSSA) ATCC 9144 (S)	>128	>128	>128	>128	>128	**32**	**128**	>128	>128	**128**	**128**
S. aureus (MRSA) NCTC 13616 (R)	>128	>128	>128	>128	>128	>128	>128	>128	>128	>128	>128
S. aureus (MRSA) USA300 (R)	>128	>128	>128	>128	>128	>128	>128	>128	>128	>128	>128
S. aureus (MRSA) 1199B (R)	>128	>128	>128	>128	>128	>128	>128	>128	>128	>128	>128

Table S5 B: *Inhibitory activity of 11 tick derived AMPs against a panel of susceptible and resistant bacteria* ***shown in μM***

Isolate	Modal MICs (μM)										
	O. savignyi							*O. moubata*			
	Os	Osa	Os-C	Os(3-12)a	Os(11-22)a	W3(Os-C)a	W(Os-C)a	OmDefB	OmDef19	W-OmC-Ca	W-OmB-Ca
Gram-negative											
E. coli NCTC 12923 (S)	>51.7	>51.7	>59	>112	>91.7	**23.5**	>54.4	>49.9	>56.8	**54.3**	**52.5**
E. coli LEC001 (R)	>51.7	>51.7	>59	>112	>91.7	>46.9	>54.4	>49.9	>56.8	>54.3	>52.5
P. aeruginosa PAO1 (S)	>51.7	>51.7	>59	>112	>91.7	>46.9	>54.4	>49.9	>56.8	>54.3	>52.5
P. aeruginosa NCTC 13437 (R)	>51.7	>51.7	>59	>112	>91.7	>46.9	>54.4	>49.9	>56.8	>54.3	>52.5
A. baumannii ATCC 17978 (S)	>51.7	>51.7	>59	>112	>91.7	**11.7**	**27.2**	>49.9	>56.8	**54.3**	**26.2**
A. baumannii AYE (R)	>51.7	>51.7	>59	>112	>91.7	**5.9**	**13.6**	>49.9	>56.8	**54.3**	**13.1**
K. pneumoniae M6 (S)	>51.7	>51.7	>59	>112	>91.7	>46.9	>54.4	>49.9	>56.8	>54.3	>52.5
K. pneumoniae NCTC 13368 (R)	>51.7	>51.7	>59	>112	>91.7	>46.9	>54.4	>49.9	>56.8	>54.3	>52.5
Gram-positive											
S. aureus (MSSA) ATCC 9144 (S)	>51.7	>51.7	>59	>112	>91.7	**11.7**	**54.4**	>49.9	>56.8	**54.3**	**52.5**
S. aureus (MRSA) NCTC 13616 (R)	>51.7	>51.7	>59	>112	>91.7	>46.9	>54.4	>49.9	>56.8	>54.3	>52.5
S. aureus (MRSA) USA300 (R)	>51.7	>51.7	>59	>112	>91.7	>46.9	>54.4	>49.9	>56.8	>54.3	>52.5
S. aureus (MRSA) 1199B (R)	>51.7	>51.7	>59	>112	>91.7	>46.9	>54.4	>49.9	>56.8	>54.3	>52.5

Table S6: *Inhibitory activity of 4 primate derived AMPs against a panel of susceptible and resistant bacteria*

Isolate	Modal MICs (µg/mL)			
	Papio anubis			
	ThetaDefA	ThetaDefB	BTD15a	BTD11a
Gram-negative				
E. coli NCTC 12923 (S)	>128	>128	**8**	>128
E. coli LEC001 (R)	>128	>128	**16**	>128
P. aeruginosa PAO1 (S)	>128	>128	>128	>128
P. aeruginosa NCTC 13437 (R)	>128	>128	>128	>128
A. baumannii ATCC 17978 (S)	>128	>128	**4**	>128
A. baumannii AYE (R)	>128	>128	**8**	>128
K. pneumoniae M6 (S)	>128	>128	**32**	>128
K. pneumoniae NCTC 13368 (R)	>128	>128	**32**	>128
Gram-positive				
S. aureus (MSSA) ATCC 9144 (S)	>128	>128	**8**	>128
S. aureus (MRSA) NCTC 13616 (R)	>128	>128	**8**	>128
S. aureus (MRSA) USA300 (R)	>128	>128	**8**	>128
S. aureus (MRSA) 1199B (R)	>128	>128	**8**	>128

Table S6 B: *Inhibitory activity of 4 primate derived AMPs against a panel of susceptible and resistant bacteria* ***shown in µM***

Isolate	Modal MICs (µM)			
	Papio anubis			
	ThetaDefA	ThetaDefB	BTD15a	BTD11a
Gram-negative				
E. coli NCTC 12923 (S)	>122.6	>116.4	**4.4**	>94
E. coli LEC001 (R)	>122.6	>116.4	**8.8**	>94
P. aeruginosa PAO1 (S)	>122.6	>116.4	>70.2	>94
P. aeruginosa NCTC 13437 (R)	>122.6	>116.4	>70.2	>94
A. baumannii ATCC 17978 (S)	>122.6	>116.4	**2.2**	>94
A. baumannii AYE (R)	>122.6	>116.4	**4.4**	>94
K. pneumoniae M6 (S)	>122.6	>116.4	**17.5**	>94
K. pneumoniae NCTC 13368 (R)	>122.6	>116.4	**17.5**	>94
Gram-positive				
S. aureus (MSSA) ATCC 9144 (S)	>122.6	>116.4	**4.4**	>94
S. aureus (MRSA) NCTC 13616 (R)	>122.6	>116.4	**4.4**	>94
S. aureus (MRSA) USA300 (R)	>122.6	>116.4	**4.4**	>94
S. aureus (MRSA) 1199B (R)	>122.6	>116.4	**4.4**	>94

Table S7: *Inhibitory activity of 15 frog derived AMPs against a panel of fungal species*

**Isolate**		**Modal MICs (µg/mL)**														
		X. laevis					***X. petersii***						***X. muelleri***			
		**XPF**	**XPF10a**	**DRAMP 02272**	**DRAMP 02273**	**X1-20a**	**CPF-P2**	**CPF-P2a**	**CPF-P3**	**CPF-P3-10a**	**CPF-P3-12a**	**CPF-P3-20a**	**CPF-MW1**	**CPF-MW-10a**	**CPF-MW-12a**	**CPF-MW-20a**
C. auris TDG 1912	**MIC50**	>128	>128	>128	>128	>128	>128	>128	>128	>128	>128	>128	>128	>128	>128	>128
	**MIC90**	>128	>128	>128	>128	>128	>128	>128	>128	>128	>128	>128	>128	>128	>128	>128
C. albicans NCPF 3281	**MIC50**	>128	>128	>128	>128	>128	>128	>128	>128	>128	>128	>128	>128	>128	>128	>128
	**MIC90**	>128	>128	>128	>128	>128	>128	>128	>128	>128	>128	>128	>128	>128	>128	>128
C. glabrata NCPF 8018	**MIC50**	>128	>128	>128	>128	>128	>128	>128	>128	>128	>128	>128	>128	>128	>128	>128
	**MIC90**	>128	>128	>128	>128	>128	>128	>128	>128	>128	>128	>128	>128	>128	>128	>128
C. krusei NCPF 3876	**MIC50**	>128	>128	**128**	>128	**64**	**64**	>128	**64**	>128	>128	>128	**64**	>128	>128	>128
	**MIC90**	>128	>128	**128**	>128	**64**	**64**	>128	**64**	>128	>128	>128	**64**	>128	>128	>128
C. tropicalis NCPF 8760	**MIC50**	**32**	>128	**32**	>128	**16**	**16**	>128	**8**	>128	>128	>128	**32**	>128	>128	>128
	**MIC90**	**32**	>128	**32**	>128	**32**	**16**	>128	**16**	>128	>128	>128	**32**	>128	>128	>128
C. parapsilosis NCPF 3209	**MIC50**	>128	>128	>128	>128	>128	>128	>128	>128	>128	>128	>128	>128	>128	>128	>128
	**MIC90**	>128	>128	>128	>128	>128	>128	>128	>128	>128	>128	>128	>128	>128	>128	>128

Table S7 B: *Inhibitory activity of 15 frog derived AMPs against a panel of fungal species* ***shown in µM***

Isolate		Modal MICs (µM)														
		X. laevis					*X. petersii*						*X. muelleri*			
		XPF	XPF10a	DRAMP 02272	DRAMP 02273	X1-20a	CPF-P2	CPF-P2a	CPF-P3	CPF-P3-10a	CPF-P3-12a	CPF-P3-20a	CPF-MW1	CPF-MW-10a	CPF-MW-12a	CPF-MW-20a
C. auris TDG 1912	**MIC50**	>46.5	>120.6	>64	>117.5	>55	>46	>74	>47	>122	>103.8	>65.7	>43.1	>110.8	>95.5	>51.7
	**MIC90**	>46.5	>120.6	>64	>117.5	>55	>46	>74	>47	>122	>103.8	>65.7	>43.1	>110.8	>95.5	>51.7
C. albicans NCPF 3281	**MIC50**	>46.5	>120.6	>64	>117.5	>55	>46	>74	>47	>122	>103.8	>65.7	>43.1	>110.8	>95.5	>51.7
	**MIC90**	>46.5	>120.6	>64	>117.5	>55	>46	>74	>47	>122	>103.8	>65.7	>43.1	>110.8	>95.5	>51.7
C. glabrata NCPF 8018	**MIC50**	>46.5	>120.6	>64	>117.5	>55	>46	>74	>47	>122	>103.8	>65.7	>43.1	>110.8	>95.5	>51.7
	**MIC90**	>46.5	>120.6	>64	>117.5	>55	>46	>74	>47	>122	>103.8	>65.7	>43.1	>110.8	>95.5	>51.7
C. krusei NCPF 3876	**MIC50**	>46.5	>120.6	**64**	>117.5	**27.5**	**23**	>74	**23.5**	>122	>103.8	>65.7	**10.8**	>110.8	>95.5	>51.7
	**MIC90**	>46.5	>120.6	**64**	>117.5	**27.5**	**23**	>74	**23.5**	>122	>103.8	>65.7	**10.8**	>110.8	>95.5	>51.7
C. tropicalis NCPF 8760	**MIC50**	**11.6**	>120.6	**16**	>117.5	**6.9**	**5.8**	>74	**2.9**	>122	>103.8	>65.7	**21.6**	>110.8	>95.5	>51.7
	**MIC90**	**11.6**	>120.6	**16**	>117.5	**13.8**	**5.8**	>74	**5.8**	>122	>103.8	>65.7	**21.6**	>110.8	>95.5	>51.7
C. parapsilosis NCPF 3209	**MIC50**	>46.5	>120.6	>64	>117.5	>55	>46	>74	>47	>122	>103.8	>65.7	>43.1	>110.8	>95.5	>51.7
	**MIC90**	>46.5	>120.6	>64	>117.5	>55	>46	>74	>47	>122	>103.8	>65.7	>43.1	>110.8	>95.5	>51.7

Table S8: *Inhibitory activity of 16 scorpion derived AMPs against a panel of fungal species*

Isolate		Modal MICs (µg/mL)															
		P. schlectheri				*O. carinatus*		*A. amoeruxi*							*O. madagascariensis*		
		PS-PB-8a	PS-PB-14a	PS-PB-16a	PS-PB-20a	Opis-16a	Opis8a	AamAP1	AamAP-S1	AamAP-S1a	AamAP1-Lys	AamAP1-Lysa	A3	A3a	K3-IsCTa	K7P8K11-IsCTa	A1F5K8-IsCTa
C. auris TDG 1912	MIC50	>128	>128	>128	>128	>128	>128	>128	>128	>128	>128	>128	>128	>128	>128	>128	>128
	MIC90	>128	>128	>128	>128	>128	>128	>128	>128	>128	>128	>128	>128	>128	>128	>128	>128
C. albicans NCPF 3281	MIC50	>128	>128	>128	>128	**64**	>128	>128	>128	**128**	**128**	**32**	>128	>128	**32**	>128	**64**
	MIC90	>128	>128	>128	>128	**128**	>128	>128	>128	**128**	>128	**64**	>128	>128	**64**	>128	**64**
C. glabrata NCPF 8018	MIC50	>128	>128	>128	>128	**128**	>128	>128	>128	>128	**128**	**128**	>128	**128**	**64**	>128	**64**
	MIC90	>128	>128	>128	>128	**128**	>128	>128	>128	>128	>128	**128**	>128	**128**	**64**	>128	**128**
C. krusei NCPF 3876	MIC50	>128	>128	>128	**128**	**32**	>128	**128**	**64**	**64**	**16**	**16**	**32**	**32**	**16**	**32**	**32**
	MIC90	>128	>128	>128	>128	**32**	>128	**128**	**128**	**64**	**32**	**16**	**64**	**32**	**32**	**64**	**32**
C. tropicalis NCPF 8760	MIC50	>128	**64**	>128	**64**	**8**	>128	**64**	**32**	**16**	**4**	**2**	**8**	**64**	**4**	**8**	**8**
	MIC90	>128	**64**	>128	**64**	**8**	>128	**64**	**32**	**16**	**8**	**2**	**16**	**64**	**8**	**16**	**8**
C. parapsilosis NCPF 3209	MIC50	>128	>128	>128	>128	**64**	>128	>128	>128	**128**	**64**	**32**	>128	>128	**64**	**64**	**64**
	MIC90	>128	>128	>128	>128	**128**	>128	>128	>128	**128**	**64**	**32**	>128	>128	**64**	**128**	**64**

Table S8 B: *Inhibitory activity of 16 scorpion derived AMPs against a panel of fungal species* **shown in µM**

Isolate		Modal MICs (µM)															
		P. schlectheri				***O. carinatus***		***A. amoeruxi***							***O. madagascariensis***		
		**PS-PB-8a**	**PS-PB-14a**	**PS-PB-16a**	**PS-PB-20a**	**Opis-16a**	**Opis8a**	**AamAP1**	**AamAP-S1**	**AamAP-S1a**	**AamAP1-Lys**	**AamAP1-Lysa**	**A3**	**A3a**	**K3-IsCTa**	**K7P8K11-IsCTa**	**A1F5K8-IsCTa**
C. auris TDG 1912	**MIC50**	>127.2	>63.8	>67.8	>55.7	>64.7	>133.2	>64.8	>64.5	>64.8	>57.8	>57.8	>62.9	>62.9	>79.5	>79	>79.9
	**MIC90**	>127.2	>63.8	>67.8	>55.7	>64.7	>133.2	>64.8	>64.5	>64.8	>57.8	>57.8	>62.9	>62.9	>79.5	>79	>79.9
C. albicans NCPF 3281	**MIC50**	>127.2	>63.8	>67.8	>55.7	**32.4**	>133.2	>64.8	>64.5	**64.8**	**57.8**	**14.4**	>62.9	>62.9	**19.9**	>79	**39.9**
	**MIC90**	>127.2	>63.8	>67.8	>55.7	**64.7**	>133.2	>64.8	>64.5	**64.8**	>57.8	**28.8**	>62.9	>62.9	**39.8**	>79	**39.9**
C. glabrata NCPF 8018	**MIC50**	>127.2	>63.8	>67.8	>55.7	**64.7**	>133.2	>64.8	>64.5	>64.8	**57.8**	**57.8**	>62.9	**62.9**	**39.8**	>79	**39.9**
	**MIC90**	>127.2	>63.8	>67.8	>55.7	**64.7**	>133.2	>64.8	>64.5	>64.8	>57.8	**57.8**	>62.9	**62.9**	**39.8**	>79	**79.9**
C. krusei NCPF 3876	**MIC50**	>127.2	>63.8	>67.8	**55.7**	**16.2**	>133.2	**64.8**	**32.2**	**32.4**	**7.2**	**7.2**	**15.7**	**15.7**	**9.9**	**19.8**	**19.9**
	**MIC90**	>127.2	>63.8	>67.8	>55.7	**16.2**	>133.2	**64.8**	**64.5**	**32.4**	**14.4**	**7.2**	**31.4**	**15.7**	**19.9**	**39.5**	**19.9**
C. tropicalis NCPF 8760	**MIC50**	>127.2	**31.9**	>67.8	**27.8**	**4**	>133.2	**32.4**	**16.1**	**8.1**	**1.8**	**0.9**	**3.9**	**31.4**	**2.5**	**4.9**	**5**
	**MIC90**	>127.2	**31.9**	>67.8	**27.8**	**4**	>133.2	**32.4**	**16.1**	**8.1**	**3.6**	**0.9**	**7.9**	**31.4**	**5**	**9.9**	**5**
C. parapsilosis NCPF 3209	**MIC50**	>127.2	>63.8	>67.8	>55.7	**32.4**	>133.2	>64.8	>64.5	**64.8**	**28.8**	**14.4**	>62.9	>62.9	**39.8**	**39.5**	**39.8**
	**MIC90**	>127.2	>63.8	>67.8	>55.7	**64.7**	>133.2	>64.8	>64.5	**64.8**	**28.8**	**14.4**	>62.9	>62.9	**39.8**	**79**	**39.8**

Table S9: *Inhibitory activity of 11 tick derived AMPs against a panel of fungal species*

Isolate		Modal MICs (µg/mL)										
		O. savignyi							*O. moubata*			
		**Os**	**Osa**	**Os-C**	**Os(3-12)a**	**Os(11-22)a**	**W3(Os-C)a**	**W(Os-C)a**	**OmDefB**	**OmDef19**	**W-OmC-Ca**	**W-OmB-Ca**
C. auris TDG 1912	**MIC50**	>128	>128	>128	>128	>128	>128	>128	>128	>128	>128	>128
	**MIC90**	>128	>128	>128	>128	>128	>128	>128	>128	>128	>128	>128
C. albicans NCPF 3281	**MIC50**	>128	>128	>128	>128	>128	>128	>128	>128	>128	>128	>128
	**MIC90**	>128	>128	>128	>128	>128	>128	>128	>128	>128	>128	>128
C. glabrata NCPF 8018	**MIC50**	>128	>128	>128	>128	>128	>128	>128	>128	>128	>128	>128
	**MIC90**	>128	>128	>128	>128	>128	>128	>128	>128	>128	>128	>128
C. krusei NCPF 3876	**MIC50**	>128	>128	>128	>128	>128	>128	>128	>128	>128	>128	>128
	**MIC90**	>128	>128	>128	>128	>128	>128	>128	>128	>128	>128	>128
C. tropicalis NCPF 8760	**MIC50**	>128	>128	>128	>128	>128	>128	>128	>128	>128	>128	>128
	**MIC90**	>128	>128	>128	>128	>128	>128	>128	>128	>128	>128	>128
C. parapsilosis NCPF 3209	**MIC50**	>128	>128	>128	>128	>128	>128	>128	>128	>128	>128	>128
	**MIC90**	>128	>128	>128	>128	>128	>128	>128	>128	>128	>128	>128

Table S9 B: *Inhibitory activity of 11 tick derived AMPs against a panel of fungal species* ***shown in µM***

**Isolate**		**Modal MICs (µM)**										
		O. savignyi							***O. moubata***			
		**Os**	**Osa**	**Os-C**	**Os(3-12)a**	**Os(11-22)a**	**W3(Os-C)a**	**W(Os-C)a**	**OmDefB**	**OmDef19**	**W-OmC-Ca**	**W-OmB-Ca**
C. auris TDG 1912	**MIC50**	>51.7	>51.7	>59	>112	>91.7	>46.9	>54.4	>49.9	>56.8	>54.3	>52.5
	**MIC90**	>51.7	>51.7	>59	>112	>91.7	>46.9	>54.4	>49.9	>56.8	>54.3	>52.5
C. albicans NCPF 3281	**MIC50**	>51.7	>51.7	>59	>112	>91.7	>46.9	>54.4	>49.9	>56.8	>54.3	>52.5
	**MIC90**	>51.7	>51.7	>59	>112	>91.7	>46.9	>54.4	>49.9	>56.8	>54.3	>52.5
C. glabrata NCPF 8018	**MIC50**	>51.7	>51.7	>59	>112	>91.7	>46.9	>54.4	>49.9	>56.8	>54.3	>52.5
	**MIC90**	>51.7	>51.7	>59	>112	>91.7	>46.9	>54.4	>49.9	>56.8	>54.3	>52.5
C. krusei NCPF 3876	**MIC50**	>51.7	>51.7	>59	>112	>91.7	>46.9	>54.4	>49.9	>56.8	>54.3	>52.5
	**MIC90**	>51.7	>51.7	>59	>112	>91.7	>46.9	>54.4	>49.9	>56.8	>54.3	>52.5
C. tropicalis NCPF 8760	**MIC50**	>51.7	>51.7	>59	>112	>91.7	>46.9	>54.4	>49.9	>56.8	>54.3	>52.5
	**MIC90**	>51.7	>51.7	>59	>112	>91.7	>46.9	>54.4	>49.9	>56.8	>54.3	>52.5
C. parapsilosis NCPF 3209	**MIC50**	>51.7	>51.7	>59	>112	>91.7	>46.9	>54.4	>49.9	>56.8	>54.3	>52.5
	**MIC90**	>51.7	>51.7	>59	>112	>91.7	>46.9	>54.4	>49.9	>56.8	>54.3	>52.5

Table S10: *Inhibitory activity of 4 primate derived AMPs against a panel of fungal species*

Isolate		Modal MICs (µg/mL)			
		Papio anubis			
		**ThetaDefA**	**ThetaDefB**	**BTD15a**	**BTD11a**
C. auris TDG 1912	**MIC50**	>128	>128	>128	**64**
	**MIC90**	>128	>128	>128	>128
C. albicans NCPF 3281	**MIC50**	>128	>128	**32**	**32**
	**MIC90**	>128	>128	**64**	**64**
C. glabrata NCPF 8018	**MIC50**	>128	>128	**128**	**16**
	**MIC90**	>128	>128	**128**	**32**
C. krusei NCPF 3876	**MIC50**	>128	>128	**16**	**32**
	**MIC90**	>128	>128	**16**	**32**
C. tropicalis NCPF 8760	**MIC50**	>128	>128	**8**	**1**
	**MIC90**	>128	>128	**8**	**2**
C. parapsilosis NCPF 3209	**MIC50**	>128	>128	**128**	**4**
	**MIC90**	>128	>128	**128**	**16**

Table S10 B: *Inhibitory activity of 4 primate derived AMPs against a panel of fungal species* ***shown in µM***

Isolate		Modal MICs (µM)			
		Papio anubis			
		**ThetaDefA**	**ThetaDefB**	**BTD15a**	**BTD11a**
C. auris TDG 1912	**MIC50**	>122.6	>116.4	>70.2	**47**
	**MIC90**	>122.6	>116.4	>70.2	>94
C. albicans NCPF 3281	**MIC50**	>122.6	>116.4	**17.5**	**23.5**
	**MIC90**	>122.6	>116.4	**35**	**47**
C. glabrata NCPF 8018	**MIC50**	>122.6	>116.4	**70.2**	**11.8**
	**MIC90**	>122.6	>116.4	**70.2**	**23.5**
C. krusei NCPF 3876	**MIC50**	>122.6	>116.4	**8.8**	**23.5**
	**MIC90**	>122.6	>116.4	**8.8**	**23.5**
C. tropicalis NCPF 8760	**MIC50**	>122.6	>116.4	**4.4**	**0.7**
	**MIC90**	>122.6	>116.4	**4.4**	**1.5**
C. parapsilosis NCPF 3209	**MIC50**	>122.6	>116.4	**70.2**	**2.9**
	**MIC90**	>122.6	>116.4	**70.2**	**11.8**

3. Haemolytic activity

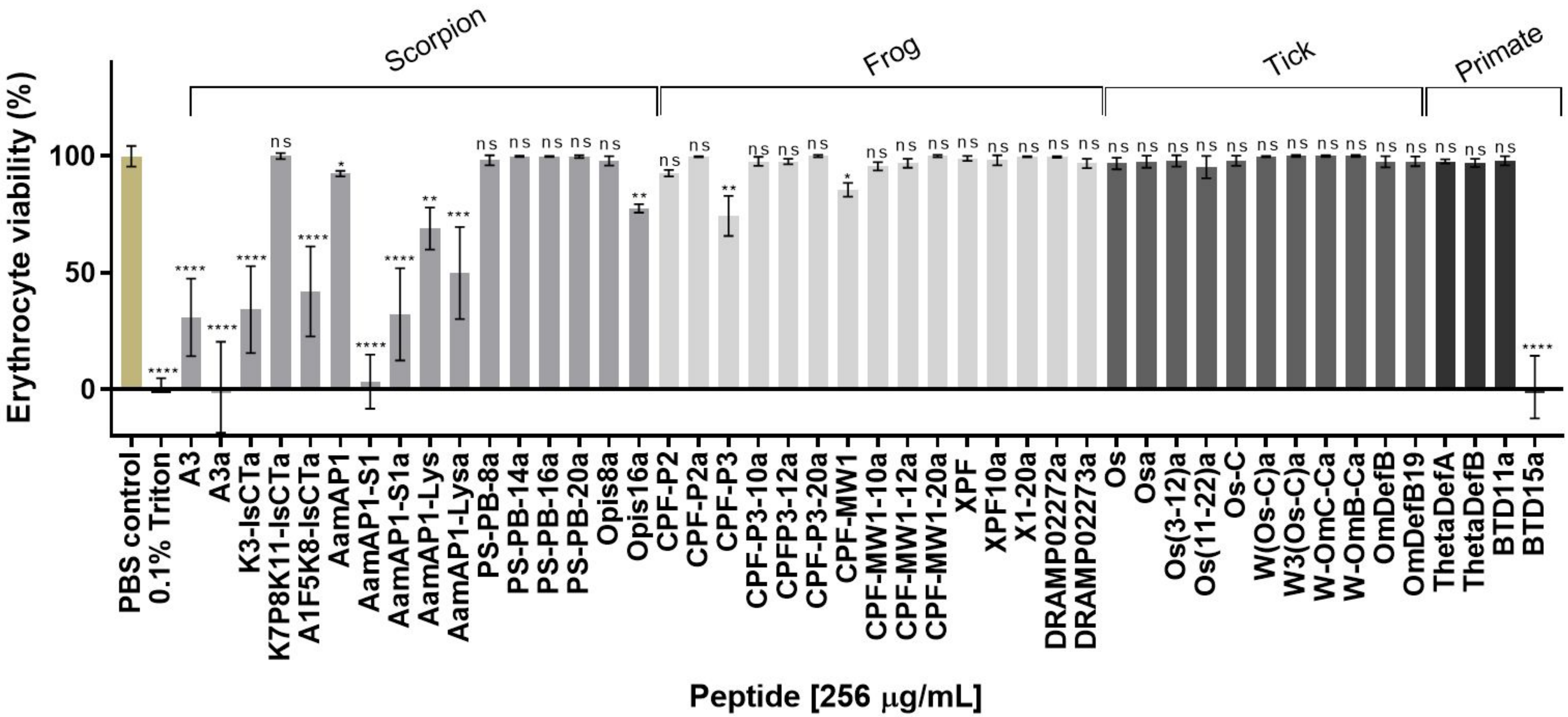

Figure S1: The percentage human erythrocyte viability after respective treatment with 46 novel AMPs. *Erythrocytes were treated with 16 scorpion, 15 frog, 11 tick and 4 primate derived AMPs at a screening concentration of 256 µg/mL each at 37 °C for 24h. Percentage viability is relative to the growth control; PBS treated cells. Error bars show the mean ± SE. Significant difference indicated by *, **, *** and **** represents p < 0.05, p < 0.01, p <0.001 and p < 0.0001 values. Results are of three independent biological repeats each performed in triplicate*

Table S11: *Haemolytic AMPs with an HC10 or HC50 below 256 µg/mL*

Peptide	Haemolysis	
	**HC10 µg/mL (µM)**	**HC50 µg/mL (µM)**
Frog derived AMPs		
CPF-P3*	**102 (37.5)**	>256 (>94)
CPF-MW1*	**214 (72)**	>256 (>86)
Scorpion derived AMPs		
Opis16a*	**155 (78.4)**	>256 (>129)
AamAP-S1*	**128 (64.5)**	**195 (98.2)**
AamAP-S1a*	**45 (22.8)**	**112 (56.7)**
AamAP1-Lys*	**94 (42.4)**	>256 (>115.5)
AamAP1-Lysa*	**53 (23.9)**	**256 (115.5)**
A3*	**9 (4.4)**	**54 (26.5)**
A3a*	**24 (11.8)**	**78 (38.3)**
K3-IsCTa*	**84 (52.2)**	**211 (131)**
A1F5K8-IsCTa*	**102 (63.6)**	**234 (146)**
Tick derived AMPs		
Primate derived AMPs		
BTD15a*	**9 (4.9)**	**33 (18.1)**

**Most active antimicrobial peptides identified in the screens*
***AMPs with an HC10 or HC50 above 256 µg/mL are regarded as non-haemolytic and the data not shown*

4. Cytotoxicity:

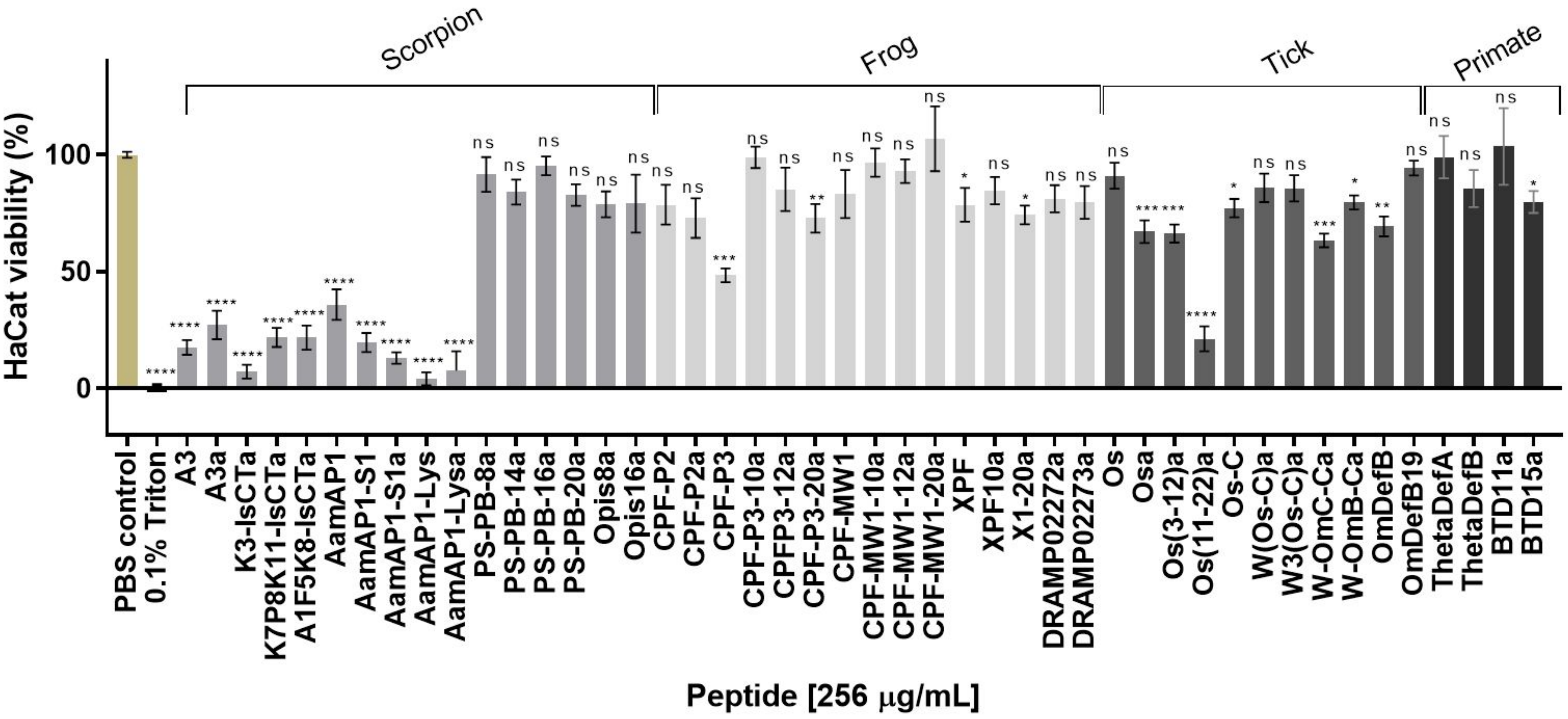

Figure S2: The percentage HaCat cell viability after respective treatment with 46 novel AMPs. *Hacat cells were treated with 16 scorpion, 15 frog, 11 tick and 4 primate derived AMPs at a screening concentration of 256 μg/mL each at 37 °C for 24h. Percentage viability is relative to the growth control; PBS treated HaCat cells. Error bars show the mean ± SE. Significant difference indicated by *, **, *** and **** represents p < 0.05, p < 0.01, p <0.001 and p < 0.0001 values. Results are of three independent biological repeats each performed in triplicate*

Table S12: *LC_{50} determined in the HaCat cell line of the 12 most toxic AMPs.*

Peptide		% HaCat cell death at initial screening (256µg/mL) ± SD	HaCat LC_{50} (µg/mL) (µM)
Scorpion AMPs			
1	A3 *	*100 ± 9.7*	*113.8* (55.9)
2	A3a *	*100 ± 10.5*	*279.7* (137.5)
3	K3-IsCTa *	*104.1 ± 11.9*	*69.4* (43.1)
4	K7P8K11-IsCTa	*89.2 ± 9.1*	*142.5* (88)
5	A1F5K8-IsCTa	*83.6 ±9.2*	*168.5* (105.1)
6	AamAP1	*44.3 ±20.2*	*270.2* (136.8)
7	AamAP1-S1	*85 ±17.9*	*121* (60.9)
8	AamAP1-S1a *	*97.6 ±12.6*	*59.3* (30)
9	AamAP1-Lys *	*99.1 ± 8.7*	*124.4* (56.1)
10	AamAP1-Lysa *	*94.8 ± 8.2*	*101.4* (45.8)
Frog AMPs			
11	CPF-P3	*39.9 ±12.3*	*283.5* (104.1)
Tick AMPs			
12	Os(11-22)a	*59.5 ±13.9*	*163.2* (116.8)

* indicates peptides with the highest toxicity

5. Circular dichroism spectra

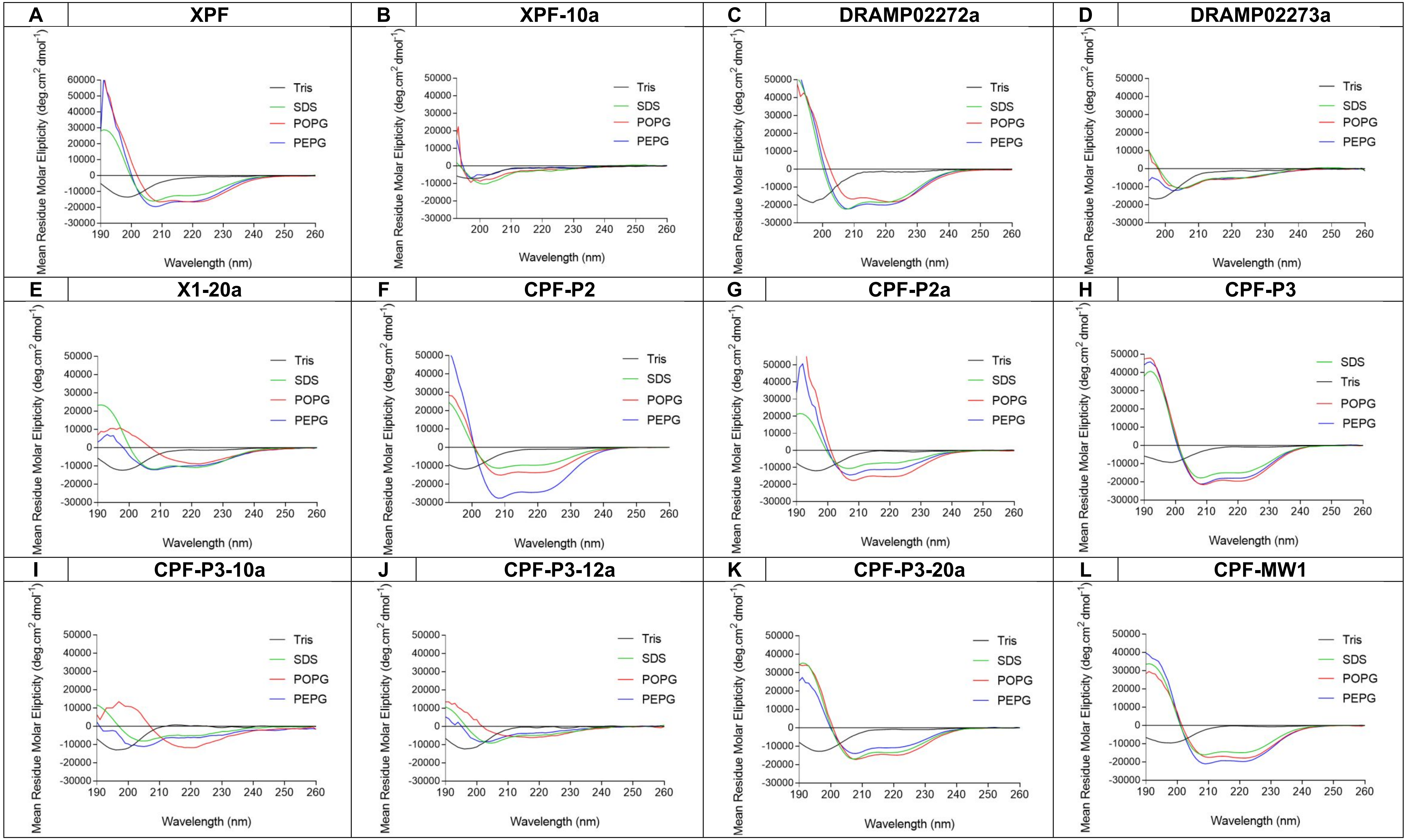

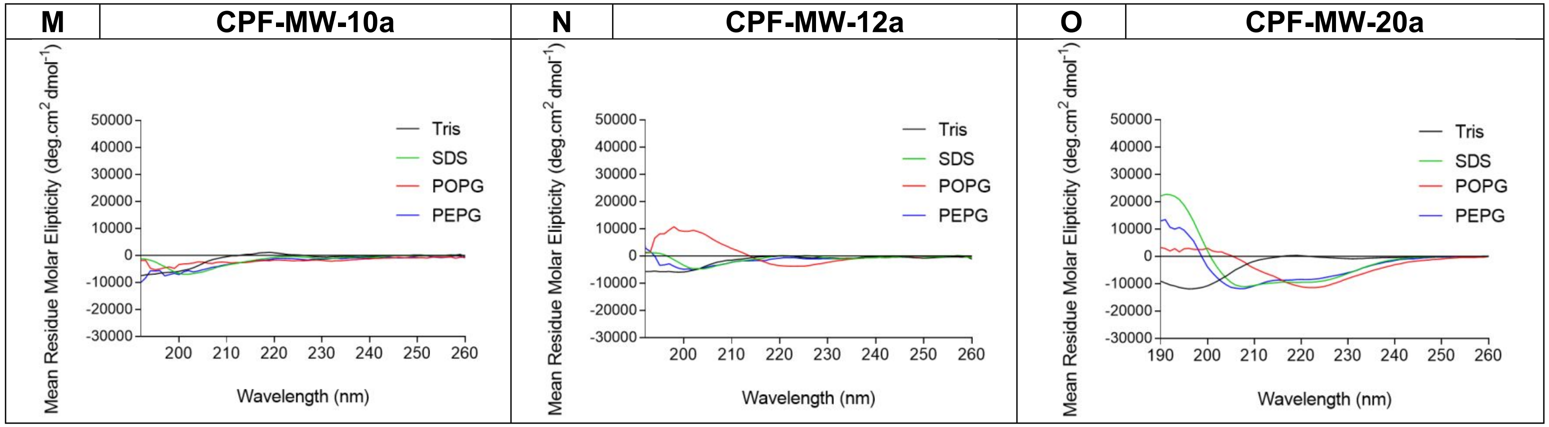

Figure S3: *CD spectra of the 15 frog derived AMPs in Tris (5mM), SDS (50mM), POPG (5mM) and PEPG (5mM) liposomes at pH of 7.4 and 23°C.*

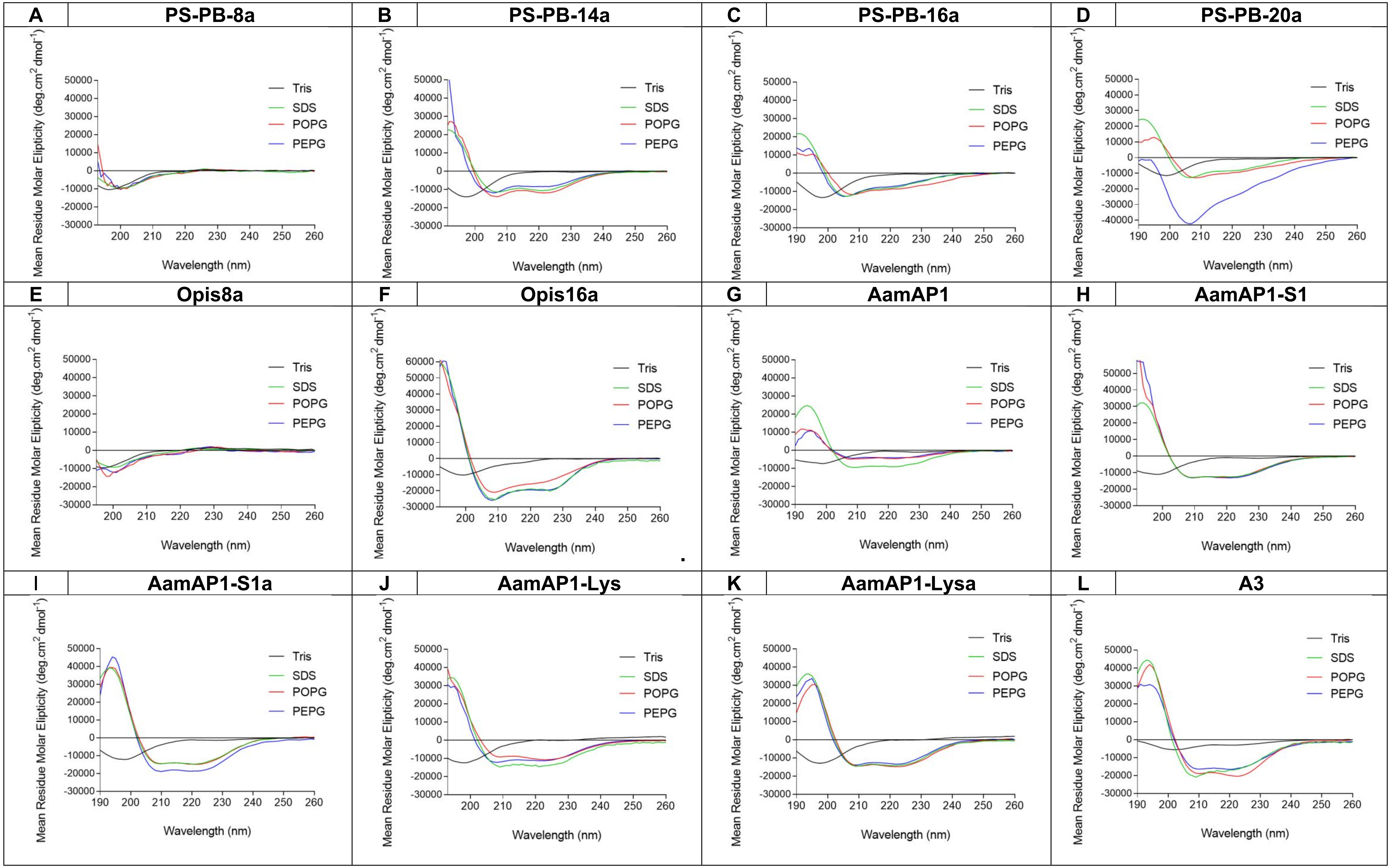
A
PS-PB-8a
B
PS-PB-14a
C
PS-PB-16a
D
PS-PB-20a
E
Opis8a
F
Opis16a
G
AamAP1
H
AamAP1-S1
I
AamAP1-S1a
J
AamAP1-Lys
K
AamAP1-Lysa
L
A3
Mean Residue Molar Elipticity (deg.cm2 dmol-1)
Wavelength (nm)
Tris
SDS
POPG
PEPG

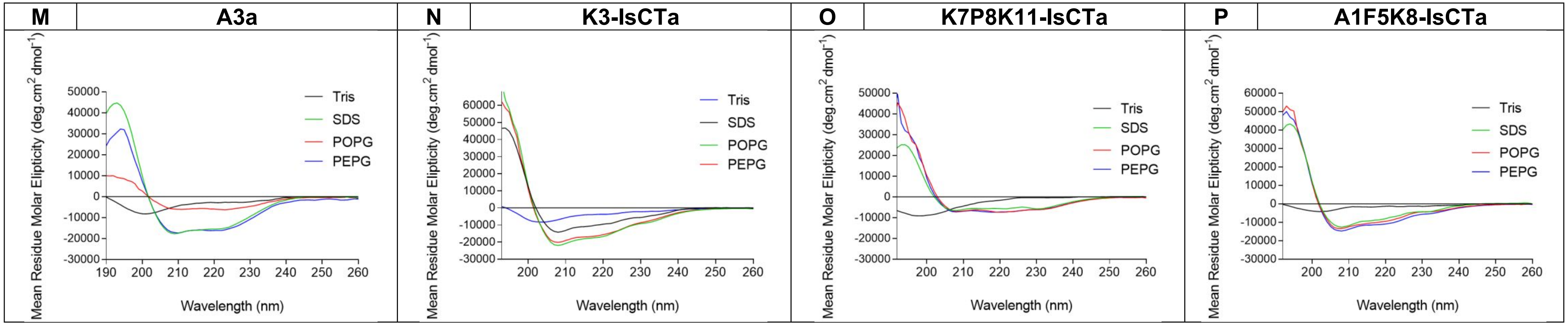

Figure S4: *CD spectra of the 16 scorpion derived AMPs in Tris (5mM), SDS (50mM), POPG (5mM) and PEPG (5mM) liposomes at pH of 7.4 and 23°C.*

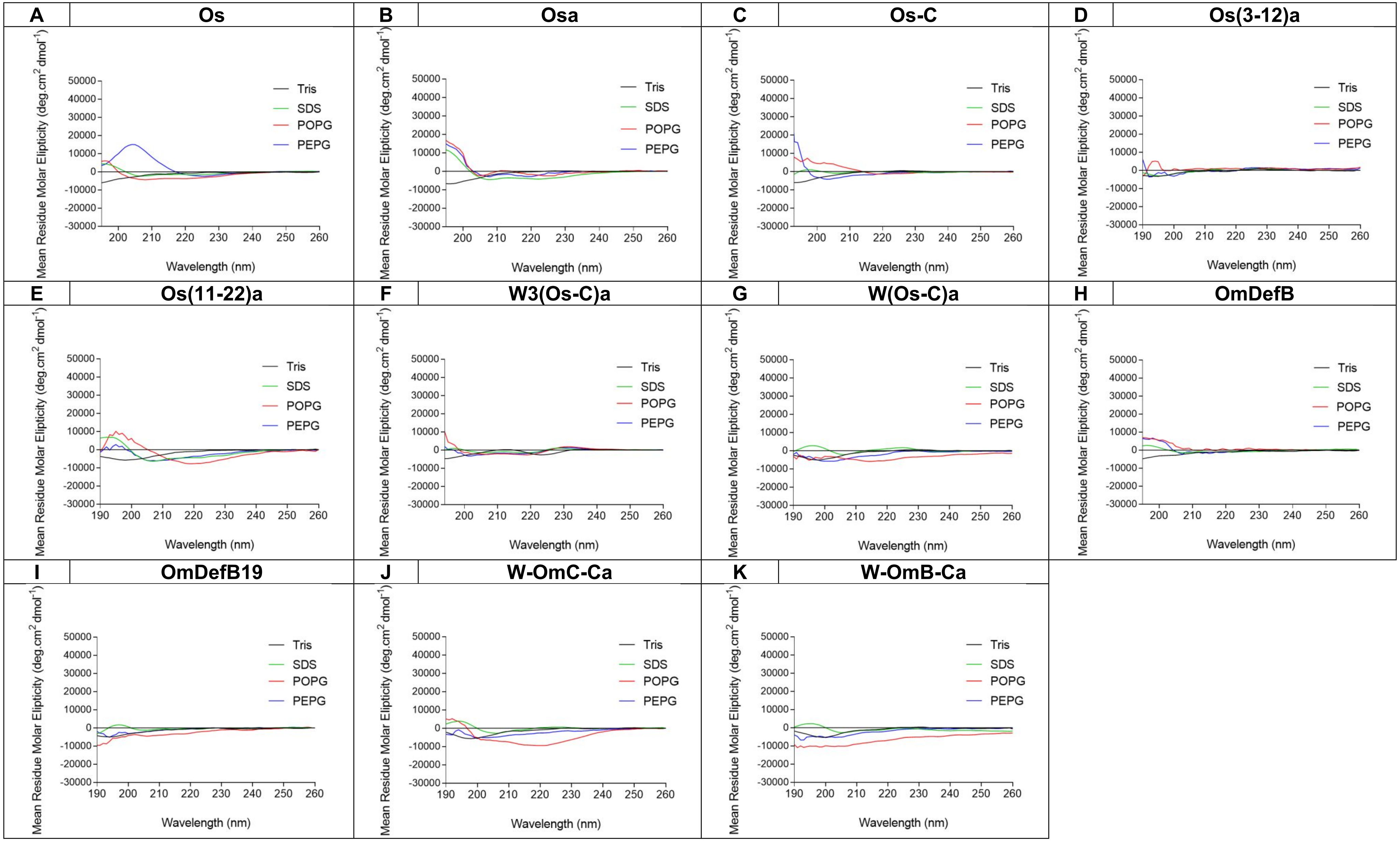

Figure S5: *CD spectra of the 11 tick derived AMPs in Tris (5mM), SDS (50mM), POPG (5mM) and PEPG (5mM) liposomes at pH of 7.4 and 23°C*

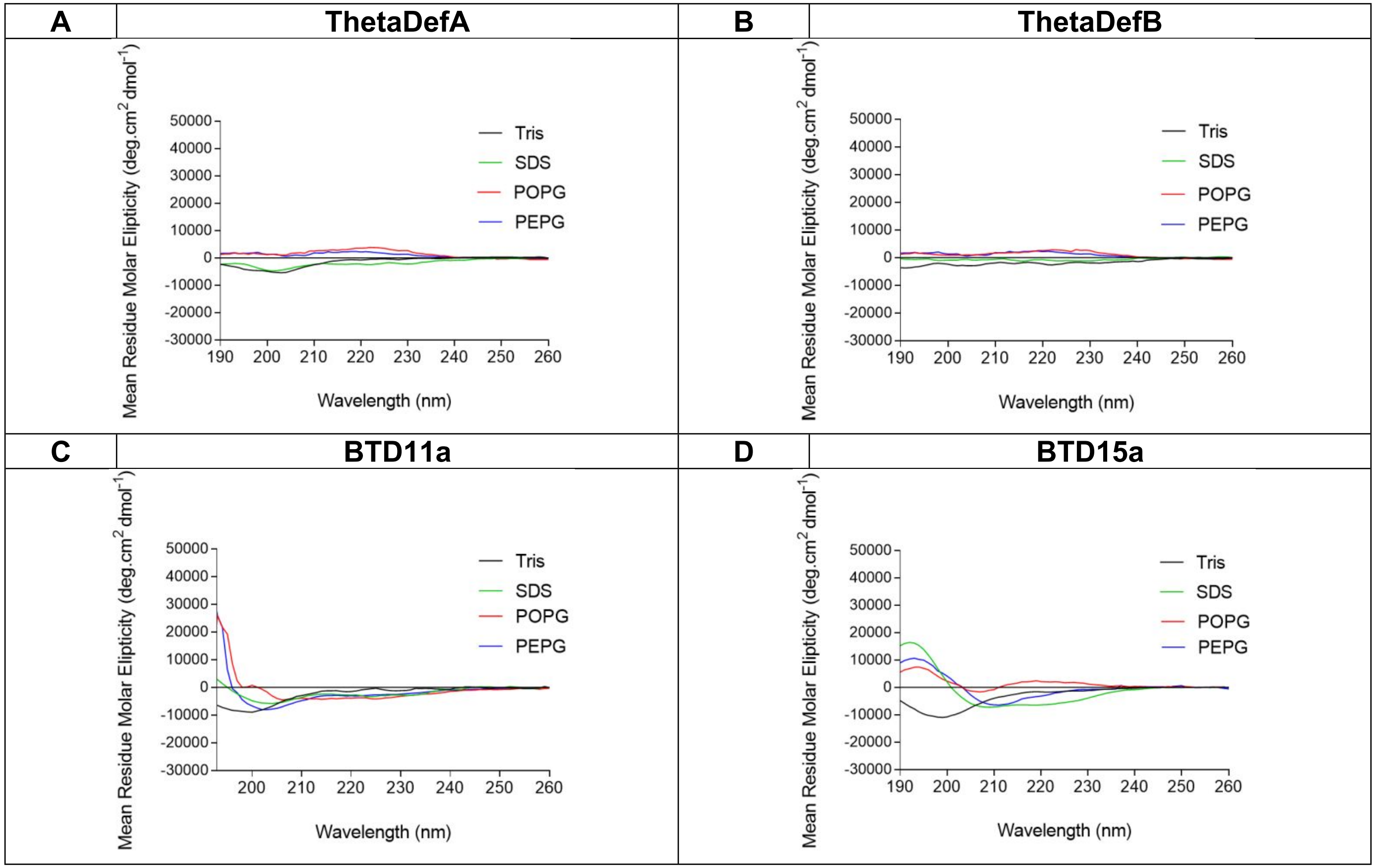

Figure S6: *CD spectra of the 4 primate derived AMPs in Tris (5mM), SDS (50mM), POPG (5mM) and PEPG (5mM) liposomes at pH of 7.4 and 23°C*

6. PCA

Bartlett's test of PCA appropriateness

Table S13: *Results of Bartlett's test calculated for all 46 peptides against Gram-positive and -negative bacteria*

	Gram-negative bacteria	Gram-positive bacteria	Fungi
Approximate χ^2			
PC-1	2016.25	2004.10	2037.89
PC-2	1643.94	1639.63	1671.67
Degrees of freedom			
PC-1	373.77	373.72	373.59
PC-2	365.94	365.61	365.56
Significance	<0.0001	<0.0001	<0.0001

Number of PCs selected per group for data interpretation based on a cumulative variance above 80%

Table S14: *Parameters determining the no. of PCs selected for Gram-negative, -positive and fungal data interpretation*

Principal component	Eigenvalue	Variance (%)	Cumulative variance (%)
Gram-negative bacteria			
PC-1	10.61	37.90	37.902
PC-2	8.22	29.36	67.26
PC-3	3.09	11.03	78.29
PC-4	1.72	6.13	84.42
Gram-positive bacteria			
PC-1	10.46	37.37	37.37
PC-2	8.25	29.48	66.85
PC-3	3.07	10.96	77.81
PC-4	1.72	6.15	83.95
Fungi			
PC-1	10.48	37.45	37.45
PC-2	8.39	29.95	67.40
PC-3	3.16	11.28	78.68
PC-4	1.72	6.142	84.82

Scree plots:

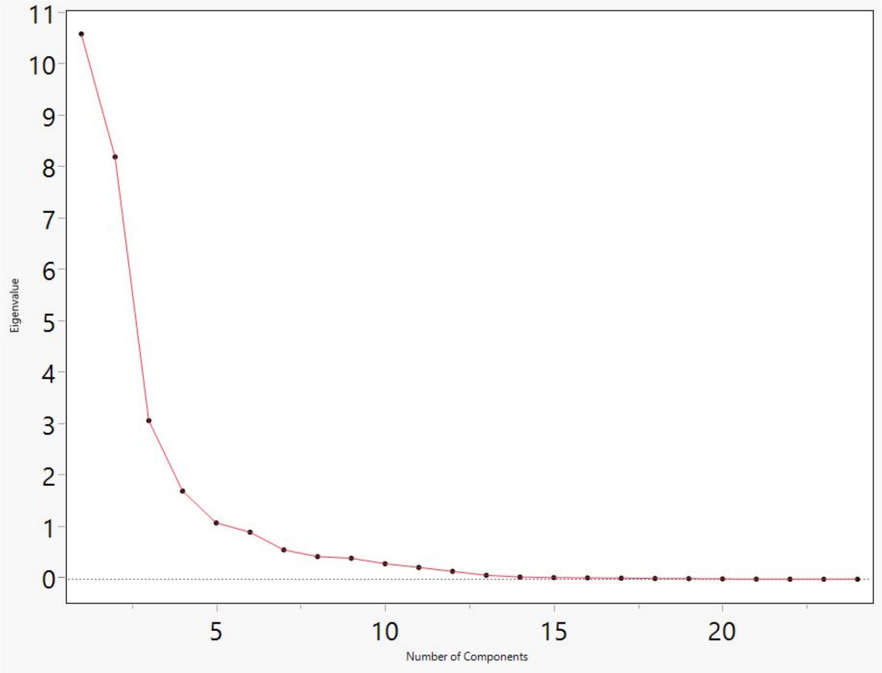

Figure S7: Scree plot for Gram-negative PCA showing the number of PCs with eigenvalues greater than 1.5. *PC-1, PC-2, PC-3, and PC-4 show eigenvalues greater than 1.5 and thus qualify to be used for the interpretation of results.*

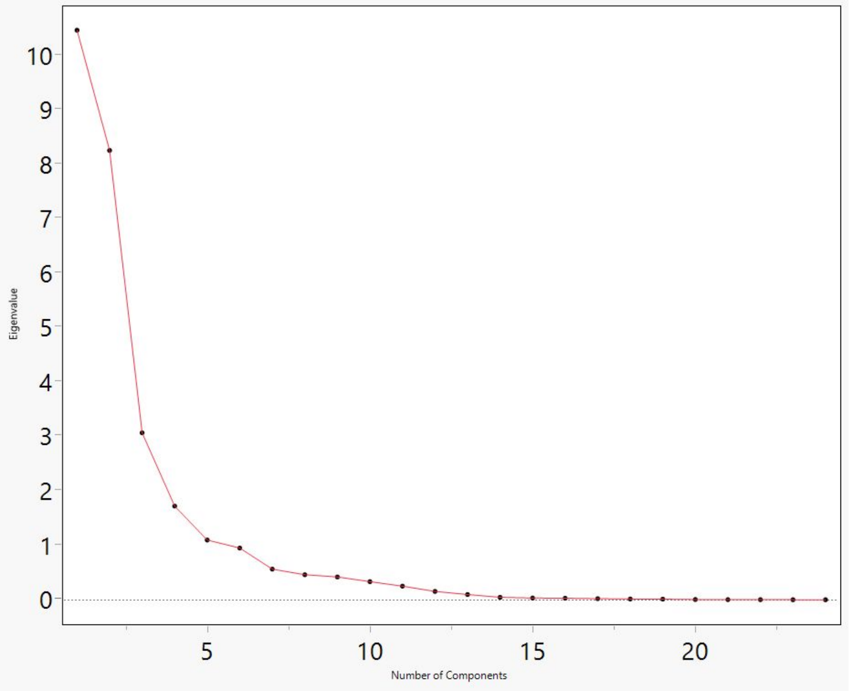

Figure S8: Scree plot for Gram-positive PCA showing the number of PCs with eigenvalues greater than 1.5. *PC-1, PC-2, PC-3, and PC-4 show eigenvalues greater than 1.5 and thus qualify to be used for the interpretation of results.*

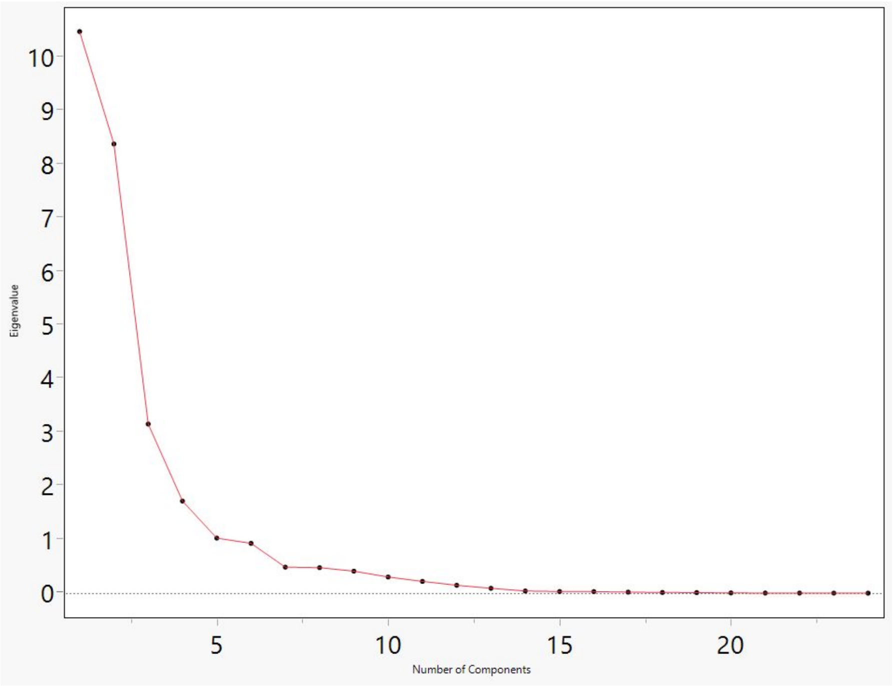

Figure S9: Scree plot for fungal PCA showing the number of PCs with eigenvalues greater than 1.5. *PC-1, PC-2, PC-3, and PC-4 show eigenvalues greater than 1.5 and thus qualify to be used for the interpretation of results.*

7. QSAR

***Table S15**: Correlation of single molecular descriptors of the 46 AMPs with antimicrobial and cytotoxic activities – Lipophilicity parameters determined with the Wimley-White bilayer/water scale at pH 7.0*

	**Property**	**R^2**				
		**Gram-negative bacteria MIC_{avg}**	**Gram-positive bacteria MIC_{avg}**	**Fungi MIC_{avg}**	**%Haemolysis**	**%HaCat cell death**
1	ElutionTime (mins)	-0.7203	-0.8032	-0.4590	0.5567	0.6485
2	$SCSA_N$	-0.7492	-0.7920	-0.4968	0.5230	0.6082
3	**L_N**	**-0.6213**	**-0.4232**	**-0.2635**	**0.4503**	**0.5828**
4	**L**	**-0.1027**	**-0.1691**	**0.0327**	**0.3917**	**0.5214**
5	MwN	-0.6832	-0.7462	-0.4162	0.4500	0.4865
6	CD Structure	-0.7250	-0.5670	-0.3425	0.4597	0.4548
7	**L_P/L_N**	**-0.2107**	**-0.1939**	**0.0976**	**0.2000**	**0.3794**
8	**L/L_N**	**-0.2107**	**-0.1939**	**0.0976**	**0.2000**	**0.3794**
9	CSL	-0.5204	-0.5914	-0.3447	0.3155	0.2755
10	SCSA	-0.4589	-0.3225	-0.1024	0.0341	0.2350
11	CAR	-0.0190	-0.0051	0.1287	0.0498	0.2250
12	**L_P/L**	**-0.1072**	**-0.0870**	**-0.1665**	**0.1140**	**0.2182**
13	Mw	-0.3691	-0.2877	-0.1111	0.0576	0.1195
14	Sequence length	-0.3623	-0.3182	-0.0633	0.0297	0.0748
15	**L_P**	**0.5005**	**0.2365**	**0.3458**	**0.0272**	**0.0531**
16	#RotBonds	-0.3049	-0.1062	-0.0739	-0.0619	0.0321
17	Q/CAR	-0.0162	0.1919	0.1001	-0.2490	-0.1772
18	**Q/L**	**0.0765**	**0.0361**	**0.0935**	**-0.0877**	**-0.1908**
19	HBacc	0.0344	0.1853	0.0936	-0.2188	-0.2010
20	$SCSA_P$	0.0653	0.2597	0.2801	-0.3813	-0.2144
21	HBtot	0.0447	0.1872	0.0260	-0.1794	-0.2305
22	HBdon	0.0486	0.1828	-0.0091	-0.1543	-0.2389
23	MwP	0.1253	0.2805	0.2178	-0.3133	-0.2675
24	Charge	0.0364	0.2892	0.0388	-0.2451	-0.2776
25	Q/CSL	0.3733	0.4251	0.3222	-0.3037	-0.3021
26	$SCSA_P/SCSA_N$	0.4631	0.5194	0.4496	-0.4156	-0.3158
27	MwP/MwN	0.4330	0.5075	0.3644	-0.3871	-0.3630
28	**Q/L_N**	**0.3642**	**0.3229**	**-0.0081**	**-0.2258**	**-0.3787**
